# Stereoselective Synthesis of Novel Sphingoid Bases Utilized for Exploring the Secrets of Sphinx

**DOI:** 10.3390/ijms22158171

**Published:** 2021-07-29

**Authors:** Essa M. Saied, Christoph Arenz

**Affiliations:** 1 Institute for Chemistry, Humboldt Universität zu Berlin, Brook-Taylor-Str. 2, 12489 Berlin, Germany; saiedess@hu-berlin.de; 2 Chemistry Department, Faculty of Science, Suez Canal University, Ismailia 41522, Egypt

**Keywords:** sphingolipids, 1-deoxysphingolipids, sphingoid bases, branched sphingosine, deuterated sphingolipids, SPT selectivity, stereoselective synthesis, cross metathesis

## Abstract

Sphingolipids are ubiquitous in eukaryotic plasma membranes and play major roles in human and animal physiology and disease. This class of lipids is usually defined as being derivatives of sphingosine, a long-chain 1,3-dihydroxy-2-amino alcohol. Various pathological conditions such as diabetes or neuropathy have been associated with changes in the sphingolipidome and an increased biosynthesis of structurally altered non-canonical sphingolipid derivatives. These unusual or non-canonical sphingolipids hold great promise as potential diagnostic markers. However, due to their low concentrations and the unavailability of suitable standards, the research to explore the secret of this class of ‘Sphinx’ lipids is ultimately hampered. Therefore, the development of efficient and facile syntheses of standard compounds is a key endeavor. Here, we present various chemical approaches for stereoselective synthesis and in-depth chemical characterization of a set of novel sphingoid bases which were recently utilized as valuable tools to explore the metabolism and biophysical properties of sphingolipids, but also to develop efficient analytical methods for their detection and quantification.

## 1. Introduction

**Figure 1:**
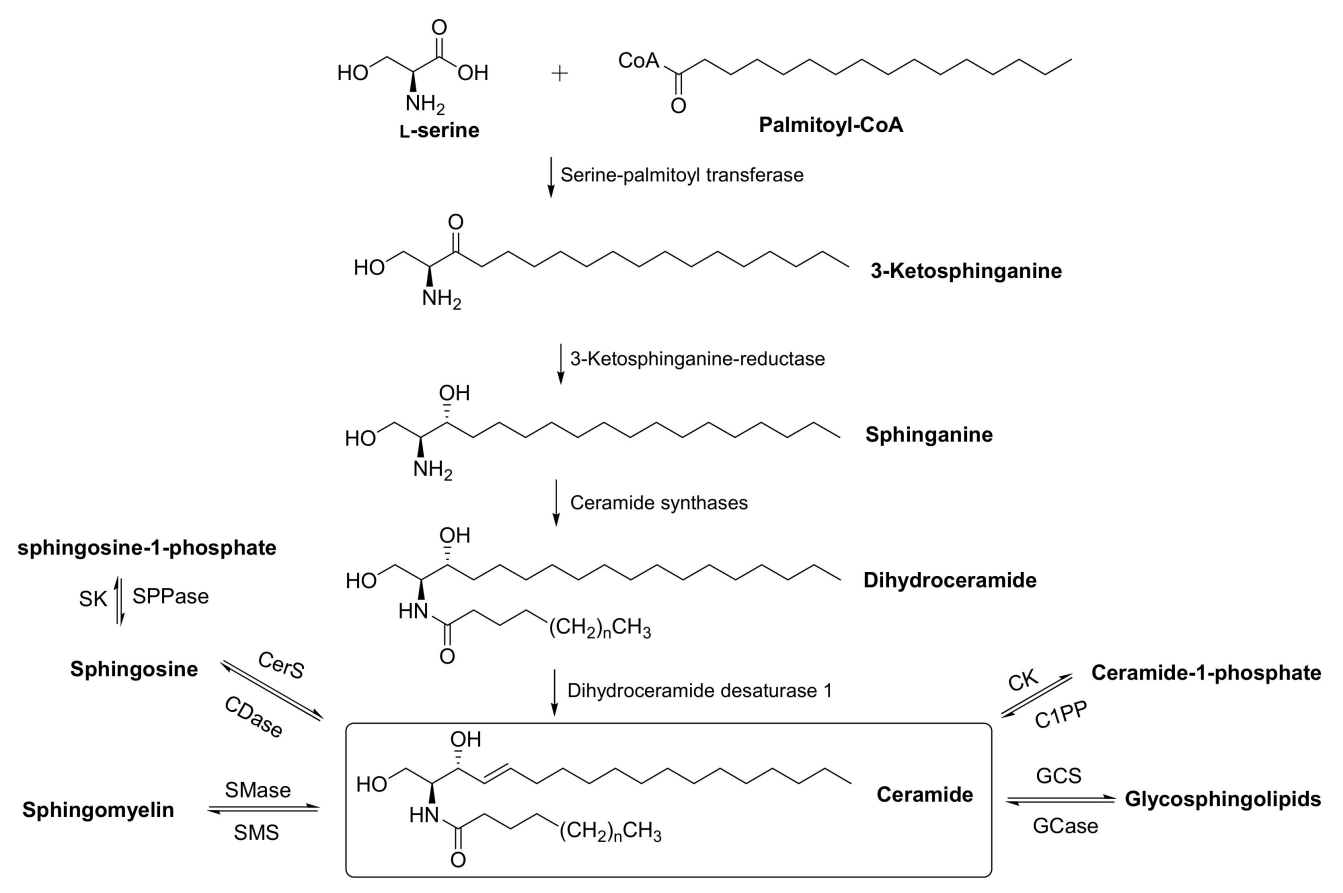
Metabolism of canonical sphingolipids. Various different headgroups can be added to ceramide to form different classes of complex sphingolipids. SMS, sphingomyelin synthase; SMase, sphingomyelinase; GCS, glucosyl-ceramide synthase; GCase, glucosyl ceramidase; CDase, ceramidase; CerS, ceramide synthase; CK, ceramide kinase; C1PP, ceramide-1-phosphate phosphatase; SPPase, S1P phosphatase; SK, sphingosine kinase.

Sphingolipids (SL) are major components of all eukaryotic plasma membranes and characterized by hydroxyl amino alkenes, also termed long-chain bases (LCB), as a core structural motif [1,2,3]. A key step for the synthesis of LCBs is the condensation reaction of L-serine and palmitoyl-CoA catalyzed by serine palmitoyl transferase (SPT) giving rise to C-18 3-ketosphinganine, which is then reduced to sphinganine (also termed “dihydrosphingosine”) followed by *N*-acylation and desaturation at the 4 position to form ceramides (Figure 1) [4]. The latter serves as membrane anchor for more complex SL such as glycosphingolipids (GSL) or the major plasma membrane lipid, sphingomyelin (SM) [1,5]. Under physiological conditions, about 60% of all mammalian sphingolipids contain C-18 sphingosine as core LCB. The remaining LCB differ with respect to chain length, saturation and hydroxylation and other features [6]. In other species such as plants and fungi (including yeast), the fully saturated 1,3,5-trihydroxyl-2-amino-octadecane, phytosphingosine, is the major LCB, which also occurs in mammals, but only in rather small amounts [7].

In the past, the head groups and acyl chains of more complex SL have been subject to intense research, while other modifications have been much less appreciated [6]. Most of the known variations to the LCB part of sphingolipids are introduced downstream of the SPT reaction. This is especially true for unique modifications found in some pathogenic fungi, which are the result of atypical biosynthetic pathways, catalyzed by specialized enzymes, which might provide opportunities for targeted pharmacological strategies [6,8,9].

**Figure 2:**
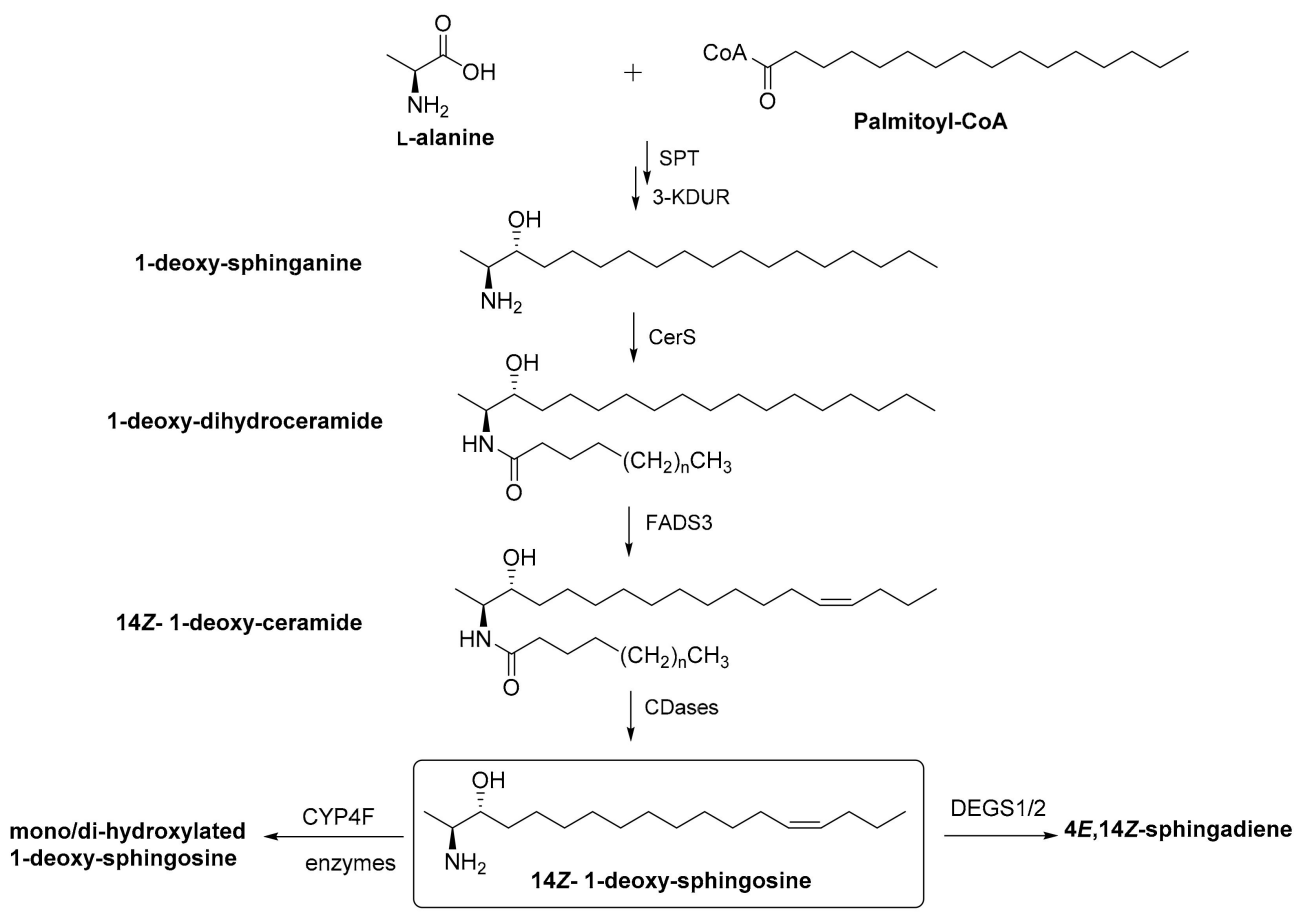
Metabolism of non-canonical sphingolipids. SPT enzyme can utilize L-alanine as a substrate, instead of L-serine, which leads to the formation of a family of atypical sphingolipid metabolites. The exact metabolic pathway of this lipid family is still elusive. SPT, serine palmitoyl transferase; 3-KDHR, 3-keto-reductase; CerS, ceramide synthases; FADS3, fatty acid desaturase 3; CDase, ceramidase; DEGS1/2, desaturase 1/2; CYP4F, cytochrome P450 (CYP)4F enzymes.

Recently, a number of LCB based on an alternative specificity of the SPT enzyme have come into focus. Research in this direction has been significantly triggered by the finding that the rare inherited neuropathy HSAN1, which is linked to mutations in the SPT gene, is correlated with highly elevated levels of LCBs devoid of the primary hydroxyl group (Figure 2) [10,11]. The resulting 1-deoxysphingolipids and 1-deoxymethyl sphingolipids are elevated due to a permanent shift in substrate preference of SPT from the canonical serine to alanine or glycine, respectively. Later it was shown that these lipids are not only neurotoxic, but also elevated in other diseases without mutations in the SPT gene, as in type II diabetes mellitus [12,13]. The mechanisms that underlie these changes in LCB composition under pathological conditions are still unknown, but subject to intense research. However, it was recently reported that serine supplementation significantly reduces the level of these atypical LCB leading to the amelioration of several diseases [14,15,16,17].

We have recently elucidated the chemical structure of 1-deoxysphingosine, the major LCB derived from an alanine instead of serine incorporation into the SPT reaction. We revealed that the primary misguided metabolic step is followed by an unexpected non-canonical introduction of the double bond. Instead of the 4*E*, a 14*Z* double bond was introduced [18]. It has been found that this double bond is formed by the desaturase FADS3, which is also responsible for introduction of the 14*Z* double bond in sphingadienine [19,20]. Furthermore, the alternative catabolism of this atypical LCB by members of the cytochrome P450 family and the resulting metabolites has been elucidated (Figure 2) [21]. Notably, the metabolic intermediates that have characterized during these studies have the potential to serve as potential biomarkers, not only for diseases such as HSAN1, but may also be highly predictive for the occurrence of neuropathy in diabetic patients [22]. Recently, the restricted metabolism of deoxysphingolipids was successfully employed for fluorescent “dead end” metabolic probes [23,24].

Several modified sphingoid bases have been recently discovered [2,22]. However, the exploration of their structures and their implication as biomarkers in pathological diseases is ultimately hampered by the unavailability of suitable standards and/or feasible efficient synthetic strategies. Herein, we describe multiple approaches for the synthesis of a novel set of sphingoid bases, which have been used as a valuable biochemical tool in the course of deciphering the mechanisms of canonical and non-canonical sphingolipid biosynthesis. Most of the presented compounds have been confirmed as naturally occurring metabolites or are currently under detailed investigations.

## 2. Results and Discussions

### 2.1. Synthesis of Novel Atypical Sphingoid Bases

As mentioned above, 1-deoxysphingolipids have been associated with several human diseases including anoxia-associated injuries, HSAN1, and diabetic sensory neuropathy [10,25,26]. Additionally, they have been recognized as potential plasma markers for predicting type 2 diabetes [22]. Therefore, the identification and quantification of 1-deoxysphingolipids and their metabolites have been critically highlighted. However, detection and characterization of 1-deoxysphingolipids are mainly hampered by the unavailability of suitable standards, and additionally, the absence of efficient and facile analytical techniques which could resolve the different kind of 1-deoxysphingolipid isomers (both structural and stereoisomers). Although 1-deoxy-sphingolipids are apparently a simple structure, their determination and structural elucidation rely on sophisticated coupled-analytical techniques [27,28,29,30,31]. Based on these facts, we aimed at synthesizing a set of 1-deoxy-sphingoid bases (**6**,**9**) which could be used as a valuable tool to study the 1-deoxysphingolipids metabolism and to establish efficient analytical techniques which allow their detection and quantification.

#### 2.1.1. 5*E*-1-Deoxy-sphingosine

**Scheme 1:**
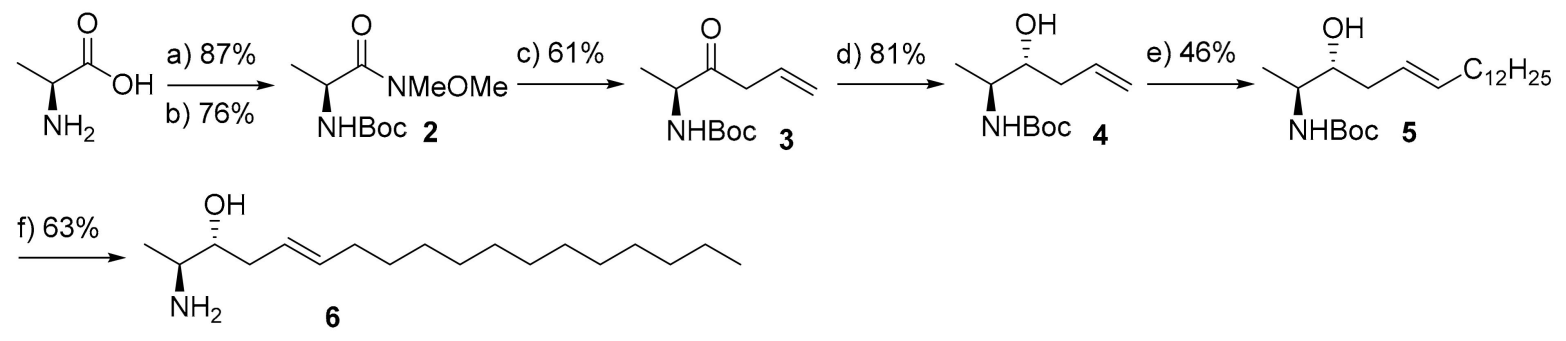
Synthesis of 5*E*-1-deoxy-sphingosine (**6**). Reagents and conditions: (**a**) Boc_2_O, 1 M NaOH, dioxane, −10 °C to r.t., 4 h; (**b**) Me(MeO)NH.HCl, EDCI.HCl, NMM, DCM, −15 °C, 2 h; (**c**) (i) Mg, 1,2-DBE, 1-allylbromide, Et_2_O, 3 h, (ii) **2**, Et_2_O, 0 °C- r.t., 3 h; (**d**) TBLAH, EtOH, −78 °C, 2 h; (**e**) 1-tetradecene, *p*-benzoquinone (10 mol%), CuI (2 mol%), Grubbs 2nd generation cat. (10 mol%), *d*-chloroform, r.t., 12 h; (**f**) AcCl, MeOH, 0 °C-r.t., 2 h.

During our study to establish an analytical technique for resolving the structure of 1-deoxy-sphingolipid isomers, we intended to investigate the applicability of cryogenic gas-phase infrared spectroscopy to differentiate between 1-deoxy-sphingolipid isomers with different C=C bond positions and/or different function groups [32]. Accordingly, we envisioned synthesizing 5*E*-1-deoxy-sphingosine **6** and 6*E*-3-keto-1-deoxy-sphingosine **9** as valuable standards. The synthesis of 5*E*-1-deoxy-sphingosine **6** was envisioned following our previously developed route in which the cross-coupling metathesis was the key reaction step (Scheme 1) [33,34,35]. Thus, L-alanine was reacted with Boc_2_O to provide *N*-Boc-L-alanine which was subsequently activated to the corresponding Weinreb amide to afford compound **2** in a 63% yield (over 2-steps). Subsequent reaction of compound **2** with freshly prepared allylmagnesium bromide under standard conditions formed the respective α,β-unsaturated ketone **3** in a moderate yield. Stereoselective reduction of allyl ketone **3** was successfully achieved using LiAl[O*t*-Bu]_3_-H to afford the desired *anti*-isomer **4** as a sole product in an excellent yield (81%, *dr* 98%). The *anti*-isomer **4** was then subjected to olefin cross-coupling metathesis with 1-tetradecene using Grubbs 2nd generation cat. under standard conditions to provide the protected 1-deoxy sphingoid base **5** with the desired *E*-configuration as the major product (71% yield) [36,37,38,39,40].

However, a careful check of NMR and LC-MS analysis revealed that the obtained product **5** was contaminated with a side-product with M-14 which is produced due to migration of the double bond by 1,3-H shifts during the cross-metathesis reaction. Purification of this mixture of product was not possible by standard purification techniques. Although, olefin migration is a well-documented side reaction occurring in Ru-catalysed olefin metathesis, in particular when using second-generation catalysts, [41,42,43,44,45] we were surprised by the extend of this side reaction in our case. We hypothesized that this problem occurred due to the Ru-catalyzed isomerization of the double bond in **5** to the allyl-position, since the tendency of 1-tetradecene to isomerize to 2-tetradecene should be comparatively low (not electron deficient). Several studies reported the influence of additives to suppress this undesirable reaction [43,45,46,47]. In our hands, we found that using Grubbs 2nd generation cat. in the presence of 1,4-benzoquinone (10 mol%) and CuI (2 mol%) afforded the desired product **5** in moderate yield with good *E*-selectivity and, most importantly, without any isomerization side-product detected (Table S1). Finally, deprotection of the Boc-group upon treatment with acetyl chloride furnished the desired compound **6**. This probe was used, together with our previously synthesized isomers [34], as valuable standards to establish an efficient analytical method for resolving the structure of 1-deoxy-sphingolipid isomers using cryogenic gas-phase infrared spectroscopy [32].

#### 2.1.2. 6*E*-3-Keto-1-deoxy-sphingosine

**Scheme 2:**
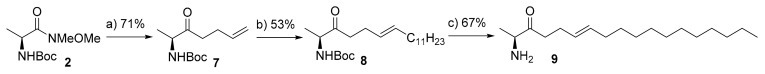
Synthesis of 6*E*-3-keto-1-deoxy-sphingosine (**9**). Reagents and conditions: (**a**) (i) Mg, 1,2-DBE, 4-bromo-1-butene, Et_2_O, 1 h, (ii) **2**, Et_2_O, 0 °C- r.t., 2 h; (**b**) 1-tridecene, *p*-benzoquinone (10 mol%), CuI (2 mol%), Grubbs 2nd generation cat. (10 mol%), *d*-chloroform, 40 °C, 3 h; (**c**) AcCl, MeOH, 0 °C-r.t., 2 h.

Next, we intended to synthesize 6*E*-3-keto-1-deoxy-sphingosine **9**. The synthesis was achieved by a 3-steps synthetic route similar to that described for compound **6** (Scheme 2). Thus, protected Weinreb amide **2** was reacted with 4-butenylmagnesium bromide to provide the truncated 3-keto-1-deoxy-shingosine **7** in a 71% yield. Subsequently, compound **7** was subjected to an olefin cross-coupling metathesis with 1-tridecene under our modified conditions (CuI 2 mol%, *p*-benzoquinone 10 mol%) in order to avoid the undesired olefin isomerization. Fortunately, the desired *E*-isomer **8** was selectively formed (*E*/*Z* 10:1) in a good yield without any isomerization product detected (as judged by LC-MS and NMR analysis). Deprotection of **8** was achieved with acetyl chloride to afford the target compound 6*E*-3-keto-1-deoxy-sphingosine **9** with a total yield of 25%. Compound **9** has been used to advocate the power of gas-phase IR spectroscopy to provide distinguished spectroscopic fingerprints for saturated and unsaturated 1-deoxysphinolipids (**9**, 3-keto-1-deoxysphinganin), but also for functional 1-deoxysphingolipid isomers (**9**, 1-deoxysphingosine). In addition, compound **9** could be used as a valuable biochemical tool to investigate whether the desaturation step also occurs in non-canonical sphingolipids after *N*-acylation or is independent from this step [22,48].

#### 2.1.3. Atypical Sphingoid Bases for Exploring Biophysical Properties of Non-Canonical Sphingolipids

**Scheme 3:**
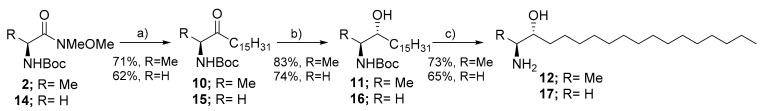
Synthesis of 1-deoxy-sphinganine (**12**) and 1-deoxymethyl-sphinganine (**17**). Reagents and conditions: (**a**) (i) Mg, 1,2-dibromoethane, 1-bromopentadecane, THF, 2–3 h. (ii) **2** or **14**, THF, 0 °C- r.t., 2–3 h; (**b**) TBLAH, EtOH, −78 °C, 2–3 h; (**c**) AcCl, MeOH, 0 °C-r.t., 2 h.

Changes in membrane biophysical properties have been related to the biological actions of sphingolipids and may contribute to pathological diseases [2,22]. As previously described, the non-canonical sphingoid bases lack the primary hydroxyl group (1-deoxy-sphingoid bases) or the hydroxymethyl group (1-deoxymethyl-LCBs). The influence of these lipids on the lipid–lipid interactions and their impact on the biochemical and biophysical properties of membranes are still unexplored [49,50]. In this regard, we envisioned 1-deoxysphinganine **12** and 1-deoxymethyl-sphinganine **17** as useful biochemical tools to follow up on these questions. Several approaches have been successfully reported for the diastereoselective synthesis of the antitumor 1-deoxy-sphinganine (+-spisulosine) starting from d-isoascorbic acid [51], chiral auxiliary [52,53], asymmetric chiral diols [54,55] and Garner aldehyde [56]. Although these routes succeeded to efficiently obtain (+)-spisulosine **12** in enantiopure form, they suffer from multiple steps route, low stereo-selectivity, low yield, or expensive starting materials. Here, we intended to utilize L-alanine as a chiral starting material in order to take the advantage of already established chiral center of the final scaffold. Indeed, *N*-protected L-alaninal has been previously applied as an efficient chiral starting material for facile synthesis of (+)-spisulosine **12** [57,58,59]. In these approaches, the *N*-protected L-alaninal was diastereoselectively reacted with metalated -pentadecyne (Grignard form or Hydrozirconation form), followed by hydrogenation and deprotection steps to afford enantiopure (+)-spisulosine **12**. In our route, we sought to modify these approaches for a more facile and efficient synthesis of deoxy-sphinaganine scaffold (Scheme 3). In this regard, we activated *N*-Boc-L-alanine to the corresponding Weinreb amide form **2**. The latter was then efficiently reacted with pentadecylmagnesium bromide, instead of an unsaturated organic nucleophile, to afford *N*-Boc-3keto-sphinganine **10** in a 71% yield.

The key challenge here was the diastereoselective reduction to the desired *anti*-aminol diastereomer. For this purpose, we have screened several reducing agents and reaction conditions (Table S2). In our hands, both NaBH_4_ and DIBAL-H afforded the desired product in moderate yield but with low diastereoselectivity (*anti*/*syn* 7:4–1:1). The best diastereoselectivity (*anti*/*syn* 76:24) was obtained when the reaction was performed in ethanol using LiAl[O*t*-Bu]_3_-H as reducing agent. Finally, deprotection of the amino group using acetyl chloride furnished (+)-spisulosine **12** in a 73% yield. The diastereopurity of compound **12** was confirmed by comparing the analytical data to the reported one. Following this established route, the synthesis of compound **17** was successfully performed starting from *N*-Boc-glycine to afford 1-deoxymethyl-sphinganine in 4 steps. Notable, we did not deeply investigate the enantiopurity of the obtained product **17**, since the stereo-configuration effect of the secondary hydroxyl group was not the main interest of this compound. Having compounds **12** and **17** in hands, we have recently investigated the influence of these naturally occurring atypical sphingoid bases on the biophysical properties of lipid membranes. Our studies have revealed that these atypical lipids, even in very small concentrations, significantly affect the intramolecular interactions of membrane lipids and impair membrane fluidity [49,50]. Future studies should investigate whether the change of the membrane properties by these lipids may contribute to their pathobiological role [60].

### 2.2. Synthesis of Deuterated Sphingoid Bases

#### 2.2.1. 14*Z*-1-Deoxy-sphingosine-*d*_2_

**Scheme 4:**
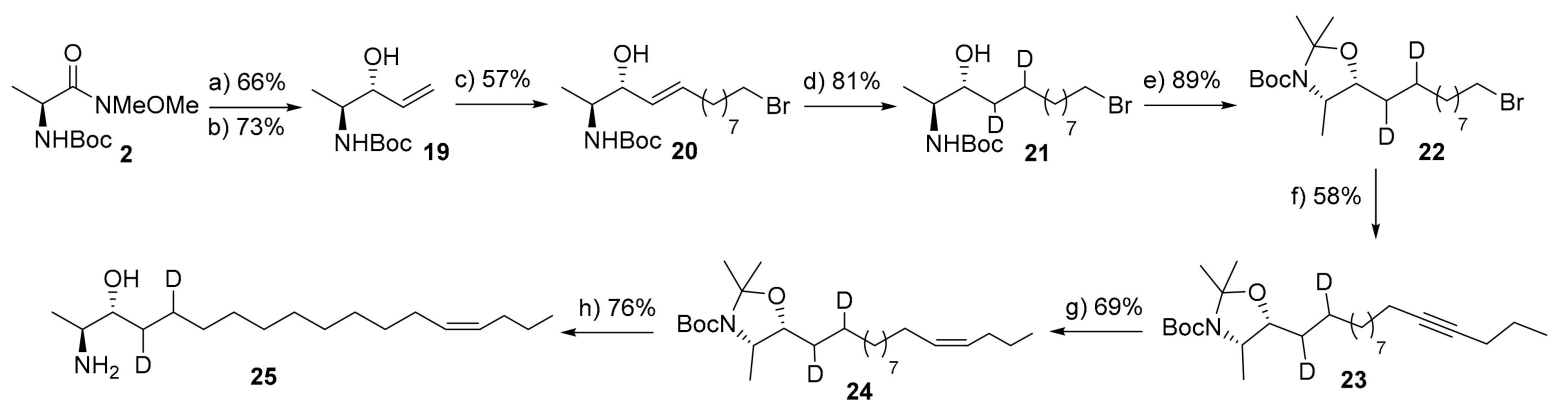
Synthesis of 14*Z*-1-deoxy-sphingosine-*d*_2_ (**25**). Reagents and conditions: (**a**) vinylmagnesium bromide (1 M in THF), THF, −20 °C-r.t., 4 h; (**b**) TBLAH, EtOH, −78 °C, 3 h; (**c**) 10-bromo-1-decene, *p*-benzoquinone (10 mol%), Grubbs 2nd generation cat. (10 mol%), *d*-chloroform, 40 °C, 12 h; (**d**) D_2_, 10% Pd/C, *d*-acetic acid (cat.), *d*-Methanol, r.t., 14 h; (**e**) 2,2-dimethoxypropane, *p*-TsOH (cat.), toluene, reflux, 2 h; (**f**) (i) 1-pentyne, *tert*-BuLi, THF, −78 °C, (ii) **22**, HMPA, THF, −78 °C- r.t.,12 h; (**g**) H_2_, Lindlar cat., EtOAc, DMF, r.t., 16 h; (**h**) AcCl, MeOH, 0 °C-r.t., 2 h.

Deuterated form of bioactive metabolites has been recognized as a potential biochemical tool for metabolic labeling approaches and for high-resolution MS/MS quantification approaches. The exact mechanism of 1-deoxysphingolipd metabolism is still elusive, however we recently elucidated the structure of the native 1-deoxy-sphingosine as 14*Z*-1-deoxy-sphingosine [18]. Furthermore, we showed that 14*Z*-1-deoxy-sphingosine can be metabolized to a set of eight 1-deoxysphingolipid downstream metabolites through reactions regulated by P450 (CYP)4F enzymes [21]. To deeply investigate the metabolic pathways of 1-deoxysphingolipids, we aimed at synthesizing a deuterated standard of 14*Z*-1-deoxysphingosine **25**. Towards this end, we have developed a synthetic approach which allows for the establishment both of deuterium and 14-*cis* double bond (Scheme 4). The synthesis was started from *N*-Boc-L-alanine which was converted into the truncated 1-deoxysphingosine **19** in 3-steps following our previous report [33,34]. Subsequent cross-coupling metathesis of intermediate **19** with 10-bromo-1-decene cross partner in the presence of Grubbs 2nd generation cat. (10 mol%) and *p*-benzoquinone (10 mol%) successfully provided the desired ω-bromo-truncated deoxysphingosine **20** in moderate yield (*E*/*Z* 11:2, no isomerization was observed). Compound **20** was then subjected to a deuteration process using catalytic 10% Pd/C and *d*-acetic acid under positive pressure of D_2_ to afford the corresponding deuterated ω-bromo- deoxysphingosine **21** in an 81% yield.

Next, both the hydroxyl group and amino group were protected by reaction with dimethoxy propane to provide intermediate **22**. The latter was then reacted with lithiated 1-pentyne in the presence of HMPA (as a cosolvent) to furnish compound **23** in moderate yield. Stereoselective reduction to the desired *Z*-configuration was successfully achieved by hydrogenation of compound **23** in the presence of Lindlar catalyst to produce the protected *d*-14*Z*-deoxy-sphingosine **24** (no *E*-stereoisomer was observed). Finally, deprotection of all protecting groups under acidic conditions furnished the desired *d*-14*Z*-deoxy-sphingosine **25**. This compound has been used in several metabolic approaches to investigate the 1-deoxysphingolipid metabolism to unexplored the physiological roles of 1-deoxysphingolipids (unpublished data). Further, 1-deoxy-sphingosine has been highlighted as a potential biomarker for several human diseases including diabetic retinopathy, however, their detection and quantification are mainly based on the calibration curves of the native analyte since there are no standards available for the deuterated 1-deoxysphingolipids, Therefore, compound **25** would be a useful analytical tool as a mass standard for quantification of cellular 1-deoxy-sphingolipid levels [21].

#### 2.2.2. 3-Ketosphinganine-*d*_2_

**Scheme 5:**
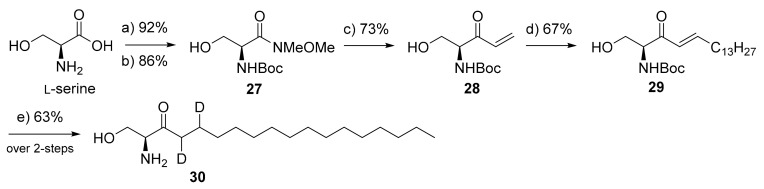
Synthesis of 3-ketosphinganine-*d*_2_ (**30**). Reagents and conditions: (**a**) Boc_2_O, 1 M NaOH, dioxane, −10 °C to r.t., 4 h; (**b**) Me(MeO)NH.HCl, EDCI.HCl, NMM, DCM, −15 °C, 2 h; (**c**) (i) *n*-BuLi, THF, −65 °C, 30 min, (ii) vinylmagnesium bromide (1 M in THF), −65 °C- r.t., 6 h; (**d**) 1-pentadecene, Grubbs 2nd generation cat.(7 mol%), DCM, 40 °C, 6 h; (**e**) (i) D_2_, 10% Pd/C, *d*-acetic acid (cat.), *d*-Methanol, r.t., 12 h, (ii) 4 M HCl, THF-MeOH, reflux, 2 h.

The regulatory mechanism for amino acid selectivity of SPT enzyme is unknown. In order to investigate SPT selection of amino acid substrates, it is important to be able to detect and measure the SPT-product (e.g., 3-ketosphinganine) in high accuracy since it is the direct metabolite generated. Therefore, a deuterated MS standard for the canonical 3-ketosphinganine **30** was envisioned as a valuable biochemical tool [61]. Towards this goal, a synthetic approach was developed similar to that previously reported by our group (Scheme 5) [33]. Thus, L-serine was converted into the respective *N*-Boc-protected Weinreb amide **27**. This was followed by reaction with vinylmagnesium bromide in the presence of *n*-BuLi to yield the vinyl ketone **28** in a 73% yield [62]. Compound **28** was then subjected to cross-coupling metathesis reaction with 1-pentadecen in the presence of Grubbs 2nd generation cat. (7 mol%) to afford the 3-keto-sphingosine **29** in moderate stereoselectivity (*E*/*Z* 7:2). Notably, compound **29** has been previously synthesized with high stereoselectivity from *N*-Boc-*O*-TBS L-serine by copper(I)-mediated coupling with *E*-1-pentadecenyl boronic acid [63].

Since the configuration of the double bond is not essential, the mixture of *E*/*Z* **29** was used in the deuteration step. Thus, *E*/*Z* **29** was exposed to D_2_ in the presence of a catalytic 10% Pd/C to provide the protected *d*-3-keto-sphinganine which was treated with 4 M HCl-THF solution to finally furnish the target molecule *d*-3-keto-sphinganine with a 63% yield (over 2-steps). Compound **30** has been used in an intensive study as a mass standard probe to explore the role of *Tsc3* in modulating amino acid selectivity of SPT in *Saccharomyces cerevisiae* [61].

### 2.3. Synthesis of Naturally Occurring Typical Sphingoid Bases

#### 2.3.1. 16*S*-Methyl-sphingosine

**Scheme 6:**
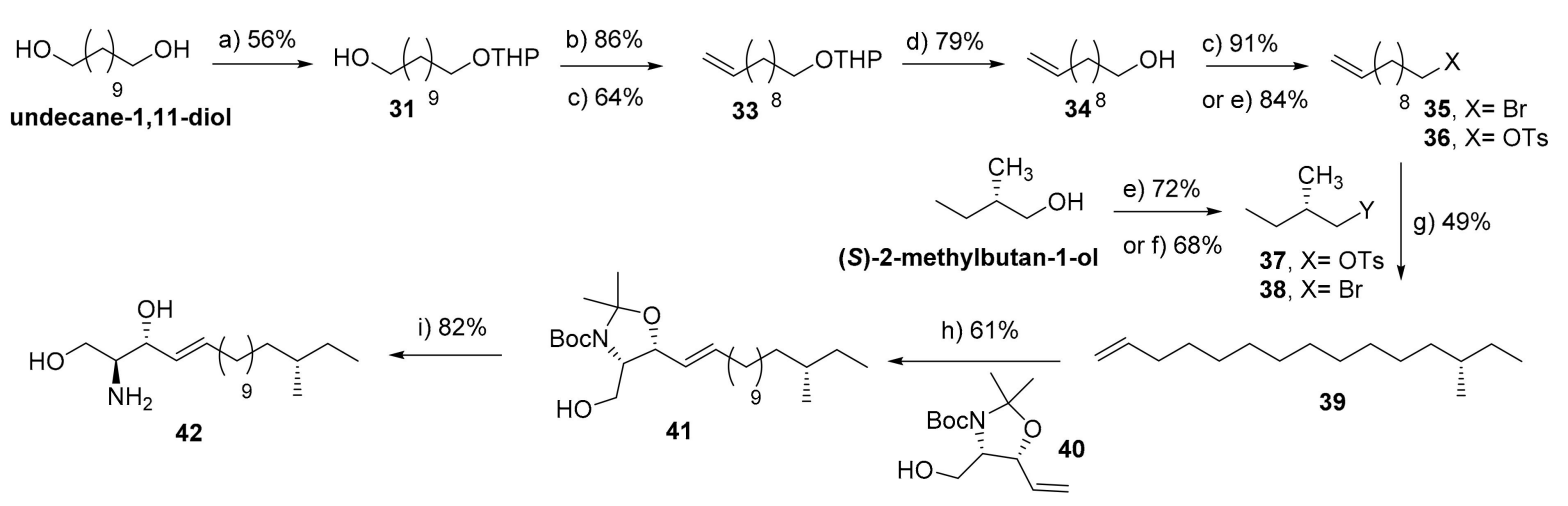
Synthesis of (16*S*)-methyl-sphingosine (**42**). Reagents and conditions: (**a**) DHP, PTSA (cat.), THF, 0 °C-r.t., 16 h; (**b**) CBr_4_, PPh_3_, DCM, 0 °C-r.t., 4 h; (**c**) t-BuOK, THF, reflux, 12 h; (**d**) PPTS (cat.), EtOH, 62 °C, 2 h; (**e**) TsCl, pyridine, DCM, 0 °C-r.t., 16 h; (**f**) NBS, PPh_3_, DCM, 0 °C-r.t., 2 h; (**g**) (i) **38**, Mg, THF, 30 °C, 90 min, and then (ii) **43**, Li_2_CuCl_4_ (cat), THF, −78 °C-r.t., 16 h; (**h**) **40**, Grubbs 2nd generation (cat.), DCM, reflux, 12 h; (**i**) AcCl, MeOH, 0 °C-r.t., 2 h.

We recently identified 16*S*-methyl-sphingosine as the main product of the subunit SPTLC3 reaction through the condensation of *anteiso*-methyl-palmitate with L-serine [64]. To proof this finding and to in-deep investigate its metabolism, we envisioned the synthesis of the methyl-branched LCB **42**. As shown in Scheme 6, the synthesis of **42** was depicted via cross-coupling metathesis of the 13*S*-pentadecene **39** and the protected aminodiol derivative **40**. The latter was obtained in 7-steps from L-serine with a overall yield of 21% [33]. The key challenge in the synthesis of **42** was to obtain a stereisomer pure alkene **39**. To this end, we decided to start the synthesis from a chiral auxiliary (*S*)-2-methylbutanol. Initially, we have followed the previously reported route [65]. Thus, (*S*)-2-methylbutanol was treated with tosyl chloride in pyridine to afford the corresponding tosylated derivative **37** in a good yield. On the other hand, the 11-bromo-undecene **35** was synthesized in 5-steps starting from 1,11-undecandiol [34]. Briefly, the long-chain terminal diol was selectively mono-protected with a THP group as described above. The resulting alcohol **31** was brominated via Appel reaction followed by Br-elimination to yield the terminal alkene **33**. After acidic deprotection of the THP group, the obtained hydroxyl alkene **34** was subjected to Appel reaction to afford 11-bromo-undecene **35** in a quantitative yield. Subsequently, the 11-bromo-undecene **35** was converted to the corresponding Grignard form via reaction with magnesium metal. Unfortunately, coupling of tosylated alcohol **37** to the freshly prepared 11-undecenylmagnesium bromide in the presence of catalytic Li_2_Cu_2_Cl_4_ following the reported conditions [65,66] provided the desired 13*S*-methyl-petadecene **39** in a very low yield (less than 20%). Several trials have been performed in order to improve the yield of the desired product (reactant equivalents, catalyst amount, reaction time, etc.), however did not provide any significant effect. Furthermore, the NMR-spectra of the obtained product revealed that compound **39** is contaminated with 11-undecene, which could be formed via hydrolysis of the Grignard form of compound **35**.

Since the separation of these alkenes’ mixture was quite impossible via standard column chromatography and the possibility of 11-undecene to interfere with the subsequent cross-metathesis step, we decided to modify the coupling strategy. Thus, we thought to invert the role of the reagents, where the chiral (*S*)-2-methylbutyl bromide is used as a nucleophile (in the form of Grignard reagent) which would after hydrolysis lead to formation of (*S*)-2-methyl-butane. The latter could be removed easily under reduced pressure and would not interfere with the cross-metathesis reaction. To this end, (*S*)-2-methylbutanol was successfully treated with *N*-bromosuccinimide and triphenyl phosphine to provide the corresponding bromo derivative **38**, which was subsequently reacted with magnesium metal to form the respective (*S*)-2-methylbutylmagnesium bromide. The latter was reacted with the tosylated alkene **36** in the presence of 10 mol% Li_2_Cu_2_Cl_4_ to fortunately furnish the desired alkene **39** in 49% yield. Having the branched alkene **39** in hand, a cross-coupling metathesis reaction was performed with intermediate **40** in the presence of Grubbs 2nd generation catalyst to afford the desired *E*-protected branched-sphingosine **41** as the major product (*E*:*Z* 13:2). Finally, the protecting groups were removed by treatment with acetyl chloride to furnish 16*S*-methyl-sphingosine **42** in 82%. The structure was confirmed by NMR and LC-MS analysis. 16*S*-methyl-sphingosine **42** has been used as an MS-standard lipid to elucidate the structure of the natively formed sphingosine by SPTLC3 subunit. Additionally, this compound showed to be metabolized into the corresponding ceramide form and more complex sphingolipid metabolites. Further studies are under progress to investigate the effect of this branched lipid on the biochemical and biophysical properties of the membranes and their implications on the dermal pathologies [64].

#### 2.3.2. (2*S*,3*R*,14*Z*)-Sphingosine

Since the structure of 1-deoxysphingosine was elucidated as 14*Z*- instead of postulated 4*E*, it was hypothesized that the corresponding metabolite could be also formed in the canonical sphingolipids (i.e., 14*Z*-sphingosine). This hypothesis was supported by a recent study which showed that under mutation of DES1, the cells are still able to generate sphingosine which has different chromatographic behavior than the canonical 4*E*-isomer [67]. These findings indicate that there is another desaturase enzyme which can desaturate both typical and atypical sphingoid bases, but also that the formed sphingosine could be 14*Z* instead of 4*E*. Toward this end, we aimed at synthesizing 14*Z*-sphingosine to elucidate the structure of natively generated sphingosine under these pathological conditions [68], but also to investigate the biochemical and biophysical property of this metabolite.

**Scheme 7:**
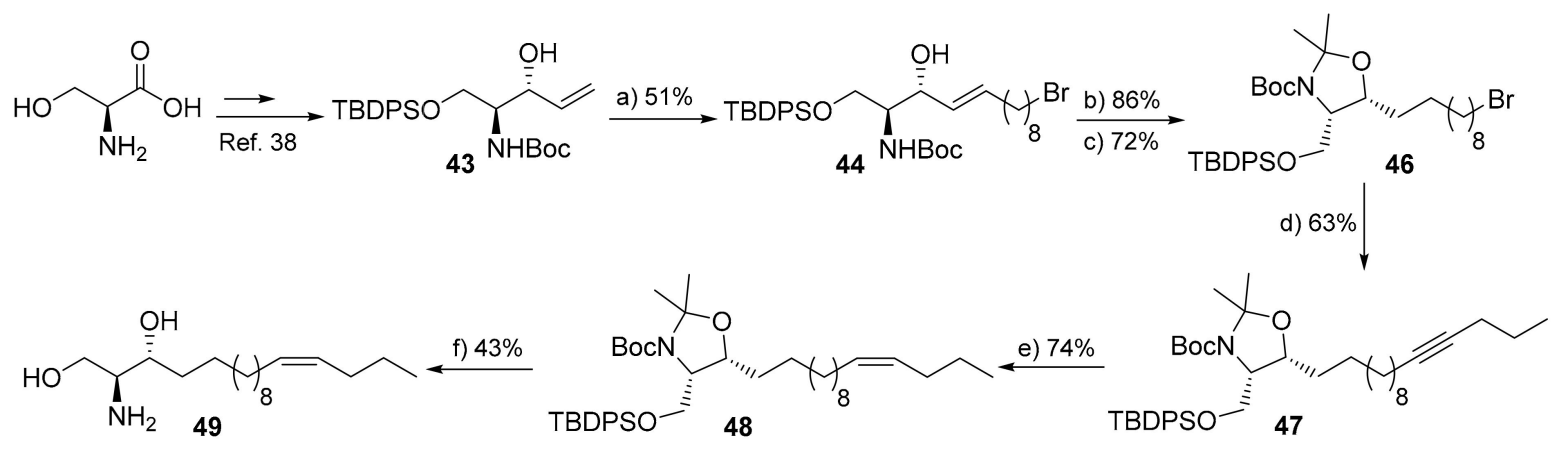
Synthesis of 14*Z*-sphingosine (**49**). Reagents and conditions: (**a**) 10-bromo-1-decene, *p*-benzoquinone (10 mol%), CuI (3 mol%), Grubbs 2nd generation cat. (10 mol%), *d*-chloroform, 35 °C, 14 h; (**b**) H_2_, 10% Pd/C, acetic acid (cat.), THF, r.t., 16 h; (**c**) 2,2-dimethoxypropane, *p*-TsOH (cat.), toluene, 80 °C, 3 h; (**d**) (i) 1-pentyne, *tert*-BuLi, THF, −78 °C, 2 h, (ii) **46**, HMPA, THF, −78 °C- r.t.,14 h; (**e**) H_2_, Lindlar cat., EtOAc, DMF, r.t., 12 h; (**f**) (i) TBAF, THF, 55 °C, 3 h, (ii) AcCl, MeOH, 0 °C-r.t., 3 h.

We initially attempted to follow our reported synthetic route for the synthesis of the 1-deoxy-analogue of the targeted molecule, starting from *O*-TBDPS-*N*-Boc L-serine (Scheme S1) [18]. Unfortunately, when the respective Weinreb amide of protected serine was treated with 11*Z*-pentadecenylmagnesium bromide under reported conditions, the desired protected 3-keto-14*Z*-sphingosine was only obtained in very low yield (>10%). Attempts to improve the yield (temperature, additives, Grignard equivalents, changing *O*-protecting group), did not afford any enhancement. Accordingly, we thought to start from the well-known intermediate **43**, since both stereocenters are already established, and to make use of the cross-coupling metathesis to construct the lipophilic tail (Scheme 7). Toward this end, intermediate **43**, obtained from L-serine in 5-steps [33], was subjected to cross-metathesis reaction with 10-bromo-decene under modified conditions to afford the truncated bromo-sphingosine **44** in a moderate yield and without any isomerization detected (*E*/*Z* 9:1, 51%). Hydrogenation of the double bond in *E*/*Z* **44** and subsequent acetal protection provided the corresponding fully protected bromo-sphinganine **46**. The latter was treated with lithiated pentyne in the presence of HMAP to provide intermediate **47** in a 63% yield. Stereoselective reduction to the *Z*-isomer was achieved by subjecting compound **47** to a hydrogenation reaction in the presence of Lindlar catalyst to afford the protected 14*Z*-sphingosine **48** as a main product (*E*/*Z* 20:1, 74%). Deprotection of **48** was proceeded in two subsequent steps, treatment with TBAF followed by acidic deprotection, to furnish the target molecule 14*Z*-sphingosine **49** in a 43% yield (over 2-steps).

With compound **49** in hands, the structure of natively formed sphingosine under DES1 mutation was elucidated as 14*Z*-sphingosine (not published). Interestingly, two independent groups recently found that the fatty acid desaturase 3 (FADS3) is responsible for introducing the double bond at 14*Z*-position for both typical and atypical sphingolipids [19,20]. Loss of DES1 activity has been recently reported as the main cause of hypo-myelinating *leukodystrophy* and has been implicated in the degeneration of nervous systems [67,68]. These findings suggest that 14*Z*-sphingosine or its metabolites could be related to these pathological diseases. Further studies are under progress to shed the light on the biophysical and molecular function of this lipid.

## 3. Conclusions

Sphingolipid levels vary greatly in the context of diseases. Currently, atypical sphingolipids have received great attention due to their implication in several pathological diseases, although their relatively low concentrations. Recently, it has become clear that SPT-catalyzed reaction is a source of multiple LCBs with high diagnostic potential, regardless of whether the underlying gene is mutated or not. With the synthetic standard compounds in combination with new sophisticated mass spectrometric methods, numerous isobars can be reliably analyzed and aligned with clinical parameters. Here we presented various chemical approaches for the synthesis of a novel set of sphingoid bases. The synthesized sphingoid bases were already utilized to deeply investigate this class of lipids in different aspects including biochemical, biophysical, and analytical aspects. We are convinced that the effectiveness of developed synthetic approaches will enable the synthesis of many typical and atypical sphingolipid standards that will result in exploring more secrets of ‘Sphinx’ and will ultimately stimulate research on the pathological role of sphingolipids.

## 4. Materials and Methods

### 4.1. General Description of Materials and Methods

Unless otherwise specified, all the commercially reagents were purchased from TCI (Berlin, Germany), Sigma-Aldrich (Berlin, Germany), Acros (Berlin, Germany) or Fluka (Berlin, Germany) and used without further purification. Solvents were distillated by standard methods before use. Thin-layer chromatography was performed on 60-F_254_ silica gel on aluminum sheets (Merck KGaA) and spots were detected by UV illumination (254 nm), and/or spraying with 1.3% ninhydrin solution, Seebach solution or KMnO_4_ solution followed by heating. Flash column chromatography was performed using Silica Gel 60 M (0.04–0.063 mm) as stationary phase with indicated eluent systems in parenthesis following the description of purification. Solvent ratios for chromatography and R_f_ values are reported in *v*/*v%* ratios. ^1^H, and ^13^C spectra were recorded on Bruker ADVANCE III 500 spectrometers and AVANCE 400 Bruker (^1^H at 300, 400 or 500 MHz, and ^13^C at 101.2 or 125.7 MHz) as solutions in CDCl_3_, CD_3_OD or mixtures of those at 25 °C. Chemical shifts (δ) are reported in parts per million (ppm): multiplicities are indicated as s (singlet), d (doublet), t (triplet), q (quartet), q = quartet; dd = doublet of doublets, ddd = doublet of doublet of doublets, dt = doublet of triplets, qd = quartet of doublets, m (multiplet) and br (broad). Chemical shifts are given in ppm with respect to the residual protons of the deuterated solvent (d-chloroform: δ 7.27 ppm; d-methanol: δ 3.31 ppm, unless otherwise noted). The coupling constants *J* are given in Hz. ^13^C NMR chemical shifts are expressed in parts per million (ppm) and referenced to the residual solvent peak (*d*-chloroform: δ 77.16 ppm; *d*-methanol δ 49.05 ppm, unless otherwise indicated). The optical rotation activity of the final compounds was measured on Jasco DIP-370 digital polarimeter equipped with a sodium D line lamp (*λ* = 589 nm). The optical rotation [α]_D_ was reported at ambient temperature for a certain sample concentration (*c*, g/100 mL, with a sample path length of 100 mm) and the value is given in 10^−1^ deg cm^2^ g^−1^ unit. UPLC−MS analyses were performed on an AGILENT 6120 UPLC-MS system (Santa Clara, CA, USA) consisting of an SQD (single quadrupole detector) mass spectrometer equipped with an electrospray ionization interface (ESI) and a photodiode array detector (DAD). The detection was performed in full scan mode and the major observable molecular ion and selected fragments, and clusters have been reported.

### 4.2. Synthetic Procedures and Analytical Data

#### 4.2.1. (2*S*,3*R*,*E*)-*tert*-Butyl-(3-hydroxyoctadec-5-en-2-yl)carbamate (**5**)

To a stirred solution of compound **4** (210 mg, 1 mmol) and 1-tetradecene (0.8 g, 4 mmol) in dry d-chloroform (5 mL) under argon atmosphere at ambient temperature was added *p*-benzoquinone (10 mol%) and CuI (2 mol%) followed by a catalytic amount of Grubbs 2nd generation catalyst (10 mol%). The resulting reaction mixture was allowed to stir at ambient temperature for 12 h (as monitored by TLC analysis, no change in the composition of reaction mixture, Pet. ether/ EtOAc 4:1; R*_f_* _(adduct)_ = 0.42; R*_f_* _(product)_ =0.56; visualized with 1.3% ninhydrine). The mixture was concentrated under reduced pressure and the resultant residue was purified by flash column chromatography over silica gel using petroleum ether and ethylacetate as eluents (0–10 % ethylacetate in petroleum ether) to provide the desired product **5** as colorless oil.

Yield: 184 mg (46 %). R*_f_*: 0.56 (Pet. ether /EtOAc 4:1, visualized with 1.3% ninhydrin solution). ^1^H NMR (CDCl_3_, 500 MHz, ppm) δ 5.55 (dt, *J* = 13.6, 6.7 Hz, 1H), 5.44–5.37 (m, 1H), 4.78 (br.s, 1H), 3.74–3.65 (m, 1H), 3.65–3.60 (m, 1H), 2.22–2.15 (m, 1H), 2.09 (dd, *J* = 15.0, 7.2 Hz, 1H), 2.00 (dd, *J* = 14.4, 7.2 Hz, 2H), 1.44 (s, 9H), 1.37–1.24 (m, 20H), 1.10 (d, *J* = 6.8 Hz, 3H), 0.88 (t, *J* = 7.0 Hz, 3H). ^13^C NMR (CDCl_3_, 126 MHz, ppm) δ 155.71, 134.8, 125.5, 79.4, 73.48, 50.1, 37.2, 32.7, 31.9, 29.7, 29.6, 29.6, 29.5, 29.4, 29.4, 29.2, 28.4, 22.7, 14.1. ESI-MS *m*/*z* calcd for C_23_H_45_NO_3_Na [M + Na]^+^ 406.33; observed: 406.3.

#### 4.2.2. (2*S*,3*R*,*E*)-2-Aminooctadec-5-en-3-ol (**6**)

To a stirred solution of compound **5** (135 mg, 0.36 mmol) in dry methanol (4 mL) at 0 °C was added dropwise acetyl chloride (260 µL, 3.6 mmol) over a period of 10 min. After being stirred at the same conditions for 30 min and for additional 2 h at ambient temperature (as indicated by TLC analysis for complete deprotection), the mixture was concentrated *in vacuo*. The residue was diluted with diethylether and washed with sat. NaHCO_3_ solution. The aqueous layer was extracted again with diethylether and the combined organic layers were washed with brine, dried over anhydrous Na_2_SO_4_, filtered and concentrated under reduced pressure. The obtained residue was purified by flash column chromatography over silica gel using isopropanol and ethylacetate as eluents (0–10% isopropanol in ethylacetate) to afford the pure product **6** as a white waxy-white solid.

Yield: 64 mg (63%). R*_f_*: 0.45 (EtOAc/ iso-propanol 4:1, visualized with 1.3% ninhydrin). ${\left[\alpha \right]}_{D}^{20}$ = +3.2 (c = 0.65, MeOH). ^1^H NMR (MeOD, 500 MHz, ppm) δ 5.58 (dd, *J* = 14.3, 7.5 Hz, 1H), 5.44 (dd, *J* = 14.6, 7.2 Hz, 1H), 3.78–3.72 (m, 1H), 3.26 (dd, *J* = 6.7, 2.8 Hz, 1H), 2.29–2.13 (m, 2H), 2.03 (dd, *J* = 13.3, 6.6 Hz, 2H), 1.42–1.27 (m, 20H), 1.23 (d, *J* = 6.8 Hz, 3H), 0.90 (t, *J* = 6.7 Hz, 3H). ^13^C NMR (MeOD, 126 MHz, ppm) δ 135.4, 126.2, 71.7, 51.9, 37.7, 33.67, 33.0, 30.8, 30.7, 30.6, 30.4, 30.4, 23.8, 14.5, 11.6. ESI-MS: *m*/*z* calcd for C_18_H_38_NO [M + H]^+^ 284.29; observed 284.3 [32].

#### 4.2.3. (*S*,*E*)-*tert*-Butyl-(3-oxooctadec-6-en-2-yl)carbamate (**8**)

A stirred solution of compound **7** (110 mg, 0.47 mmol) and 1-tridecene (0.34 g, 1.9 mmol) in dry d-chloroform (4 mL) under argon atmosphere at ambient temperature was treated a catalytic amount of *p*-benzoquinone (10 mol%) followed by CuI (2 mol%) and Grubbs catalyst 2nd generation (10 mol%). After the resulting reaction mixture was stirred under reflux for 6 h (as monitored by TLC analysis; no further change in the composition of the reaction mixture; Pet. ether/ EtOAc 4:1; R*_f_* _(adduct)_ = 0.55; R*_f_* _(product)_ =0.68; visualized with 1.3% ninhydrin), the solvent was removed under reduced pressure. The obtained residue was subjected to flash column chromatography over silica gel using petroleum ether and ethylacetate as eluents (0–10% ethylacetate in petroleum ether) to afford the desired product **8** as a waxy-white solid.

Yield: 95 mg (53 %). R*_f_*: 0.47 (Pet. ether /EtOAc 9:1, visualized with 1.3% ninhydrine solution). ^1^H NMR (CDCl_3_, 500 MHz, ppm) δ 5.52–5.34 (m, 1H), 5.33–5.25 (m, 1H), 5.20 (br.s, 1H), 4.35–4.20 (m, 1H), 2.59–2.41 (m, 1H), 2.28–2.18 (m, 1H), 1.99–1.84 (m, 4H), 1.37 (s, 9H), 1.30–1.17 (m, 21H), 0.81 (t, *J* = 7.0 Hz, 3H). ^13^C NMR (CDCl_3_, 126 MHz, ppm) δ 209.1, 155.2, 132.0, 127.85, 8.7, 55.1, 39.17, 32.51, 31.9, 29.6, 29.52, 29.4, 29.3, 29.2, 28.3, 26.6, 22.7, 17.9, 14.1. ESI-MS *m*/*z* calcd for C_23_H_43_NO_3_Na [M + Na]^+^ 404.31; observed: 404.3.

#### 4.2.4. (*S*,*E*)-2-Aminooctadec-6-en-3-one (**9**)

To a stirred solution of compound **8** (77 mg, 0.2 mmol) in absolute methanol (2 mL) at 0 °C was dropwisely added acetylchloride (143 µL, 2 mmol) over a period of 10 min. The resulting reaction mixture was allowed to stir at the same conditions for 30 min, gradually warm to ambient temperature, and stirred for additional 2 h; during which a white solid formed. The mixture was filtered, and the residue was washed several times with ice-cooled diethylether to afford a white waxy-solid of crude product. The obtained crude product was further purified by flash column chromatography over silica gel using ethylacetate and isopropanol as eluents (0–10% isopropanol in ethylacetate) to provide the final pure product **9** as a white solid.

Yield: 38 mg (67%). R*_f_*: 0.51 (EtOAc/iso-propanol 4:1, visualized with KMnO_4_ solution). ${\left[\alpha \right]}_{D}^{20}$ = +13.1 (c = 1.1, MeOH). ^1^H NMR (MeOD, 500 MHz, ppm) δ 5.55–5.47 (m, 1H), 5.45–5.39 (m, 1H), 4.14 (q, *J* = 7.3 Hz, 1H), 2.76–2.69 (m, 1H), 2.66–2.58 (m, 1H), 2.35–2.27 (m, 2H), 1.98 (q, *J* = 6.6 Hz, 2H), 1.53–1.49 (m, 4H), 1.37–1.27 (m, 17H), 0.90 (t, *J* = 7.0 Hz, 3H). ^13^C NMR (MeOD, 126 MHz, ppm) δ 206.8, 133.1, 129.1, 55.9, 39.3, 33.6, 33.1, 30.8, 30.7, 30.6, 30.5, 30.3, 27.2, 23.7, 15.7, 14.4. ESI-MS: *m*/*z* calcd for C_11_H_36_NO [M + H]^+^ 282.28; observed 282.3 [32].

#### 4.2.5. (*S*)-*tert*-Butyl-(3-oxooctadecan-2-yl)carbamate (**10**)

The 1-pentadecylmagnesium bromide solution was synthesized as follows; A mixture of magnesium turnings (0.51 g, 21 mmol) in anhydrous THF (4 mL) under an argon atmosphere at ambient temperature was treated with drops of 1,2-dibromoethane. The resulting mixture was allowed to stir at the same conditions for 20 min, before it was treated dropwisely with a solution of 1-bromopentadecane (1.5 g, 5.2 mmol, in 2 mL of anhydrous THF) over a period of 20 min. The resulting reaction mixture was allowed to stir for additional 3 h at 35 °C to afford a transparent solution of 1-pentadecylmagnesium bromide which was immediately used in the next step.

A stirred solution of Weinreb amide derivative **2** (0.35 g, 1.5 mmol) in anhydrous THF (15 mL) under an argon atmosphere at 0 °C was treated dropwisely with a freshly prepared 1-pentadecylmagnesium bromide solution (6 mL, 5.1 mmol, 1 M solution in Et_2_O) over a period of 10 min. After being stirred at ambient temperature for 2 h (as monitored by TLC analysis; Pet. ether / EtOAc 4:1; R*_f_* _(adduct)_ = 0.2; R*_f_* _(product)_ =0.69; visualized with 1.3% ninhydrin solution), the reaction mixture was cooled again to 0 °C and subsequently quenched with an ice-cold 1 M HCl solution (40 mL). The resulting mixture was diluted with diethylether (50 mL), and the layer were separated. The aqueous layer was extracted with diethylether (3 × 50 mL), and the combined organic phases were washed with saturated NaHCO_3_ solution (80 mL) and brine (80 mL), dried over anhydrous Na_2_SO_4_, filtered and concentrated under reduced pressure. The resultant crude product was purified by flash column chromatography over silica gel using petroleum ether and ethylacetate as eluents (from 5–15% ethylacetate in petroleum ether) to yield compound **10** as white solid.

Yield: 410 mg (71 %). R*_f_*: 0.48 (Pet. ether /EtOAc 9:1, visualized with 1.3% ninhydrine solution). ^1^H NMR (CDCl_3_, 500 MHz, ppm) δ 5.28 (d, *J* = 6.0 Hz, 1H), 4.30 (p, *J* = 7.0 Hz, 1H), 2.55–2.40 (m, 2H), 1.62–1.54 (m, 2H), 1.43 (s, 9H), 1.31 (d, *J* = 7.2 Hz, 3H), 1.29–1.21 (m, 24H), 0.87 (t, *J* = 7.0 Hz, 3H). ^13^C NMR (CDCl_3_, 126 MHz, ppm) δ 209.9, 155.2, 79.6, 55.0, 39.2, 31.9, 29.7, 29.7, 29.6, 29.4, 29.4, 29.2, 28.3, 23.6, 22.7, 18.0, 14.1. ESI-MS *m*/*z* calcd for C_23_H_46_NO_3_ [M + H]^+^ 384.34; observed: 384.3.

#### 4.2.6. (2*S*,3*R*)-*tert*-Butyl-(3-hydroxyoctadecan-2-yl)carbamate (**11**)

To a stirred solution of compound **10** (0.34 g, 0.89 mmol) in dry ethanol (2 mL) under argon atmosphere at −78 °C was added portionwise lithium tri-(*tert*-butoxy)-aluminum hydride (0.57 g, 2.2 mmol) over a period of 20 min. After the resulting reaction mixture was allowed to stir at the same conditions for 2 h (as judged by TLC analysis: Pet. ether / EtOAc 4:1; R*_f_* _(adduct)_ = 0.69; R*_f_* _(product)_ =0.57; visualized with 1.3% ninhydrine solution), an ice-cold 1 M HCl solution (100 mL) was added dropwise to quench the reaction. The resulting mixture was allowed to warm gradually to ambient temperature and subsequently diluted with ethylacetate (100 mL). The layers were separated and the aqueous layer was extracted again with ethylacetate (2 × 100 mL). The combined organic extracts were washed with sat. NaHCO_3_ solution and brine, dried over anhydrous Na_2_SO_4_, filtered, and concentrated under reduced pressure. Flash column chromatography for the obtained residue over silica gel using petroleum ether and ethylacetate as eluents (15–23% ethylacetate in petroleum ether) afforded the desired product **11** as a white solid.

Yield: 285 mg (83 %). R*_f_*: 0.57 (Pet. ether /EtOAc 4:1, visualized with 1.3% ninhydrin solution). ^1^H NMR (CDCl_3_, 500 MHz, ppm) δ 4.63 (br. s, 1H), 3.77–3.53 (m, 2 H), 1.46 (s, 9H), 1.45–1.22 (m, 28H), 1.07 (d, *J* = 6.8 Hz, 3H), 0.88 (t, *J* = 6.4 Hz, 3H). ^13^C NMR (CDCl_3_, 126 MHz, ppm) δ 155.8, 79.6, 74.6, 50.7, 33.6, 32.1, 29.8, 29.7, 29.6, 29.4, 28.5, 26.2, 22.7, 14.4, 14.1. ESI-MS *m*/*z* calcd for C_23_H_48_NO_3_ [M + H]^+^ 386.36; observed: 386.4.

#### 4.2.7. (2*S*,3*R*)-2-Aminooctadecan-3-ol (**12**)

A solution of compound **11** (0.15 g, 0.38 mmol) in dry methanol (4 mL) at 0 °C was treated with acetyl chloride (272 µL, 3.8 mmol) in dropwise over a period of 10 min. After the resulting reaction mixture was stirred at the same conditions for 30 min and for additional 1 h at ambient temperature (as controlled by TLC analysis; Pet. ether/EtOAc 3:2; R*_f_* _(adduct)_ = 0.51; R*_f_* _(product)_ = 0.0; visualized with 1.3% ninhydrin solution), the solvent was removed under reduced pressure. The obtained residue was portioned between diethylether (100 mL) and sat. NaHCO_3_ solution (90 mL). The layers were separated and the aqueous layer was extracted again with diethylether (2 × 80 mL). The combined organic layers were washed with brine, dried over anhydrous Na_2_SO_4_, filtered, and concentrated *in vacuo* to afford a white waxy solid. The crude product was further purified by flash column chromatography over silica gel using ethylacetate and isopropanol as eluents (0–10% isopropanol in ethylacetate) to provide the final product **12** as a white solid.

Yield: 84 mg (77%). R*_f_*: 0.47 (EtOAc/ iso-propanol 9:1, visualized with 1.3% ninhydrine). ${\left[\alpha \right]}_{D}^{20}$ = +8.4 (c = 1.2, MeOH); [ref +5.2 (c = 0.36, MeOH)] [53]. ^1^H NMR (MeOD, 500 MHz, ppm) δ 3.72–3.68 (m, 1H), 3.27 (qd, *J* = 6.8, 3.0 Hz, 1H), 1.53 (dt, *J* = 11.0, 8.2 Hz, 1H), 1.45 (dt, *J* = 13.6, 4.6 Hz, 2H), 1.38–1.27 (m, 26H), 1.22 (d, *J* = 6.8 Hz, 3H), 0.90 (t, *J* = 7.0 Hz, 3H). ^13^C NMR (MeOD, 126 MHz, ppm) δ 71.6, 52.6, 34.0, 33.1, 30.8, 30.8, 30.7, 30.7, 30.6, 30.5, 27.0, 23.7, 14.4, 12.0. ESI-MS: *m*/*z* calcd for C_18_H_40_NO [M + H]^+^ 286.32; observed 286.3 [32].

#### 4.2.8. *tert*-Butyl-(2-oxoheptadecyl)carbamate (**15**)

1-Pentadecylmagnesium bromide was synthesized as follows; A mixture of magnesium powder (0.51 g, 21 mmol) in dry THF (2 mL) under argon atmosphere was treated with a catalytic drop of 1,2-dibromoethane followed by a dropwise addition of a solution of 1-bromopentadecane (1.5 g, 5.2 mmol, 1 M in dry TFH). The resulting reaction mixture was stirred under reflux for 2 h to afford a transparent solution of 1-pentadecylmagnesium bromide which was used in the next step.

To a stirred solution of compound **14** (330 mg, 1.5 mmol) in dry THF (15 mL) under argon atmosphere at 0 °C was added dropwise a freshly prepared 1-pentadecylmagnesium bromide solution over a period of 20 min. After being stirred for 2 h at ambient temperature (as monitored by TLC analysis; Pet. ether/ EtOAc 4:1; R*_f_* _(adduct)_ = 0.19; R*_f_* _(product)_ = 0.68; visualized with 1.3% ninhydrine), the reaction mixture was added in dropwise to an ice-cooled 1 M HCl solution (100 mL) to quench. The resulting mixture was diluted with ethylacetate (100 mL) and the layers were separated. The aqueous layer was extracted with ethylacetate (2 × 100 mL), and the combined organic layers were washed with sat. NaHCO_3_ solution and brine, dried over Na_2_SO_4_, filtered, and concentrated. The obtained residue was purified by flash column chromatography over silica gel using petroleum ether and ethylacetate as eluents (0–14% ethylacetate in petroleum ether) to afford compound **15** as a white solid.

Yield: 340 mg (62%). R*_f_*: 0.47 (cyclohexane/EtOAc 9:1, visualized with 1.3% ninhydrin solution). ^1^H NMR (CDCl_3_, 500 MHz, ppm) δ 4.11 (br.s, 2H), 2.58–2.42 (m, 2H), 1.66–1.58 (m, 2H), 1.44 (s, 9H), 1.28–1.20 (m, 24H), 0.87 (t, *J* = 6.9 Hz, 3H). ^13^C NMR (CDCl_3,_ 126 MHz, ppm): δ 209.5, 155.3, 79.7, 42.6, 40.1, 33.2, 29.8, 29.6, 29.6, 29.5, 29.4, 29.3, 28.6, 23.6, 22.7, 14.2. ESI-LCMS: *m*/*z* calcd for C_22_H_43_NO_3_Na [M + Na]^+^ 392.31; observed 392.3.

#### 4.2.9. (*R*)-*tert*-Butyl-(2-hydroxyheptadecyl)carbamate (**16**)

Lithium tri-(*tert*-butoxy)-aluminium hydride (0.49 g, 1.9 mmol) was added in portionwise to a stirred solution of compound **15** (285 mg, 0.76 mmol) in dry ethanol (2 mL) under argon atmosphere at −78 °C. After the resulting reaction mixture was stirred at the same conditions for 3 h (as detected by TLC analysis; Pet. ether/ EtOAc 4:1; R*_f_* _(adduct)_ = 0.69; R*_f_* _(product)_ = 0.55; visualized with 1.3% ninhydrin), an ice-cooled 1 M HCl solution (100 mL) was added in dropwise over a period of 20 min. The resulting mixture was allowed to warm to ambient temperature and was extracted with ethylacetate (3 × 100 mL). The combined organic extracts were washed with sat. NaHCO_3_ solution and brine, dried over Na_2_SO_4_, filtered, and concentrated under reduced pressure. Purification of the resultant residue by flash column chromatography over silica gel using petroleum ether and ethylacetate as eluents (10–20% ethylacetate in petroleum ether) afforded the desired product **16** as a white waxy-solid.

Yield: 210 mg (74%). R*_f_*: 0.55 (cyclohexane/EtOAc 4:1, visualized with 1.3% ninhydrin solution). ^1^H NMR (CDCl_3,_ 500 MHz, ppm): δ ^1^H NMR (CDCl_3_, 500 MHz, ppm) δ 4.48 (br. s, 1H), 3.79–3.72 (m, 1H), 3.17–2.89 (m, 2H), 1.47 (s, 9H), 1.47–1.24 (m, 28H), 0.89 (t, *J* = 6.5 Hz, 3H). ^13^C NMR (CDCl_3,_ 126 MHz, ppm): δ 155.6, 79.5, 62.3, 43.7, 34.5, 32.6, 29.8, 29.7, 29.6, 29.4, 28.5, 26.3, 22.7, 14.1. ESI-LCMS: *m*/*z* calcd for C_22_H_46_NO_3_ [M + H]^+^ 372.35; observed 372.3.

#### 4.2.10. (*R*)-1-Aminoheptadecan-2-ol (**17**)

Acetyl chloride (305 µL, 4.3 mmol) was added in dropwise to a stirred solution of compound **16** (0.16 g, 0.43 mmol) in dry methanol (4 mL) at 0 °C. The resulting reaction mixture was allowed to stir at the same conditions for 30 min and for additional 2 h at ambient temperature (as monitored by TLC analysis), during while a white solid was formed. The mixture was filtrated, and the residue was washed several times with ice-cooled diethylether to afford a white waxy-solid crude product. Purification of the crude product by flash column chromatography over silica gel using isopropanol and ethylacetate as eluents (0–10% isopropanol in ethyacetate) provided the final product **17** as a white solid.

Yield: 76 mg (65%). R*_f_*: 0.39 (EtOAc/iso-propanol 10:1, visualized with 1.3% ninhydrin). ${\left[\alpha \right]}_{D}^{20}$ = +1.9 (c = 1.0, MeOH). ^1^H NMR (MeOD, 500 MHz, ppm) δ 3.77–3.70 (m, 1H), 3.01 (dd, *J* = 12.7, 3.0 Hz, 1H), 2.75 (dd, *J* = 12.7, 9.5 Hz, 1H), 1.48 (t, *J* = 7.8 Hz, 2H), 1.43–1.23 (m, 26H), 0.90 (t, *J* = 6.9 Hz, 3H). ^13^C NMR (MeOD, 126 MHz, ppm) δ 68.8, 46.1, 36.0, 33.1, 30.8, 30.7, 30.7, 30.6, 30.5, 26.4, 23.7. ESI-MS *m*/*z* clcd for C_17_H_38_NO [M + H]^+^: 272.29; observed 272.3 [32].

#### 4.2.11. (2*S*,3*R*)-*tert*-Butyl-(13-bromo-3-hydroxytridecan-2-yl-4,5-*d*_2_)carbamate (**21**)

To a stirred solution of compound **20** (660 mg, 1.7 mmol) in dry *d*-methanol (20 mL) under argon atmosphere at ambient temperature was added a catalytic amount of 10% Pd/C (10 mg) followed by catalytic drop of *d*-acetic acid. The reaction vessel was evacuated and backfilled with deuterium gas (this process was repeated 2 times) and the resulting heterogeneous reaction mixture was allowed to stir at the same conditions for 14 h (as detected by HPLC analysis for complete reduction reaction). The reaction mixture was filtered through a short pad of Celite, which was rinsed several times with methanol (3 × 50 mL). The combined filtrates were concentrated under reduced pressure and the obtained residue was purified by flash column chromatography over silica gel using cylcohexane and ethylacetate as eluents (10–20% ethylacetate in cyclohexane) to provide compound **21** as a colorless oil.

Yield: 540 mg (81%). R*_f_*: 0.5 (Pet. ether/ EtOAc 4:1, visualized with 1.3% ninhydrin solution). ^1^H NMR (CDCl_3,_ 500 MHz, ppm): δ 4.76 (s, 1H), 3.67 (d, *J* = 15.5 Hz, 1H), 3.63 (t, *J* = 6.3 Hz, 1H), 3.40 (t, *J* = 6.9 Hz, 2H), 1.88–1.81 (m, 2H), 1.44 (s, 9H), 1.41–1.22 (m, 14H), 1.07 (d, *J* = 6.8 Hz, 3H).^13^C NMR (CDCl_3,_ 126 MHz, ppm): δ 156.0, 79.6, 74.6, 50.7, 34.2, 33.6, 33.0, 29.8, 29.6, 29.6, 29.5, 28.9, 28.5, 28.3, 26.2, 14.5. ESI-LCMS: *m*/*z* calcd for C_18_H_35_D_2_NO_3_Br [M + H]^+^ 396.21; observed 396.2.

#### 4.2.12. (4*S*,5*R*)-*tert*-Butyl-5-(10-bromodecyl-1,2-*d*_2_)-2,2,4-trimethyloxazolidine-3-carboxylate (**22**)

To a stirred solution of compound **21** (515 mg, 1.3 mmol) in anhydrous toluene (13 mL) under an argon atmosphere was added 2,2-dimethoxypropane (0.8 mL, 6.5 mmol), followed by a catalytic amount of *p*-toluensulfonic acid monohydrate (10 mol%). After being stirred under reflux in a Dean-Stark apparatus for 2 h (as judged by the TLC analysis; Pet. ether/ EtOAc 4:1; R*_f_* _(adduct)_ = 0.5; R*_f_* _(product)_ = 0.78; visualized with 1.3% ninhydrin solution), the reaction mixture was cooled to ambient temperature, diluted with ethylacetate, and successfully quenched with sat. NaHCO_3_ solution (80 mL). The mixture was transferred to separating funnel and the layers were separated. The aqueous layer was extracted again with ethylacetate (2 × 80 mL) and the combined organic layers were washed with brine, dried over anhydrous Na_2_SO_4_, filtered and concentrated. Flash column chromatography of the resultant crude residue over silica gel using cyclohexane and ethylacetate as eluents (0% to 10% ethylacetate in cyclohexane) provided the desired product **22** as a colorless oil.

Yield: 505 mg (89%). R*_f_*: 0.53 (cyclohexane/EtOAc 9:1, visualized with 1.3% ninhydrin solution). ^1^H NMR (CDCl_3,_ 500 MHz, ppm): δ 3.99–3.76 (m, 2H), 3.40 (t, *J* = 6.9 Hz, 2H), 1.88–1.81 (m, 2H), 1.61–1.45 (m, 15H), 1.45–1.23 (m, 14H), 1.07 (dd, *J* = 15.9, 6.2 Hz, 3H). ^13^C NMR (CDCl_3,_ 126 MHz, ppm): δ 152.1, 151.8, 92.6, 92.2, 79.9, 79.3, 76.6, 76.3, 55.4, 55.4, 34.2, 33.0, 29.6, 29.5, 28.9, 28.7, 28.6, 28.3, 27.5, 25.1, 24.0, 14.5, 13.8. ESI-LCMS: *m*/*z* calcd for C_21_H_38_D_2_NO_3_BrNa [M+Na]^+^ 458.22; observed 458.2.

#### 4.2.13. (4*S*,5*R*)-*tert*-Butyl-2,2,4-trimethyl-5-(pentadec-11-yn-1-yl-1,2-*d*_2_)oxazolidine-3-carboxylate (**23**)

The lithated 1-pentyne was synthesized as follows; to a stirred solution of 1-pentyne (330 µL, 3.3 mmol) in anhydrous THF (7 mL) under an argon atmosphere at −78 °C was carefully added a solution of *t*-BuLi (1.8 mL, 3 mmol, 1.7 M solution in pentane) in dropwise over a period of 20 min; the rate of addition was adjusted to keep the internal temperature below −65 °C. The resulting reaction mixture was stirred at the same conditions for 2 h to afford lithiated 1-pentyne solution which was used directly in the next step.

A stirred solution of freshly prepared lithated 1-pentyne at −78 °C was sequentially treated with hexamethylphosphoramide (870 µL, 5 mmol), followed by a dropwise addition of a solution of compound **22** (470 mg, 1.1 mmol, 0.25 M solution in anhydrous THF) over a period of 20 min. After being stirred at the same conditions for 1 h, and for additional 12 h at ambient temperature (as monitored by TLC analysis; Pet. ether/ EtOAc 9:1; R*_f_* _(adduct)_ = 0.53; R*_f_* _(product)_ = 0.64; visualized with Seebach reagent), the reaction mixture was carefully quenched with an ice-cold sat. NH_4_Cl solution (80 mL). The resulting mixture was diluted with ethylacetate (80 mL) and the layers were separated. The aqueous phase was extracted again with ethylacetate, and the combined organic phases were subsequently washed with sat. NaHCO_3_ solution and brine, dried over anhydrous Na_2_SO_4_, filtered, and concentrated. The obtained residue was purified by flash column chromatography over silica gel using petroleum ether and ethylacetate as eluents (from 0–15% ethylacetate in petroleum ether) to afford the desired product **23** as a pale yellow oil.

Yield: 265 mg (58%). R*_f_*: 0.48 (Pet. ether/ EtOAc 10:1, visualized with 1.3% ninhydrine solution). ^1^H NMR (CDCl_3,_ 500 MHz, ppm): δ 3.93–3.74 (m, 2H), 2.66–2.27 (m, 4H), 1.78–1.64 (m, 2H), 1.63–1.46 (m, 17H), 1.44–1.22 (m, 14H), 1.09 (dd, *J* = 15.9, 6.1 Hz, 3H), 0.89 (t, *J*= 7.3 Hz, 3H). ^13^C NMR (CDCl_3,_ 126 MHz, ppm): δ 152.1, 151.8, 92.6, 92.2, 83.7, 79.9, 79.3, 77.8, 76.6, 76.4, 55.4, 55.4, 31.6, 30.3, 29.9, 29.7, 29.7, 29.7, 29.6, 29.4, 28.7, 28.6, 28.4, 27.5, 27.4, 25.1, 24.0, 23.0, 22.7, 22.1, 14.5, 14.0, 13.8. ESI-LCMS: *m*/*z* calcd for C_26_H_46_D_2_NO_3_Br [M + H]^+^ 424.38; observed 424.4.

#### 4.2.14. (4*S*,5*R*)-*tert*-Butyl-2,2,4-trimethyl-5-((*Z*)-pentadec-11-en-1-yl-1,2-*d*_2_)oxazolidine-3-carboxylate (**24**)

A stirred solution of compound **23** (220 mg, 0.53 mmol) in dry mixture of ethylacetate:DMF (50 mL, 95:5) under an argon atmosphere at ambient temperature was treated with a catalytic amount of Lindlar catalyst. The reaction vessel was evacuated and then backfilled with hydrogen gas (this process was repeated at least 2 times), and the resulting reaction mixture was allowed to stir at the same conditions for 16 h (as monitored by HPLC analysis for complete reduction). The mixture was subsequently filtered through a pad of Celite, which was rinsed several times with ethylacetate (3 × 80 mL). The combined filtrates were concentrated under *in vacuo* and the obtained residue was purified by flash column chromatography over silica gel using petroleum ether and ethylacetate as eluents (from 0–10% ethylacetate in petroleum ether) to provide the desired compound **24** as a colorless oil.

Yield: 155 mg (69%). R*_f_*: 0.47 (Pet. ether/ EtOAc 10:1, visualized with 1.3% ninhydrin solution). ^1^H NMR (CDCl_3,_ 500 MHz, ppm): δ 3 5.40–5.32 (m, 2H), 3.98–3.77 (m, 2H), 2.04–1.98 (m, 4H), 1.60–1.45 (m, 16H), 1.41–1.23 (m, 17H), 1.08 (dd, *J* = 16.1, 6.2 Hz, 3H), 0.90 (t, *J* = 7.4 Hz, 3H). ^13^C NMR (CDCl_3,_ 126 MHz, ppm): δ 152.1, 151.8, 130.2, 129.8, 92.6, 92.2, 79.9, 79.3, 76.6, 76.4, 55.5, 55.4, 31.6, 30.3, 29.9, 29.7, 29.7, 29.7, 29.6, 29.4, 28.7, 28.6, 28.4, 27.5, 27.4, 25.1, 24.0, 23.3, 23.0, 14.5, 14.0, 13.8. ESI-LCMS: *m*/*z* calcd for C_26_H_46_D_2_NO_3_Br [M + H]^+^ 426.39; observed 426.4.

#### 4.2.15. (2*S*,3*R*,*Z*)-2-Aminooctadec-14-en-4,5-*d*_2_-3-ol (**25**)

To a stirred solution of compound **24** (0.12 g, 0.28 mmol) in dry methanol (3 mL) at 0 °C was added dropwise acetyl chloride (200 µL, 2.8 mmol). After being stirred at the same conditions for 30 min, and for additional 90 min at ambient temperature (as monitored by TLC, visualized with KMnO_4_ solution), the reaction mixture was concentrated under reduced pressure. The resultant residue was taken up in diethylether (50 mL) and sequentially washed with saturated NaHCO_3_ solution (50 mL) and brine solution (80 mL), dried over anhydrous Na_2_SO_4_, filtered and concentrated *in vacuo*. Flash column chromatography of the obtained crude product over silica gel using ethylacetate and isopropanol as eluents (from 0–10% isopropanol in ethylacetate) provided the desired product **25** as a white solid.

Yield: 62 mg (76%). R*_f_*: 0.46 (EtOAc/ iso-propanol 4:1, visualized with 1.3% ninhydrin solution). ${\left[\alpha \right]}_{D}^{20}$ = +11.6 (c = 0.73, MeOH). ^1^H NMR (MeOD_,_ 500 MHz, ppm): δ 5.39–5.31 (m, 2H), 3.72–3.68 (m, 1H), 3.27 (qd, *J* = 6.8, 3.0 Hz, 1H), 2.06–1.99 (m, 2H), 1.46–1.28 (m, 18H), 1.22 (d, *J* = 6.8 Hz, 3H), 0.91 (t, *J* = 7.4 Hz, 3H). ^13^C NMR (MeOD_,_ 126 MHz, ppm): δ 131.0, 130.6, 71.6, 52.6, 30.8, 30.7, 30.6, 30.3, 30.3, 28.1, 23.9, 14.1, 12.0. ESI-LCMS: *m*/*z* calcd for C_18_H_36_D_2_NO [M + H]^+^ 286.31; observed 286.3.

#### 4.2.16. (*S*)-*tert*-Butyl-(1-hydroxy-3-oxopent-4-en-2-yl)carbamate (**28**)

To a stirred solution of compound **27** (1.45 g, 5.9 mmol) in dry THF (30 mL) at −78 °C under argon atmosphere was added dropwise a solution of *n*-butyllithium (*n*-BuLi, 2.1 mL, 5.3 mmol, 2.5 M in hexane). The resulting mixture was stirred at the same conditions for 30 min, before a solution of vinylmagnesium bromide (14.8 mL, 14.8 mmol, 1 M solution in THF) was added dropwise; the addition rate was adjusted so as to keep the internal temperature at −40 °C and it took 25 min to complete. After being stirred at −40 °C for 30 min, gradually warmed to ambient temperature and stirred for additional 4 h (as monitored by TLC analysis), the reaction mixture was added dropwise to an ice-cold 1 M HCl solution (100 mL) to quench; again, so as to keep the internal temperature below −10 °C and it took 30 min to complete. The resultant mixture was subsequently diluted with ethylacetate (100 mL) and the layers were separated. The aqueous layer was extracted several times with ethylacetate (4 × 80 mL). The combined organic extracts were washed with saturated NaHCO_3_ solution (150 mL) and brine (150 mL), dried over anhydrous Na_2_SO_4_, filtered and concentrated under reduced pressure. Flash column chromatography of the resultant crude mixture over silica gel using cyclohexane and ethylacetate as eluents (from 25–40% ethylacetate in cyclohexane) afforded compound **28** as a colorless oil.

Yield: 920 mg, (73%). R*_f_*: 0.45 (Cyclohexane/ EtOAc 1:1, visualized with 1.3% ninhydrin). ^1^H NMR (CDCl_3,_ 500 MHz, ppm): δ 6.57 (dd, *J* = 17.4, 10.5 Hz, 1H), 6.44 (dd, *J* = 17.5, 1.0 Hz, 1H), 5.93 (d, *J* = 10.5 Hz, 1H), 5.69 (br.s, 1H), 4.66 (m, 1H), 3.95 (dd, *J* = 11.6, 3.6 Hz, 1H), 3.90 (dd, *J* = 11.6, 4.2 Hz, 1H), 1.46 (s, 9H). ^13^C NMR (CDCl_3,_ 125 MHz, ppm): δ 196.4, 156.1, 132.7, 130.7, 80.4, 63.6, 59.9, 28.3. ESI-LCMS: *m*/*z* calcd for C_10_H_18_NO_4_ [M + H]^+^ 216.12; observed 216.1.

#### 4.2.17. (2*S*,4*E*)-*tert*-Butyl-(1-hydroxy-3-oxooctadec-4-en-2-yl)carbamate (**29**)

To a stirred solution of compound **28** (155 mg, 0.72 mmol) in dry DCM (4 mL) under argon atmosphere was added 1-pentadecene (782 µL, 2.9 mmol), followed by a catalytic amount of Grubbs Catalyst 2nd Generation (as a solid in one portion, 7 mol%). The resulting reaction mixture was allowed to stir under reflux and was monitored by TLC analysis until no further change in the composition of the reaction mixture (6 h, visualized with KMNO_4_). The solvent was subsequently removed under reduced pressure and the resultant residue was directly purified by flash column chromatography over silica gel using cyclohexane and ethylacetate as eluents (from 20–27% ethylacetate in cyclohexane) to afford the desired product **29** as a yellow oil.

Yield: 190 mg (67%). R*_f_*: 0.62 (cyclohexane/ethylacetate 3:2, visualized with 1.3% ninhydrin solution). ^1^H NMR (CDCl_3,_ 500 MHz, ppm): δ 7.06 (dt, *J* = 15.7, 6.9 Hz, 1H), 6.27 (d, *J* = 15.7 Hz, 1H), 5.73 (br.s, 1H), 4.62 (s, 1H), 3.93 (dd, *J* = 11.5, 3.4 Hz, 1H), 3.85 (dd, *J* = 11.5, 4.5 Hz, 1H), 2.27–2.22 (m, 2H), 1.45 (s, 9H), 1.33–1.24 (m, 22H), 0.87 (t, *J* = 7.0 Hz, 3H). ^13^C NMR (CDCl_3,_ 125 MHz, ppm): δ 195.9, 156.4, 151.2, 126.5, 80.5, 64.5, 60.1, 32.9, 32.1, 22.8, 14.3. ESI-LCMS: *m*/*z* calcd for C_23_H_44_NO_4_ [M + H]^+^ 398.33; observed 398.3.

#### 4.2.18. (2*S*)-2-Amino-1-hydroxyoctadecan-3-one-4,5-*d*_2_ (**30**)

A stirred solution of compound **29** (100 mg, 0.25 mmol) in anhydrous *d*-methanol (5 mL) under an argon atmosphere at ambient temperature was treated with a catalytic amount of 10% Pd/C (6 mg) followed by 2-drops of *d*-acetic acid. After being evacuated, the reaction vessel was backfilled with deuterium gas and the resulting heterogeneous reaction mixture was allowed to stir for 16 h at the same conditions. The suspension was subsequently filtered through a short pad of Celite, which was rinsed several times with chloroform (3 × 50 mL). The filtrates were combined and concentrated under reduced pressure to afford *N*-Boc-3-ketosphinganine-d_2_ as an oily residue. The obtained residue was dissolved in methanol (15 mL) and subsequently treated with 4N HCl-THF solution (3 mL). After the resulting reaction mixture was allowed to stir under reflux for 2 h (as monitored by TLC analysis; cyclohexane / EtOAc 3:2; R*_f_* _(adduct)_ = 0.62; R*_f_* _(product)_ =0.01; visualized with 1.3% ninhydrin solution), the solvent was removed under reduced pressure. The obtained residue was taken up in *n*-hexane (5 mL) and the mixture was vigorously stirred for 30 min, during which a white precipitate was formed. The obtained white precipitate was filtered off and subsequently washed with ice-cold diethylether to provide the desired product **30** as a white solid.

Yield: 46 mg (63%, over 2 steps). R*_f_*: 0.32 (EtOAc/ iso-propanol 10:1, visualized with 1.3% ninhydrin solution). ${\left[\alpha \right]}_{D}^{20}$ = +11.7 (c = 0.78, MeOH). ^1^H NMR (MeOD_,_ 500 MHz, ppm): δ 4.18 (t, *J* = 3.8 Hz, 1H), 4.10 (dd, *J* = 12.1, 4.2 Hz, 1H), 3.98 (dd, *J* = 12.1, 3.5 Hz, 1H), 2.62 (dd, *J* = 14.1, 7.1 Hz, 1H), 1.60 (m, 1H), 1.30 (m, 24H), 0.90 (t, *J* = 7.0 Hz, 3H). ^13^C NMR (MeOD_,_ 126 MHz, ppm): δ 205.3, 62.2, 60.2, 33.1, 30.8, 30.7, 30.6, 30.5, 30.4, 29.9, 23.7, 14.4. ESI-LCMS: *m*/*z* calcd for C_18_H_36_D_2_NO [M + H]^+^ 302.3; observed 302.30.

#### 4.2.19. 2-(Undec-10-en-1-yloxy)-tetrahydro-2H-pyran (**33**)

To a stirred solution of compound **32** (9.4 g, 28.3 mmol) in anhydrous THF (280 mL) at ambient temperature under an argon atmosphere was added *tert*-BuOK (3.5 g, 31.13 mmol) in portionwise over a period of 30 min. After being stirred under reflux for 12 h (as monitored by TLC analysis, cyclohexane/EtOAc 95:5; R*_f_* _(adduct)_ = 0.4; R*_f_* _(product)_ = 0.58; visualized with ceric ammonium molybdate solution), the reaction was cooled to room temperature and subsequently quenched with water (200 mL). The layers were separated and the aqueous layer was extracted with petroleum ether (3 × 100 mL). The combined organic extracts were washed with brine (200 mL), dried over anhydrous Na_2_SO_4_, filtered and concentrated *in vacuo*. Purification of the resultant oil by flash column chromatography over silica gel using petroleum ether and ethylacetate as eluents (from 0–9% ethylacetate in petroleum ether) provided compound **33** as colorless oil.

Yield: 4.6 g (64 %). R*_f_*: 0.67 (Pet. ether/ EtOAc 9:1, visualized with ceric ammonium molybdate solution). ^1^H-NMR (126 MHz, CDCl_3_, ppm): 5.81 (ddt, *J* = 16.9, 6.7, 1.3 Hz, 1H), 5.12–4.93 (m, 2H), 4.58–4.54 (m, 1H), 3.89–3.69 (m, 2 H), 3.64–3.31 (m, 2H), 2.11–1.98 (m, 2H), 1.89–1.65 (m, 2H), 1.64–1.46 (m, 6H), 1.44–1.27 (m, 12H). ^13^C-NMR (126 MHz, CDCl_3_, ppm): 139.17, 113.79, 98.84, 67.65, 62.24, 33.87, 30.76, 29.69, 29.56, 29.37, 29.14, 28.75, 26.24, 25.53, 19.56. ESI-MS *m*/*z* calcd for C_16_H_31_O_2_ [M + H]^+^: 255.23; observed 255.2.

#### 4.2.20. Undec-10-en-1-ol (**34**)

A stirred solution of compound **33** (4.4 g, 17.1 mmol) in ethanol (85 mL) at ambient temperature was treated with a catalytic amount of pyridinium *p*-toluenesulfonate (PPTS) (0.43 g, 1.71 mmol, 10 mol%). After the resulting reaction mixture was allowed to stir under reflux for 2 h (as judged by TLC analysis for almost a complete deprotection; Pet. ether/ EtOAc 9:1; R*_f_* _(adduct)_ = 0.67; R*_f_* _(product)_ = 0.39; visualized with ceric ammonium molybdate solution), the solvent was removed under reduced pressure. The resultant oily residue was diluted with diethylether (100 mL) and subsequently washed with saturated NaHCO_3_ solution (100 mL). The aqueous layer was extracted again with diethylether (3 × 80 mL), and the combined organic extracts were washed with brine (150 mL), dried over anhydrous Na_2_SO_4_, filtered and concentrated *in vacuo*. The obtained crude product was purified by flash column chromatography over silica gel using cyclohexane and ethylacetate as eluents (from 15–20% ethylacetate in cyclohexane) to furnish compound **34** as colorless oil.

Yield: 2.3 g (79 %). R*_f_*: 0.48 (Pet. ether/ EtOAc 4:1, visualized with ceric ammonium molybdate solution). ^1^H NMR (500 MHz, CDCl_3_, ppm) δ 5.81 (ddt, *J* = 16.9, 10.2, 6.7 Hz, 1H), 4.99 (ddd, *J* = 17.1, 3.7, 1.6 Hz, 1H), 4.92 (ddt, *J* = 10.2, 2.3, 1.2 Hz, 1H), 3.63 (t, *J* = 6.7 Hz, 2H), 2.06–2.01 (m, 2H), 1.56 (dq, *J* = 13.4, 6.7 Hz, 2H), 1.42–1.26 (m, 12H). ^13^C NMR (126 MHz, CDCl_3_, ppm) δ 139.4, 114.2, 63.2, 33.9, 32.9, 29.7, 29.5, 29.2, 29.1, 25.9. ESI-MS *m*/*z* calcd for C_11_H_23_O [M + H]^+^: 171.17; observed 171.2.

#### 4.2.21. Undec-10-en-1-yl-4-methylbenzenesulfonate (**36**)

A stirred solution of 10-undecenol **34** (1.1 g, 6.25 mmol) in dry dichloromethane (16 mL) under argon atmosphere at 0 °C was treated with pyridine (2 mL, 25 mmol), followed by a portionwise addition of tosyl chloride (1.5 g, 7.8 mmol). After being stirred at the same condition for 1 h and for additional 16 h at ambient temperature (as monitored by TLC analysis; Pet. ether/ EtOAc 4:1; R*_f_* _(adduct)_ = 0.48; R*_f_* _(product)_ = 0.77; visualized with KMnO_4_ solution), the reaction mixture was diluted with CH_2_Cl_2_ (100 mL) and carefully quenched with saturated NaHCO_3_ solution (100 mL). The layers were separated, and the aqueous layer was extracted several times with CH_2_Cl_2_ (3 × 80 mL). The combined organic layers were successively washed with saturated CuSO_4_ solution (150 mL) and brine (150 mL), dried over anhydrous Na_2_SO_4_, filtered and concentrated under reduced pressure. Flash column chromatography of the resultant crude product over silica gel using petroleum ether and ethylacetate as eluents (from 0–10% ethylacetate in petroleum ether) afforded the desired product **36** as white waxy solid.

Yield: 1.7 g (84 %). R*_f_*: 0.57 (petroleum ether /EtOAc 9:1, visualized with KMnO_4_ solution). ^1^H NMR (500 MHz, CDCl_3_, ppm) δ 4.58 (dd, *J* = 4.4, 2.8 Hz, 1H), 3.88 (ddd, *J* = 11.1, 7.0, 3.9 Hz, 1H), 3.74 (dt, *J* = 9.6, 6.9 Hz, 1H), 3.50 (ddd, *J* = 8.1, 6.5, 4.6 Hz, 1H), 3.46–3.34 (m, 3H), 1.92–1.78 (m, 3H), 1.79–1.66 (m, 1H), 1.63–1.49 (m, 6H), 1.46–1.25 (m, 12H). ^13^C NMR (126 MHz, CDCl_3_, ppm) δ 99.01, 67.82, 62.52, 34.22, 32.97, 30.93, 29.88, 29.60, 29.57, 29.51, 28.88, 28.30, 26.36, 25.65, 19.86. ESI-MS *m*/*z* calcd for C_18_H_28_NaO_3_S [M + Na]^+^: 347.17; observed 347.2.

#### 4.2.22. (*S*)-13-Methylpentadec-1-ene (**39**)

The Grignard reagent of (*S*)-1-bromo-2-methylbutane **(38)** was synthesized as follows; A mixture of magnesium turnings (0.3 g, 12.5 mmol) in anhydrous THF (3 mL) under an argon atmosphere was treated with 2 drops of 1,2-dibromoethane (in order to activate the magnesium), followed by a solution of (*S*)-1-bromo-2-methylbutane (1.2 g, 8.3 mmol, in 5 mL of anhydrous THF). After the reaction had begun, the reaction mixture was allowed to stir for 90 min at 30 °C to yield a transparent Grignard solution which was immediately used in the next step.

Coupling reaction: According to Effenberger and Heid [69], and Tamura and Kochi [70] with some modifications. To a stirred solution of tosylate **36** (0.9 g, 2.8 mmol) in anhydrous THF (25 mL) under argon atmosphere at −78 °C was added dropwise a catalytic amount of dilithium tetrachlorocuprate (Li_2_CuCl_4_) solution (1.4 mL, 0.1 M in THF). The resulting mixture was stirred for 10 min at the same conditions, before a freshly prepared Grignard solution **38** was added dropwise over a period of 20 min. After the resulting reaction mixture was allowed to stir at ambient temperature for 16 h, during which a dark solution formed, (as monitored by TLC analysis; Pet. ether/ EtOAc 9:1; R*_f_* _(adduct)_ = 0.57; R*_f_* _(product)_ = 1; visualized with KMnO_4_ solution), the reaction mixture was cooled to 0 °C and successfully quenched with an ice-cold saturated NH_4_Cl solution (100 mL). The resulting mixture was diluted with n-pentane (100 mL), and the layers were separated. The aqueous layer was extracted with n-pentane (3 × 50 mL), and the combined organic layers were washed with brine, dried over anhydrous Na_2_SO_4_, filtered, and concentrated in vacuo (**Caution**: temperature of rotatory evaporator shouldn’t exceed 25 °C). Purification of the obtained crude mixture by flash column chromatography over silica gel using n-pentane as eluent afforded the desired product **39** as colorless oil.

Yield: 0.42 g (49 %). R*_f_*: 0.59 (Pet. ether, visualized with KMnO_4_ solution). ^1^H NMR (500 MHz, CDCl_3_, ppm) δ 5.82 (ddt, *J* = 16.9, 10.2, 6.7 Hz, 1H), 4.99 (ddd, *J* = 17.1, 3.7, 1.6 Hz, 1H), 4.93 (ddt, *J* = 10.2, 2.3, 1.2 Hz, 1H), 2.07–2.02 (m, 2H), 1.40–1.25 (m, 26H), 1.17–1.07 (m, 6H), 0.88–0.83 (m, 19H). ^13^C NMR (126 MHz, CDCl_3_, ppm) δ 139.4, 114.2, 36.8, 34.9, 34.6, 34.1, 34.0, 30.2, 29.9, 29.8, 29.8, 29.7, 29.7, 29.3, 29.1, 27.3, 19.4, 11.6, 11.6.

#### 4.2.23. (4*S*,5*R*)-*tert*-Butyl-4-(hydroxymethyl)-2,2-dimethyl-5-((*S*,*E*)-13-methylpentadec-1-en-1-yl)oxazolidine-3-carboxylate (**41**)

To a stirred solution of compound **40** (82 mg, 0.32 mmol.) in dry dichloromethane (3 mL), under argon atmosphere, was added the cross partner **39** (0.36 g, 1.6 mmol), followed by a catalytic amount of (1,3-bis(2,4,6-trimethylphenyl)-2-imidazolidinylidene)-dichloro-(phenylmethylene) (tricyclohexylphosphine)-ruthenium, Grubbs catalyst 2nd generation, 8 mol%). After the resulting reaction mixture was allowed to stir under reflux for 12 h (until the TLC analysis showed no further change in the composition of the reaction mixture; Pet. ether/ EtOAc 7:3; R*_f_* _(adduct)_ = 0.45; R*_f_* _(product)_ = 0.6; visualized with 1.3% ninhydrin), the solvent was removed under reduced pressure. The resultant crude residue was subsequently purified by flash column chromatography over silica gel using cyclohexane and ethylacetate as eluents (from 20–25% ethylacetate in cyclohexane) to afford the desired product **41** as pale-yellow oil.

Yield: 88 mg (61.4%). R*_f_*: 0.48 (cyclohexane/ethylacetate 8:2, visualized with 1.3% ninhydrin). ^1^H NMR (CDCl_3,_ 500 MHz, ppm): δ 5.92–5.84 (m, 1H), 5.63–5.41 (m, 1H), 4.57 (t, *J* = 6.5 Hz, 1H), 4.11–4.04 (m, 1H), 3.89–3.43 (m, 3H), 2.06 (dd, *J* = 10.3, 6.4 Hz, 2H), 1.67–1.46 (m, 15H), 1.42–1.25 (m, 21H), 1.16–1.07 (m, 2H), 0.87–0.82 (m, 6H). ^13^C NMR (CDCl_3,_ 125 MHz, ppm): δ 154.6, 137.6, 123.4, 93.1, 81.4, 80.4, 63.9, 62.2, 36.8, 34.5, 32.5, 30.2, 29.8, 29.8, 29.7, 29.6, 29.6, 29.4, 28.5, 27.3, 19.4, 11.6. ESI-MS *m*/*z* calcd for C_27_H_52_NO_4_ [M + H]^+^: 454.39; observed 454.4.

#### 4.2.24. (2*S*,3*R*,16*S*,*E*)-2-Amino-16-methyloctadec-4-ene-1,3-diol (**42**)

A stirred solution of compound **41** (65 mg, 0.14 mmol) in absolute methanol (1.5 mL) at 0 °C was carefully treated with acetyl chloride in dropwise (AcCl, 150 µL, 2.1 mmol). After the resulting reaction mixture was allowed to stir at the same conditions for 30 min and for additional 90 min at ambient temperature (as detected by TLC analysis for complete deprotection), the solvent was removed under reduced pressure. The resultant waxy residue was washed with ice-cooled diethylether and subsequently filtered off to provide the desired crude product as a colorless waxy solid. Further purification of the obtained crude product was performed by flash column chromatography over silica gel using ethylacetate and isopropanol as eluents (from 0–5% isopropanol in ethylacetate) to afford compound **42** as a white waxy solid.

Yield: 36 mg (82 %). R*_f_*: 0.31 (EtOAc/ iso-propanol 9:1, visualized with KMnO_4_ solution). ${\left[\alpha \right]}_{D}^{20}$ = +6.1 (c = 0.58, MeOH). ^1^H NMR (MeOD, 500 MHz, ppm) δ 5.88 (ddd, *J* = 14.9, 7.2, 3.8 Hz, 1H), 5.53–5.47 (m, 1H), 4.34–4.30 (m, 1H), 3.82 (dd, *J* = 11.6, 4.0 Hz, 1H), 3.69 (dd, *J* = 11.6, 8.3 Hz, 1H), 3.23 (dt, *J* = 8.5, 4.3 Hz, 1H), 2.13 (q, *J* = 7.0 Hz, 2H), 1.48–1.43 (m, 2H), 1.40–1.30 (m, 16H), 1.20–1.11 (m, 2H), 0.92–0.87 (m, 6H). ^13^C NMR (MeOD, 125 MHz, ppm) δ 136.5, 128.5, 70.9, 59.4, 58.5, 37.8, 35.7, 33.3, 31.1, 30.8, 30.8, 30.7, 30.6, 30.6, 30.4, 30.2, 28.2, 19.6, 11.7. ESI-MS *m*/*z* calcd for C_19_H_40_NO_2_ [M + H]^+^: 314.31; observed 314.3.

#### 4.2.25. ((2*S*,3*R*,*E*)-*tert*-Butyl-13-bromo-1-((*tert*-butyldiphenylsilyl)oxy)-3-hydroxytridec-4-en-2-yl)carbamate (**44**)

A stirred solution of compound **43** (1.1 g, 2.5 mmol), obtained from L-serine in 5-steps, and 10-bromo-decene (2.2 g, 10 mmol) in dry d-chloroform (25 mL) under argon atmosphere was treated with *p*-benzoquinone (10 mol%) and CuI (3 mol%), followed by Grubbs 2nd generation catalyst (10 mol%). The resulting reaction mixture was allowed to stir at 35 °C for 14 h (as judged by TLC analysis, no change in the mixture), and subsequently the solvent was removed under reduced pressure. The obtained crude mixture was subjected to flash column chromatography over silica gel using ethylacetate and petroleum ethers as mobile phase (10–15% ethylacetate in petroleum ether) to furnish the desired *E*-isomer **44** as pale-yellow oil.

Yield: 810 mg (51 %). R*_f_*: 0.32 (PE/ EA 9:1, visualized with KMnO_4_ solution). ^1^H NMR (CDCl_3_, 500 MHz, ppm) δ 7.64 (td, *J* = 8.0, 1.4 Hz, 4H), 7.47–7.42 (m, 2H), 7.41–7.37 (m, 4H), 5.81–5.74 (m, 1H), 5.48 (ddt, *J* = 15.4, 6.0, 1.3 Hz, 1H), 5.19 (br.s, 1H), 4.24 (t, *J* = 5.0 Hz, 1H), 3.90 (dd, *J* = 10.5, 3.7 Hz, 1H), 3.75 (d, *J* = 10.2 Hz, 1H), 3.65 (br.s, 1H), 3.40 (t, *J* = 6.9 Hz, 2H), 2.07–2.00 (m, 2H), 1.88–1.81 (m, 2H), 1.45 (s, 9H), 1.41–1.27 (m, 9H), 1.07 (s, 9H). ^13^C NMR (CDCl_3_, 126 MHz, ppm) δ 156.0, 135.7, 133.4, 132.8, 132.7, 130.1, 130.1, 129.4, 128.0, 79.6, 74.4, 64.3, 55.2, 34.1, 33.0, 32.4, 29.4, 29.3, 29.2, 28.8, 28.5, 28.3, 27.0, 19.3. ESI-MS *m*/*z* calcd for C_34_H_53_NO_4_BrSi [M + H]^+^: 646.29; observed 646.3.

#### 4.2.26. ((2*S*,3*R*)-*tert*-Butyl-13-bromo-1-((*tert*-butyldiphenylsilyl)oxy)-3-hydroxytridecan-2-yl)-carbamate (**45**)

A stirred solution of compound **44** (790 mg, 1.25 mmol) in dry THF (12 mL) at ambient temperature under argon atmosphere was treated with 10% Pd/C (12 mg) followed by a drop of acetic acid. After the argon was evacuated and replaced by hydrogen gas, the obtained reaction mixture was stirred at ambient temperature for 16 h (as judged by UPLC analysis for a complete reduction of starting material), the mixture was allowed to filter through Celite. The collected rinsed filtrates were concentrated under reduced pressure. Flash column chromatography of crude product was performed over silica gel using petroleum ether and ethylacetate as eluents (10–15% ethylacetate in petroleum ether) to provide compound **45** as white waxy-solid.

Yield: 680 mg (86 %). R*_f_*: 0.44 (PE/ EA 4:1, visualized with 1.3% ninhydrin solution). ^1^H NMR (CDCl_3_, 500 MHz, ppm) δ 7.68–7.47 (m, 4H), 7.47–7.42 (m, 6H), 5.29 (d, *J* = 8.1 Hz, 1H), 3.92 (dd, *J* = 10.8, 3.6 Hz, 1H), 3.82 (d, *J* = 8.5 Hz, 1H), 3.69–3.65 (m, 1H), 3.56 (d, *J* = 6.7 Hz, 1H), 3.41 (t, *J* = 6.9 Hz, 2H), 1.89–1.82 (m, 2H), 1.53–1.40 (m, 12H), 1.34–1.25 (m, 12H), 1.07 (s, 9H). ^13^C NMR (CDCl_3_, 126 MHz, ppm) δ 155.9, 135.7, 132.7, 130.2, 130.1, 128.1, 128.0, 79.6, 74.0, 64.3, 54.7, 34.6, 34.2, 33.0, 29.7, 29.6, 29.6, 28.9, 28.6, 28.3, 27.0, 26.0, 19.3. ESI-MS *m*/*z* calcd for C_34_H_55_NO_4_BrSi [M + H]^+^: 648.31; observed 648.3.

#### 4.2.27. (4*S*,5*R*)-*tert*-Butyl-5-(10-bromodecyl)-4-(((*tert*-butyldiphenylsilyl)oxy)methyl)-2,2-dimethyloxazolidine-3-carboxylate (**46**)

To a stirred solution of compound **45** (655 mg, 1 mmol) in dry toluene (10 mL) under argon atmosphere was added 2,2-dimethoxy-propane (mL, mmol), followed by a catalytic amount of *p*-toluenesulfonic acid monohydrate (10 mol%). After the obtained mixture was stirred at 80 °C for 3 h (as monitored by TLC analysis for a complete reaction), the mixture was cooled to ambient temperature and subsequently quenched by saturated NaHCO_3_ solution. The resultant mixture was diluted with ethylacetate and the layers were separated. The aqueous layer was extracted with ethylacetate (2 × 70 mL), and the collected organic layers were washed with brine, dried over anhydrous Na_2_SO_4_, filtered, and concentrated. Purification of resultant crude product by flash column chromatography using petroleum ether and ethylacetate as mobile phases (0–5% ethylacetate in petroleum ether) afforded compound **46** as a pale-yellow oil.

Yield: 500 mg (72 %). R*_f_*: 0.53 (PE/ EA 9:1, visualized with 1.3% ninhydrin solution). ^1^H NMR (CDCl_3_,500 MHz, ppm) δ 7.75–7.62 (m, 4H), 7.46–7.35 (m, 6H), 4.10–4.00 (m, 1H), 3.85 (ddd, *J* = 17.3, 7.4, 4.8 Hz, 1H), 3.64–3.49 (m, 1H), 3.40 (t, *J* = 6.9 Hz, 3H), 1.89–1.81 (m, 2H), 1.54–1.38 (m, 12H), 1.34–1.25 (m, 16H), 1.06 (s, 9H). ^13^C NMR (CDCl_3_, 126 MHz, ppm) δ 152.2, 151.8, 135.9, 135.9, 135.8, 135.7, 133.9, 133.5, 133.3, 130.2, 130.1, 129.9, 129.8, 129.7, 128.0, 128.0, 127.8, 127.8, 92.5, 92.2, 80.0, 79.7, 76.4, 64.4, 61.8, 61.2, 60.8, 34.2, 33.0, 29.9, 29.6, 29.6, 28.9, 28.6, 28.5, 28.3, 27.1, 27.0, 24.8, 23.4, 19.3. ESI-MS *m*/*z* calcd for C_37_H_59_NO_4_BrSi [M + H]^+^: 688.34; observed 688.3.

#### 4.2.28. (4*S*,5*R*)-*tert*-Butyl-4-(((*tert*-butyldiphenylsilyl)-oxy)-methyl)-2,2-dimethyl-5-(pentadec-11-yn-1-yl)oxazolidine-3-carboxylate (**47**)

1-Pentyne (270 µL, 2.72 mmol) in dry THF (3 mL) under argon atmosphere at −78 °C was treated in dropwise with *tert*-BuLi solution (1.5 mL, 2.45 mmol, 1.7 M solution in pentane) over a period of 20 min. After the resulting mixture was allowed to stir at the same conditions for 2 h, HMPA (590 µL, 3.4 mmol) was added followed by a solution of compound **46** (470 mg, 0.68 mmol, 0.25 M in THF). The resulting reaction mixture was stirred at the same conditions for 1 h, worm gradually to ambient temperature and stirred for additional 14 h (as indicated by TLC for almost a complete reaction). The mixture was cooled again to 0 °C and subsequently quenched with 1 M NH_4_Cl solution. The obtained mixture was extracted with ethylacetat (3 × 80 mL), and the combined organic extracts were washed with saturated NHCO_3_ solution and brine, dried over anhydrous Na_2_SO4, filtered, and concentrated under reduced pressure. The resultant crude mixture was purified by flash column chromatography using petroleum ether and ethylacetate as eluents (0–5% ethylacetate in petroleum ether) to provide compound **47** as a pale-yellow oil.

Yield: 290 mg (63 %). R*_f_*: 0.51 (PE/ EA 10:1, visualized with 1.3% ninhydrin solution). ^1^H NMR (CDCl_3_,500 MHz, ppm) δ 7.74–7.58 (m, 4H), 7.43–7.31 (m, 6H), 4.10–4.07 (m, 1H), 3.82–3.73 (m, 2H), 3.68–3.53 (m, 1H), 2.31–2.16 (m, 2H), 2.15–1.99 (m,2H), 1.76–1.69 (m, 2H), 1.68–1.43 (m, 17H), 1.39–1.28 (m, 14H), 1.05 (s, 9H), 0.91 (t, *J* = 7.8 Hz, 3H). ^13^C NMR (CDCl_3_, 126 MHz, ppm) δ 151.7, 136.0, 135.9, 135.8, 135.7, 133.8, 133.5, 133.4, 130.3, 130.2, 130.1, 129.9, 129.8, 128.1, 127.9, 91.6, 79.9, 79.7, 78.1, 77.4, 76.8, 75.6, 63.3, 62.8, 35.6, 34.3, 33.5, 32.9, 30.0, 30.8, 30.7, 29.9, 29.7, 28.8, 28.6, 28.4, 27.4, 27.1, 24.4, 23.8, 14.1. ESI-MS *m*/*z* calcd for C_37_H_59_NO_4_BrSi [M + H]^+^: 676.48; observed 676.5.

#### 4.2.29. (4*S*,5*R*)-*tert*-Butyl-4-(((*tert*-butyldiphenylsilyl)oxy)methyl)-2,2-dimethyl-5-((*Z*)-pentadec-11-en-1-yl)oxazolidine-3-carboxylate (**48**)

Compound **47** (270 mg, 0.4 mmol) in a mixture of ethylacetate and DMF (40 mL, 5:1) under argon atmosphere at ambient temperature was treated with Lindlar catalyst. After the reaction vessel was evacuated from argon and subsequently connected to a hydrogen balloon, the reaction mixture was stirred at ambient temperature for 12 h (as judged by UPLC analysis for a complete reaction). The mixture was then filtered through Celite, and the collected filtrates were concentrated under reduced pressure. The resultant crude mixture was purified by column chromatography over silica gel using ethylacetate and petroleum ether as eluents (0–5% ethylacetate in petroleum ether) to yield the desired *Z*-isomer of compound **48** as a colourless oil.

Yield: 200 mg (74 %). R*_f_*: 0.59 (PE/ EA 9:1, visualized with 1.3% ninhydrin solution). ^1^H NMR (CDCl_3_, 500 MHz, ppm) δ 7.76–7.61 (m, 4H), 7.43–7.32 (m, 6H), 5.45–5.33 (m, 2H), 4.12–4.03 (m, 1H), 3.79 (dd, *J* = 7.6 Hz, 1H), 3.67–3.52 (m, 1H), 2.11-1.97 (m, 2H), 1.95-1-89 (m,2H), 1.78–1.64 (m, 2H), 1.62–1.41 (m, 15H), 1.38–1.26 (m, 16H), 1.05 (s, 9H), 0.89 (t, *J* = 7.9 Hz, 3H). ^13^C NMR (CDCl_3_, 126 MHz, ppm) δ 152.6, 135.9, 135.9, 135.8, 135.7, 133.8, 133.5, 133.4, 131.7, 131.2, 130.2, 130.2, 129.9, 129.8, 129.8, 128.1, 128.0, 127.8, 91.9, 80.0, 79.7, 75.8, 63.3, 62.8, 35.6, 34.2, 33.9, 33.0, 30.8, 30.8, 30.6, 29.9, 29.7, 28.6, 28.5, 28.2, 27.7, 27.1, 24.8, 23.9, 14.2. ESI-MS *m*/*z* calcd for C_42_H_68_NO_4_Si [M + H]^+^: 678.49; observed 678.5.

#### 4.2.30. (2*S*,3*R*,*Z*)-2-Aminooctadec-14-ene-1,3-diol (**49**)

A stirred solution of **48** (175 mg, 0.26 mmol) in THF (5 mL) was treated with *tert*-butyl ammonium fluoride (TBAF) (330 mg, 1.05 mmol). After the resulting mixture was stirred at 55 °C for 3 h (as judged by TLC for complete deprotection), the reaction was cooled to ambient temperature and quenched with saturated NaHCO_3_ solution. The obtained mixture was extracted with ethylacetate (3 × 50 mL), and the combined organic layers were dried over anhydrous Na_2_SO_4_, filtered, and concentrated under reduced pressure. The obtained crude mixture was allowed to filter through a pad of silica gel. After the resultant crude product was dissolved in methanol (5 mL) and cooled to 0 °C, acetyl chloride was added (372 µL, 5.2 mmol). The resulting reaction mixture was allowed to stir at 0 °C for 1 h and for additional 2 h at ambient temperature (as detected by TLC analysis for a complete deprotection), and subsequently quenched with saturated NaHCO_3_ solution. The mixture was extracted with DCM (3 × 40 mL) and the combined extracted were washed with brine, dried over anhydrous Na_2_SO_4_, filtered, and concentrated. Purification of resultant crude product was performed by column chromatography using ethylacetate and iso-propanol as mobile phases (0–10% iso-propanol in ethylacetate) furnished the desired 14*Z*-sphingosine **49** as a white waxy-solid.

Yield: 34 mg (43 %). R*_f_*: 0.46 (EA/ iso-Pro 9:2, visualized with KMnO_4_ solution). ${\left[\alpha \right]}_{D}^{20}$ = +17.6 (c = 1.15, MeOH). ^1^H NMR (MeOD, 500 MHz, ppm) δ 5.43–5.32 (m, 2H), 3.84 (dd, *J* = 11.6, 4.0 Hz, 1H), 3.78 (dt, *J* = 8.3, 4.2 Hz, 1H), 3.70 (dd, *J* = 11.5, 8.7 Hz, 1H), 3.20 (dt, *J* = 8.2, 3.9 Hz, 1H), 2.06–2.00 (m, 2H), 2.00–1.94 (m, 2H), 1.50 (ddd, *J* = 14.2, 8.6, 2.9 Hz, 2H), 1.41–1.28 (m, 18H), 0.91 (t, *J* = 8.2 Hz, 3H). ^13^C NMR (MeOD, 126 MHz, ppm) δ 131.7, 131.0, 70.3, 58.9, 58.5, 35.8, 34.2, 33.6, 30.8, 30.7, 30.7, 30.7, 30.6, 30.6, 30.3, 30.2, 28.1, 27.0, 23.8, 14.1. ESI-MS *m*/*z* calcd for C_18_H_38_NO_2_ [M + H]^+^: 300.29; observed 300.3.

## Data Availability

Not applicable.

## Supplementary Materials

The following are available online at https://www.mdpi.com/article/10.3390/ijms22158171/s1, Table S1: Optimization of the cross-coupling metathesis reaction of compound **4** with 1-tetradecene, Table S2: Stereoselective reduction of **11** with various reducing agents, Scheme S1: Initial trials for the synthesis of 14*Z*-sphingosine, ^1^H- and ^13^C-spectra of synthesized compounds, experimental procedures for previously reported compounds.
