# Supplementary material for: Stereoselective Synthesis of Novel Sphingoid Bases Utilized for Exploring the Secrets of Sphinx
Source: Int J Mol Sci. 2021 Jul 29;22(15):8171. doi: 10.3390/ijms22158171 (PMC8347175; doi:10.3390/ijms22158171)
Supplement: Supplementary file 1 [file ijms-22-08171-s001.zip › ijms-1263645-supplementary.pdf]

Supporting Information

Stereoselective Synthesis of Novel Sphingoid Bases Utilized for Exploring the Secrets of Sphinx

Essa M. Saied [1,2] and Christoph Arenz [1,*]

1 Institute for Chemistry, Humboldt Universität zu Berlin, Brook-Taylor-Str. 2, 12489 Berlin, Germany; saiedess@hu-berlin.de
2 Chemistry Department, Faculty of Science, Suez Canal University, Ismailia 41522, Egypt
* Correspondence: arenzchr@hu-berlin.de

Table S1: Optimization of the cross-coupling metathesis reaction of compound **4** with 1-tetradecene

Entry	1-tetradecene (equiv.)	Catalyst (10mol%)	Additive	Reaction conditions	Yield	Isomerization product
**1**	3	Grubbs 2nd G	-	DCM, r.t., 16h<br>or<br>DCM, reflux, 6h	71%	detected
**2**[a]	8	Grubbs 2nd G	-	DCM, r.t., 16h	66%	detected
**3**	5	H. Grubbs 2nd	-	DCM, r.t., 16h	69%	detected
**4**[b]	4	Grubbs 2nd G	acetic acid (10mol%)	Toluene, reflux	49%	detected (>5%)
**5**	4	Grubbs 2nd G	CuI (3mol%)	Et_2O, refflux	82%	detected
**6**[c]	4	Grubbs 2nd G	p-benzoquinone (10mol%)	CD_2Cl_2, r.t., 14h	32%	not detected
**7**[c]	4	Grubbs 2nd G	p-benzoquinone (10mol%)<br>CuI (3mol%)	CD_2Cl_2, r.t., 14h	46%	not detected

(a) Catalyst was added portionwise over 2h
(b) Homo-coupling of starting material was detected
(c) Significant amount of starting material was recovered
(d) Reaction was run in 0.05M

Table S2: Stereoselective reduction of **11** with various reducing agents:

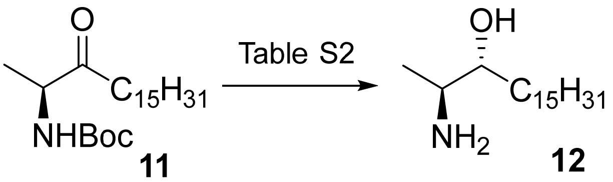

Reagent	Conditions	Yield (anti/syn)
**LAH**	THF, -20°C	89% (1:1)
**$NaBH_4$**	MeOH, -78°C-r.t	68% (1:1)
**DIBAL-H**	DCM, -78°C	81% (7:4)
**TBLA-H**	EtOH, -78°C	73% (3:1)

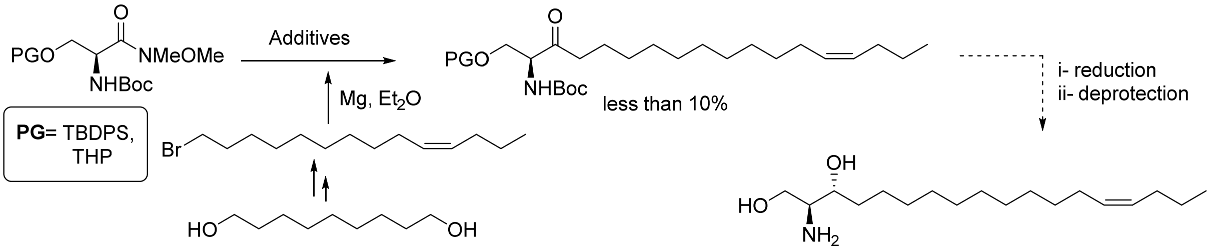

Scheme S1: Initial trials for the synthesis of 14Z-sphingosine. We have followed a synthetic route similar to that we have developed for the 1-deoxysphingosine [1]. The 11*Z*-pentadecene was obtained in 5-steps starting from 1,10-decandiol. The *O*-protected L-serine Weinreb amide was synthesized in 3-steps as previously reported [2]. Our trials only provided the desired product in less than 10%. Attempts to improve the yield by changing the O-protecting group, adding additives (e.g., LiCl), refluxing, or adding more equivalent of 11*Z*-pentadecenylmagnesium bromide, did not significantly improve the yield.

1. General description of methods

Unless otherwise specified, all reactions were carried out in oven-dried (>120°C) glassware equipped with a magnetic stir bar and a rubber septum under a positive pressure of argon. Air- or moisture-sensitive reagents were transferred to the reaction vessel under positive pressure of argon via syringe. Air and/or moisture sensitive reactions were carried out in well dried glassware under an argon atmosphere with dry, freshly distilled solvents using standard syringe-cannula/septa techniques. Reactions were run at room temperature (20-25°C) unless otherwise noted in the experimental procedure, and reported reaction temperatures refer to the external temperatures measured for the bath in which the reaction vessel was immersed. Heating was obtained through the use of a silicone oil bath. For reactions run below room temperature, the term "-78°C" refers to a bath of acetone and dry ice, "-20°C" refers to a slurry of sodium chloride and ice-water bath, and "0°C" refers to an ice-water bath. Removal of residual solvents was accomplished by evacuation of the container for a period of 12-20 hours using a high vacuum line.

Reagents and solvents

All the commercially available reagents were purchased from Sigma-Aldrich, TCI, Fluka or Acros and used without further purification, unless otherwise specified. All the solvents were used after distillation by standard methods. The petroleum ether used throughout this study had a boiling range of 40–60°C.

Chromatography

The thin layer chromatography studies were performed on pre-coated silica gel 60-F_{254} on aluminum sheets (Merck KGaA) and spots were detected by UV illumination (254 nm), and/or spraying with 1.3% ninhydrin solution, ceric ammonium molybdate (Seebach reagent) solution (25 g $MoO_3 \cdot H_3PO_4 \cdot H_2O$, 10 g $Ce(SO_4)_2 \cdot 4H_2O$, 60 ml H_2SO_4 and 905 ml of H_2O) or $KMnO_4$ solution (1.5 g $KMnO_4$, 10 g K_2CO_3 and 1.25 ml 10% NaOH in 200 ml water) followed by heating. Preparative flash column chromatography was performed manually using glass columns of different size packed with Silica Gel 60M (0.04-0.063 mm) as stationary phase with indicated eluent systems in parenthesis following the description of purification. Solvent ratios for chromatography and R_f values are reported in v/v% ratios.

Spectroscopic Data

The structure of all synthesized compounds was confirmed with ^{1}H NMR, ^{13}C NMR, DEPT, ^{31}P NMR and MS analysis. ^{1}H, ^{13}C and ^{31}P NMR spectra were recorded on Bruker AVANCE II 300, AVANCED PX 300, AVANCE 400 and Bruker ADVANCE III 500 spectrometers (^{1}H at 300, 400 or 500 MHz, ^{13}C at 75.4, 101.2 or 125.7 MHz and ^{31}P at 161.9 or 202.4 Hz) as solutions in $CDCl_3$, CD_3OD or mixtures of those at 25 °C. Chemical shifts (δ) are reported in parts per million (ppm): multiplicities are indicated as s (singlet), d (doublet), t (triplet), q (quartet), m (multiplet) and br (broad). Chemical shifts are given in ppm with respect to TMS as an external standard, (^{1}H, APT, ^{13}C, $\delta = 0.00$) with calibration against the residual solvent signal or 85% H_3PO_4 (^{31}P, $\delta = 0.00$) as external standard. The coupling constants J are given in Hz.

Mass Spectroscopy

Mass spectroscopy (MS) experiments were recorded on an AGILENT 6120 UPLC–MS system consisting of an SQD (single quadrupole detector) mass spectrometer equipped with an electrospray ionization interface (ESI) in the positive and negative ion detection modes. The samples were separated on a Zorbax Eclipse Plus C18 column (particle size 1.8 μ m, 2.1 × 50 mm) using a UPLC pump at a flow rate of 0.8 ml per min with a ternary solvent system of MeOH-H_2O-HCOOH, methanol (99.9%

MeOH: 0.1% HCOOH, v/v). The column was first equilibrated using a mixture of 95% mobile phase A and 5% mobile phase B, and then 10 μ l of the sample was injected. This was followed by a ramp gradient over 2 min to 95% phase B and 5% phase A, which remained until 7 min, followed by a ramp gradient back down to 95% solvent A and 5% solvent B for 1 min, and column equilibration with the same mixture for 1 min. The detection was performed in full scan mode and the major observable molecular ion and selected fragments and clusters have been reported.

2. Synthetic procedures and analytical data.

Synthesis of (*tert*-butoxycarbonyl)-L-alanine (1)

A solution of di-*tert*-butyl dicarbonate (14.34 g, 70.72 mmol) in dioxane (120 mL) was added dropwise to a stirred solution of L-alanine (6.00 g, 67.34 mmol) in aqueous 1M NaOH (120 mL) at -10ºC. After the resulting reaction mixture was stirred for 12h at ambient temperature, the reaction mixture was washed with ethylacetate (2×150 mL) to remove excess of unreacted Boc_2O. The aqueous layer was cooled again at -15ºC and carefully acidified with a solution of 1M $KHSO_4$ to pH=2-3. The resulting mixture was subsequently extracted several times with ethylacetate (4×150 mL). The organic layers were combined, dried over anhydrous Na_2SO_4, filtered and concentrated *in vacuo* to provide *N*-Boc-L-alanine as white solid. The product was used in the next step without further purification.

Yield: 11.4 g (87%). R_f: 0.3 ($CHCl_3$/MeOH 12:1; visualized with 1.3% ninhydrine solution). ^{1}H NMR ($CDCl_3$, 500 MHz, ppm): δ 5.12-5.04 (m, 1H), 4.40-4.32 (m, 1H), 1.48-1.43 (m, 12H). ^{13}C NMR ($CDCl_3$, 126 MHz, ppm): δ 177.8, 155.6, 80.5, 49.2, 28.4, 18.4. ESI-LCMS: *m/z* calcd for $C_8H_{16}NO_4$ $[M+H]^+$ 190.11; observed 190.1. [1,4]

Synthesis of (*S*)-*tert*-butyl-1-(*N*-methoxy-*N*-methylcarbamoyl)ethylcarbamate (2)

To a stirred solution of *N*-Boc-L-alanine (4.50 g, 23.8 mmol), *N,O*-dimethylhydroxylamine hydrochloride (2.6 g, 26.2 mmol) and *N*-methylmorpholine (5.2 mL, 47.6 mmol) in dry DCM (100 mL) at -10°C under an argon atmosphere, 1-ethyl-3-(3-dimethylaminopropyl)carbodiimide hydrochloride (5 g, 26.2 mmol) was added portionwise over a period of 30 min. The resulting reaction mixture was allowed to stir for 4h at 0°C (as monitored by TLC analysis for almost complete reaction; Silica gel, EtOAc 100%; $R_{f(adduct)}$= 0.1; $R_{f(product)}$=0.56; visualized with 1.3% ninhydrine solution), and was then quenched with saturated NH_4Cl solution (200 mL). The layers were separated, and the aqueous layer was extracted several times with CH_2Cl_2 (4×100 mL). The organic layers were combined, washed with saturated $NaHCO_3$ solution (120 mL), brine (120 mL), dried over anhydrous Na_2SO_4, filtered and concentrated under reduced pressure to afford compound **2** as a white solid. The product was used in the next step without any further purification.

Yield: 4.1 g (76 %). R_f: 0.42 (cyclohexane/ethylacetate 3:2, visualized with 1.3% ninhydrine). ^{1}H NMR ($CDCl_3$, 500 MHz, ppm): δ 5.27 (d, *J*= 6.9 Hz, 1H), 4.74-4.62 (br. m, 1H), 3.77 (s, 3H), 3.21 (s, 3H), 1.44 (s, 9H), 1.31 (d, *J*= 6.9 Hz, 3H). ^{13}C NMR ($CDCl_3$, 126 MHz, ppm): δ 18.8, 28.5, 32.3, 46.7, 61.7, 79.6, 155.3, 173.8. ESI-LCMS: *m/z* calcd for $C_{10}H_{21}N_2O_4$ $[M+H]^+$ 233.15; observed 233.1. [1,4]

Synthesis of (*S*)-*tert*-butyl -(3-oxohex-5-en-2-yl)carbamate (3)

The 1-allylmagnesium bromide was prepared as follows; Catalytic drops of 1,2-dibromoethane was added to a mixture of magnesium powder (1.84 g, 77 mmol) in anhydrous Et_2O (10mL) under an argon atmosphere at ambient temperature. The resulting mixture was stirred at the same conditions for 10 min, before it treated with a solution of 1-allylbromide (2.4 g, 19.3 mmol, 1M solution in anhydrous Et_2O). The resulting reaction mixture was allowed to stir at 35°C for 3h to afford a transparent allylmagnesium bromide solution which was used in the next step.

A freshly prepared allylmagnesium bromide solution (19.3 mmol, 20 mL) was added dropwise to a stirred solution of Weinreb amide **2** (1.3 g, 5.5 mmol) in dry diethylether (mL) under argon atmosphere at 0°C. After being stirred at the same conditions for 30 min and for additional 2h at ambient temperature (as judged by TLC analysis; Pet. ether/ EtOAc 4:1; $R_{f\ (adduct)}$= 0.2; $R_{f\ (product)}$=0.54;visualized with 1.3% ninhydrine), the reaction mixture was dropwisely added to an ice-cooled 1M HCl solution (100 mL) to quench. The resulting mixture was diluted with ethylactetate and transferred to separating funnel. The layers were separated and the aqueous layer was extracted again with ethylacetate (2x100 mL). The organic extracts were combined, washed with sat $NaHCO_3$ solution and brine solution, dried over anhydrous Na_2SO_4, filtered and concentrated *in vacuo* to provide a brownish oil residue. Purification of the resultant crude residue with flash column chromatography over silica gel using petroleum ether and ethylacetate as eluents (0-10% ethylacetate in petroleum ether) afforded the product **3** as a white solid.

Yield: 710 mg (61 %). R_f: 0.54 (Pet. ether /EtOAc 4:1, visualized with 1.3% ninhydrine solution). ^{1}H NMR ($CDCl_3$, 500 MHz, ppm) δ 5.91 (ddt, *J* = 17.1, 10.2, 6.9 Hz, 1H), 5.20 (ddd, *J* = 10.2, 2.7, 1.3 Hz, 1H), 5.16 (dq, *J* = 17.1, 1.5 Hz, 1H), 4.39 – 4.32 (m, 1H), 3.33 – 3.22 (m, 2H), 1.43 (s, 9H), 1.33 (d, *J* = 7.2 Hz, 3H). ^{13}C NMR ($CDCl_3$, 126 MHz, ppm) δ 207.5, 155.3, 129.9, 119.4, 79.9, 54.9, 44.1, 28.5, 17.8. ESI-MS *m/z* calcd for $C_{11}H_{20}NO_3$ $[M+H]^+$ 214.14; observed: 214.1. [5]

Synthesis of (2*S*,3*R*)-*tert*-butyl (3-hydroxyhex-5-en-2-yl)carbamate (4)

Lithium tri-(*tert*-butoxy)-aluminum hydride (1.7 g, 6.75 mmol) was added in portionwise to a stirred solution of compound **3** (0.58 g, 2.7 mmol) in dry ethanol (6 mL) under argon atmosphere at -78°C. The resulting reaction mixture was allowed to stir at the same conditions for 2h (as judged by TLC analysis; Pet. ether/ EtOAc 4:1; $R_{f\ (adduct)}$= 0.54; $R_{f\ (product)}$=0.42; visualized with 1.3% ninhydrine), before it was quenched with ice-cooled 1M HCl solution (120 mL). The resulting mixture was allowed to warm gradually to ambient temperature and then diluted with ethylacetate (100 mL). The layers were separated, and the aqueous layer was extracted with ethylacetate (2x100 mL). The combined organic extracts were washed with sat. $NaHCO_3$ solution and brine solution, dried over anhydrous Na_2SO_4, filtered and concentrated under reduced pressure. The obtained crude residue was purified by flash column chromatography over silica gel using petroleum ether and ethylacetate as eluents (10-20% ethylacetate in petroleum ether) to provide the product **4** as a pale-yellow oil.

Yield: 470 mg (81 %). R_f: 0.48 (Pet. ether /EtOAc 3:2, visualized with 1.3% ninhydrin solution). ^{1}H NMR ($CDCl_3$, 500 MHz, ppm) δ 5.83 (ddt, *J* = 17.1, 10.2, 7.1 Hz, 1H), 5.17 – 5.13 (m, 1H), 5.12 (dd, *J* = 3.3, 1.5 Hz, 1H), 4.78 (br.s, 1H), 3.73 – 3.67 (m, 2H), 2.27 – 2.12

(m, 2H), 1.44 (s, 9H), 1.11 (d, J = 6.8 Hz, 3H).^{13}C NMR ($CDCl_3$, 126 MHz, ppm) δ 155.9, 134.8, 118.2, 79.6, 73.4, 50.4, 38.4, 28.5, 14.7. ESI-MS *m/z* calcd for $C_{11}H_{22}NO_3$ $[M+H]^+$ 216.16; observed: 214.2. [5,6]

Synthesis of (*S*)-*tert*-butyl-(3-oxohept-6-en-2-yl)carbamate (7)

The 1-butylmagnesium bromide solution was prepared as follows, A magnesium powder (510 mg, 21.2 mmol) was suspended in dry diethylether (2 mL) at ambient temperature under argon atmosphere and the resulting mixture was subsequently treated with drops of 1,2-diromoethane. The resulting mixture was allowed to stir at the same conditions for 10 min and then a solution of 4-bromo-1-butene (0.71 g, 5.3 mmol, 1M in dry diethylether) was dropwisely added over a period of 10 min. The resulting reaction mixture was allowed to stir at 35ºC for 1h to afford a transparent solution of 1-butylmagnesium bromide. The solution was used directly in the next step.

To a stirred solution of Weinreb amide **2** (0.34 g, 1.5 mmol) in dry diethylether (15 mL) under argon atmosphere at 0ºC was added dropwise a fresh solution of 1-butylmagnesium bromide (5.3 mmol, 1M in diethylether) over a period of 20 min. The resulting reaction mixture was allowed to stir at the same conditions for 30 min and for additional 2h at ambient temperature (as monitored by TLC analysis; Pet. ether / EtOAc 4:1; $R_{f\,(adduct)}$= 0.2; $R_{f\,(product)}$=0.55; visualized with 1.3% ninhydrin solution). The reaction was diluted with diethylether (100 mL) and subsequently quenched with an ice-cold 1M HCl (100mL). The layers were separated, and the aqueous layer was extracted with diethylether (2x 80 mL). The organic layers were combined, washed with sat. $NaHCO_3$ solution and brine, dried over anhydrous Na_2SO_4, filtered, and concentrated *in vacuo*. Purification of the obtained residue by flash column chromatography over silica gel using petroleum ether and ethylacetate as eluents (10-15% ethylacetate in petroleum ether) afforded the desired product **7** as a white solid.

Yield: 210 mg (71 %). R_f: 0.55 (Pet. ether /EtOAc 4:1, visualized with 1.3% ninhydrin solution). ^{1}H NMR ($CDCl_3$, 500 MHz, ppm) δ 5.79 (ddt, J = 16.8, 10.2, 6.5 Hz, 1H), 5.24 (br.s, 1H), 5.03 (ddd, J = 17.1, 3.2, 1.6 Hz, 1H), 4.98 (dd, J = 10.2, 1.4 Hz, 1H), 4.31 (p, J = 7.0 Hz, 1H), 2.63 (dt, J = 17.2, 7.5 Hz, 1H), 2.59 – 2.52 (m, 1H), 2.35 (dt, J = 14.0, 4.3 Hz, 2H), 1.43 (s, 9H), 1.32 (d, J = 7.2 Hz, 3H). ^{13}C NMR ($CDCl_3$, 126 MHz, ppm) δ 209.0, 155.3, 136.9, 115.7, 79.9, 55.2, 38.4, 28.5, 27.6, 18.0. ESI-MS *m/z* calcd for $C_{12}H_{22}NO_3$ $[M+H]^+$ 228.16; observed: 228.2. [7]

Synthesis of (*tert*-butoxycarbonyl)glycine (13)

A stirred solution of glycine (1.1 g, 14.8 mmol) in aqueous 1M NaOH solution (50 mL) at 0°C was treated in dropwise with a solution of di-*tert*-butyl dicarbonate (3.4 g, 16.3 mmol, in dioxane) over a period of 20 min. After the resulting reaction mixture was stirred at the same conditions for 1h and for additional 12h at ambient temperature, the solvent was removed under reduced pressure. The mixture was washed with n-hexane (50 mL) to remove the unreacted Boc_2O. The aqueous residue was cooled again to 0ºC and was carefully acidified by 1M $KHSO_4$ solution. The obtained mixture was diluted with ethylacetate (100 mL) and the layers were separated. The aqueous layer was extracted with ethylacetate (2x100 mL), and the combined

organic extracts were dried over anhydrous Na_2SO_4, filtered, and concentrated. The obtained crude product **13** was pure as determined by NMR- analysis and was used directly in the next step without any further purification.

Yield: 2.3 mg (89%). ^{1}H NMR ($CDCl_3$, 500 MHz, ppm): δ 6.78 (br.s, 1H), 3.97 (br.s, 2H), 1.45 (s, 9H). ^{13}C NMR ($CDCl_3$, 126 MHz, ppm): δ 178.2, 155.2, 80.6; 43.7, 28.4. ESI-LCMS: *m/z* calcd for $C_7H_{13}NO_4$ $[M+H]^+$ 176.09; observed 176.1. [4,8]

Synthesis of *tert*-butyl (2-(methoxy(methyl)amino)-2-oxoethyl)carbamate (14)

To a stirred solution of *N*-Boc-glycine **13** (1.2 g, 6.75 mmol) in dry dichloromethane (25 mL) under argon atmosphere at -10°C was added *N*-methylmorpholine (2.3 mL, 20.3 mmol), followed by *N,O*-dimethylhydroxylamine hydrochloride (0.79 g, 8.1 mmol). The resulting mixture was treated at the same conditions with 1-ethyl-3-(3-dimethylaminopropyl)carbodiimide hydrochloride (1.55 g, 8.1 mmol) in portionwise over a period of 30 min. After being stirred at the same conditions for 2h (as detected by TLC analysis; EtOAc 100%; $R_{f(adduct)}$= 0.1; $R_{f(product)}$=0.54; visualized with 1.3% ninhydrine solution), the reaction was quenched with sat. NH_4Cl (100 mL). The mixture was diluted with DCM and the layers were separated. The aqueous layer was extracted again with DCM (2x80 mL), and the combined organic layers were washed with sat. $NaHCO_3$ solution and brine, dried over anhydrous Na_2SO_4, filtered, and concentrated to afford a white solid of the desired product **14**. The obtained product was pure as determined by LCMS- and NMR-analysis and was directly used in the next step without any further purification.

Yield: 1.15 mg (78%). R_f: 0.54 (EtOAc 100%, visualized with 1.3% ninhydrine solution). ^{1}H NMR ($CDCl_3$, 500 MHz, ppm): δ 5.28 (br.s, 1H), 4.09 (br.s, 2H), 3.71 (s, 3H), 3.22 (s, 3H), 1.46 (s, 9H). ^{13}C NMR ($CDCl_3$, 126 MHz, ppm): δ 173.8, 155.2, 79.6, 61.4, 41.7, 32.4, 28.4. ESI-LCMS: *m/z* calcd for $C_9H_{19}N_2O_4$ $[M+H]^+$ 219.13; observed 219.1.[9]

Synthesis of (*S*)-*tert*-butyl-3-oxopent-4-en-2-ylcarbamate (18)

A stirred solution of compound **2** (3 g, 13 mmol) in dry THF (65 mL) under an argon atmosphere at -20°C was added dropwise a solution of vinylmagnesium bromide (45.5 mL, 45.5 mmol, 1M solution in THF); the addition rate was adjusted so as to keep the internal temperature at -20 °C and it took 45 min to complete. The resulting reaction mixture was allowed to stir at the same conditions for 30 min and for additional 3h at ambient temperature (as judged by TLC analysis; Pet. ether/ EtOAc 4:1; $R_{f\ (adduct)}$= 0.2; $R_{f\ (product)}$=0.54;visualized with 1.3% ninhydrin). The reaction mixture was added dropwisely to an ice-coold 1M HCl solution to quench; again, so as to keep the temperature of solution below -10°C. The resultant mixture was diluted with ethylacetate and the layers were separated. The aqueous layer was extracted with ethylacetate (2x100 mL), and the combined organic extracts were subsequently washed with saturated $NaHCO_3$ solution and brine, dried over anhydrous Na_2SO_4, filtered and concentrated *in vacuo*. The resultant residue was purified by flash column chromatography over silica gel using cyclohexane and ethylacetate as eluents (from 5-15% ethylacetate in cyclohexane) to afford the desired product **18** as a white solid.

Yield: 1.7 g (66 %). R_f: 0.54 (cyclohexane/ethylacetate 4:1, visualized with 1.3% ninhydrine solution). ^{1}H NMR ($CDCl_3$, 500 MHz, ppm): δ 6.48 (dd, J = 17.4, 9.9 Hz, 1H). 6.37 (dd, J =

17.4, 1.9 Hz, 1H), 5.88 (dd, J = 10.2, 1.9 Hz, 1H), 5.37 (br s, 1H), 4.54-4.67 (m, 1H), 1.44 (s, 9H), 1.33 (d, J = 6.9 Hz, 3H). ^{13}C NMR ($CDCl_3$, 126 MHz, ppm): δ 198.7, 155.2, 132.9, 130.3, 79.5, 53.2, 28.6, 18.4. ESI-LCMS *m/z* calcd for $C_{10}H_{18}NO_3$ $[M+H]^+$ 200.13; observed 200.1. [2,4]

Synthesis of (2*S*,3*R*)-*tert*-butyl-(3-hydroxypent-4-en-2-yl)carbamate (19)

To a stirred solution of compound **18** (1.5 g, 7.5 mmol) in anhydrous ethanol (15 mL) under an argon atmosphere at -78°C was added in portionwise lithium tri-(*tert*-butoxy)-aluminum hydride (4.8 g, 18.75 mmol); the addition rate was adjusted so as to keep the internal temperature below -65°C and it took 30 min to complete. After the resulting reaction mixture was allowed to stir at the same conditions for 3h (as judged by TLC analysis; Pet. ether/ EtOAc 4:1; $R_{f\,(adduct)}$= 0.54; $R_{f\,(product)}$=0.42; visualized with 1.3% ninhydrine), an ice-coold 1M HCl solution (100 mL) was added dropwise to quench the reaction; again, so as to keep the internal temperature below -65°C and it took 30 min to complete. The resulting mixture was diluted with ethylacetate and was allowed to gradually warm to ambient temperature. The layers were separated, and the aqueous layer was subsequently extracted with ethylacetate (2x 100mL). The combined organic layers were sequentially washed with saturated $NaHCO_3$ solution and brine, dried over anhydrous Na_2SO_4, filtered and concentrated under reduced pressure. Flash column chromatography of the resultant crude mixture over silica gel using cyclohexane and ethylacetate as eluents (from 15-25% ethylacetate in cyclohexane) afforded the desired product **19** as a white solid.

Yield: 1.1 g (73%). R_f: 0.42 (cyclohexane/ethylacetate 4:1, visualized with $KMnO_4$ solution). ^{1}H NMR ($CDCl_3$, 500 MHz, ppm): δ 5.85 (ddd, J= 17.2, 10.6, 5.5 Hz, 1H), 5.33 (dd, J = 17.2, 1.6 Hz, 1H), 5.24 (dd, J = 10.5, 1.5 Hz, 1H), 4.76-4.62 (m, 1H), 4.23-4.16 (m, 1H), 2.85 (br. s, 1H), 1.45 (s, 9H), 1.09 (d, J = 6.9 Hz, 3H). ^{13}C NMR ($CDCl_3$, 126 MHz, ppm): δ 156.5, 137.0, 116.7, 79.9, 75.9, 50.9, 28.5, 15.5. ESI-LCMS: *m/z* calcd for $C_{10}H_{19}NO_3Na$ $[M+Na]^+$ 224.13; observed 224.1. [2,4]

Synthesis of 10-bromo-1-decene

A stirred solution of 1,10-dibromodecane (7 g, 23.3 mmol) in anhydrous THF (200 mL) under an argon atmosphere was treated in portionwise with *tert*-BuOK (2.9 g, 25.6 mmol) over a period of 30 min. After being stirred at 50°C for 12h (as detected by TLC analysis; Pet. ether; $R_{f\,(adduct)}$= 0.58; $R_{f\,(product)}$=0.89; visualized with $KMnO_4$ solution), the reaction mixture was cooled to 0°C and subsequently quenched with water. The resulting mixture was diluted with diethylether, and the layers were separated. The aqueous layer was extracted with diethylether (2x100 mL), and the combined organic layers were washed with brine, dried over anhydrous Na_2SO_4, filtered and concentrated under reduced pressure. Purification of the obtained crude product by flash column chromatography over silica gel using petroleum ether as eluent provided the desired product 10-bromo-1-decene as colorless oil.

Yield: 3.1 g (61%). R_f: 0.84 (*n*-hexane 100%, visualized with $KMnO_4$ solution). ^{1}H NMR ($CDCl_3$, 500 MHz, ppm): δ 5.81 (ddt, J = 16.9, 10.1, 6.7 Hz, 1H), 5.04 – 4.91 (m, 2H), 3.41 (t, J = 6.9 Hz, 2H), 2.03 (dt, J = 7.6, 4.0 Hz, 2H), 1.92 – 1.78 (m, 2H), 1.49 – 1.28 (m, 10H). ^{13}C NMR ($CDCl_3$, 126 MHz, ppm): δ 139.3, 114.3, 34.2, 33.9, 33.0, 29.4, 29.1, 29.0, 28.9, 28.3. [2]

Synthesis of (2*S*,3*R*,4*E*)-*tert*-butyl-(13-bromo-3-hydroxytridec-4-en-2-yl)carbamate (20)

A stirred mixture of aminoalcohol derivative **19** (660 mg, 3.25 mmol) and 10-bromo-1-decene (2.9 g, 13 mmol) in anhydrous d-chloroform (10 mL) under an argon atmosphere was treated with a catalytic amount of *p*-benzoquinone (10 mol%) followed by Grubbs Catalyst 2nd Generation (10 mol%, in one portion). After the resulting reaction mixture was stirred under reflux for 12 h (as detected by TLC analysis, until no further change in the composition of the reaction mixture, Pet. ether/ EtOAc 4:1; $R_{f\ (adduct)}$= 0.42; $R_{f\ (product)}$=0.51; visualized with 1.3% ninhydrine solution), the solvent was removed under reduced pressure. Flash column chromatography for the obtained residue over silica gel using cyclohexane and ethylacetate as eluents (from 10-25% ethylacetate in cyclohexane) provided the desired product **20** as a colorless oil.

Yield: 725 mg (57%). R_f: 0.51 (Pet. ether /ethylacetate 4:1, visualized with 1.3% ninhydrine solution). ^{1}H NMR ($CDCl_3$, 500 MHz, ppm): δ 5.70 (dtd, *J* = 15.0, 6.8, 1.1 Hz, 1H), 5.43 (dd, *J* = 15.4, 6.6 Hz, 1H), 4.10 (d, *J* = 2.4 Hz, 1H), 3.83-3.73 (m, 1H), 3.40 (t, *J* = 6.9 Hz, 2H), 2.04 (q, *J* = 7.0 Hz, 2H), 1.88 – 1.81 (m, 2H), 1.44 (s, 9H), 1.42 – 1.27 (m, 10H), 1.07 (d, *J* = 6.9 Hz, 3H). ^{13}C NMR ($CDCl_3$, 126 MHz, ppm): δ 156.4, 134.0, 128.6, 79.8, 75.9, 51.2, 34.2, 32.9, 32.5, 29.4, 29.2, 29.2, 28.8, 28.5, 28.3, 15.7. ESI-MS: *m/z* calcd for $C_{18}H_{35}NO_3Br$ $[M+H]^+$ 392.18; observed 392.2. [4]

(*S*)-2-[(*tert*-Butoxycarbonyl)-amino]-3-hydroxypropionic acid (26)

A stirred solution of L-serine (2.5 g, 23.75 mmol) in aqueous 1M NaOH (25 mL) at -15°C was treated with a solution of di-*tert*-butyldicarbonate (6.2 g, 28.5 mmol) in dioxane (25 mL) dropwise over a period of 20 min. After being stirred at the same conditions for 1h and for additional 3h at ambient temperature, the reaction mixture was washed two times with n-hexane (2×60 mL) to remove excess of unreacted Boc_2O. The aqueous layer was then cooled to -15°C and carefully acidified with a solution of 1M $KHSO_4$ (to pH≈2-3). The resulting mixture was subsequently extracted with ethylacetate (4×80 mL), and the combined organic extracts were dried over anhydrous Na_2SO_4, filtered and concentrated under reduced pressure to provide *N*-Boc-L-serine **26** as colourless oil. The product was used in the next step without any further purification. Yield: 4.5 g (92%). R_f: 0.2 (EtOAc 100%, visualized with 1.3% ninhydrine). The spectroscopic data of the product were consistent with those reported in literature**.** [2]

(*S*)-*tert*-butyl-1-(*N*-methoxy-*N*-methylcarbamoyl)-2-hydroxyethylcarbamate (27)

To a stirred solution of *N*-Boc-L-serine **26** (3 g, 14.6 mmol) in dry DCM (30 mL) at -15°C under argon atmosphere was added *N,O*-dimethylhydroxylamine hydrochloride (1.6 g, 16.1 mmol), followed by addition of *N*-methylmorpholine (1.8 mL, 16.1 mmol, until pH≈8-9). To the resulting mixture, 1-ethyl-3-(3-dimethylaminopropyl)-carbodiimide hydrochloride (EDCI.HCl) (3.1 g, 16.1 mmol) was added portionwise over a period of 30 min. After the resulting reaction mixture was allowed to stir for 90 min at -15°C (as judged by TLC analysis, EtOAc 100%; $R_{f(adduct)}$= 0.2; $R_{f(product)}$=0.5; visualized with 1.3% ninhydrine), an ice-cold 1M

HCl solution (100 mL) was carefully added to quench the reaction. The resulting mixture was subsequently diluted with CH_2Cl_2 (100 mL) and the layers were separated. The aqueous layer was extracted with CH_2Cl_2 (3×80 mL). The combined organic layers were sequentially washed with saturated $NaHCO_3$ solution (100 mL) and brine (100 mL), dried over anhydrous Na_2SO_4, filtered and concentrated under reduced pressure to provide compound **27** as a white solid. The product was used in the next step without any further purification.

Yield: 3.1 g, (86%). R_f: 0.16 (EtOAc 100%, visualized with 1.3% ninhydrine). ^{1}H NMR ($CDCl_3$, 500 MHz, ppm): δ 1.44 (s, 9H), 3.22 (s, 3H), 3.77 (s, 3H), 3.80-3.82 (m, 2H), 4.79 (b.s, 1H), 5.65 (d, *J*=5.7 Hz, 1H), ^{13}C-NMR ($CDCl_3$, 125 MHz, ppm) δ 28.4, 32.2, 52.5, 61.7, 63.8, 80.2, 156.0, 171.1. ESI-HRMS *m/z* calcd for $C_{10}H_{20}N_2O_5Na$ $[M+Na]^+$ 271.1269; observed 271.1264. [2]

11-((tetrahydro-2H-pyran-2-yl)oxy)undecan-1-ol (31)

A stirred solution of 1,11-undecandiol (11.8 g, 63 mmol) in dry tetrahydrofuran (200 mL) at 0°C under argon atmosphere was treated with a catalytic amount of *p*-toluenesulfonic acid monohydrate (PTSA) (0.95 g, 5.04 mmol, 8 mol%), followed by dropwise addition of 3,4-dihydropyran solution (5.9 mL, 69.3 mmol, 1M in dry THF). After being stirred at the same conditions for 1h, gradually warmed to ambient temperature and stirred for additional 16h (as judged by TLC analysis; cyclohexane/ EtOAc 6:4; $R_{f\,(adduct)}$= 0.36; $R_{f\,(product)}$=0.67; visualized with ceric ammonium molybdate solution), the reaction mixture was diluted with diethylether (100 mL) and carefully quenched at 0°C with saturated aqueous $NaHCO_3$ solution (150 mL). The layers were separated and the aqueous layer was extracted with diethylether (3x100 mL). The combined organic extracts was washed with brine solution (200 mL), dried over anhydrous Na_2SO_4, filtered and concentrated *in vacuo*. Purification of the obtained crude product by flash column chromatography over silica gel using cyclohexane and ethylacetate as eluents (from 18-26% ethylacetate in cyclohexane) provided compound **31** as colorless oil.

Yield: 9.6 g (56%; **note**, the di-protected diol byproduct was isolated in ~6% yield). R_f: 0.52 (cyclohexane/EtOAc 7:3, ceric ammonium molybdate solution). ^{1}H NMR (500 MHz, $CDCl_3$, ppm) δ 4.53 (1H, t, *J* =3.2 Hz), 3.75-3.71 (m, 2H), 3.67-3.62 (m, 2H), 3.46 (2H, t, *J* =6.5 Hz), 1.86-1.69 (m, 8H), 1.61-1.23 (m, 16H). ^{13}C NMR (126 MHz, $CDCl_3$, ppm) δ 98.8, 67.6, 62.9, 62.2, 32.7, 30.6, 29.7, 29.6, 29.5, 29.5, 29.4, 29.3, 26.2, 25.7, 25.4, 19.5. MS (ESI^+) *m/z* calcd for $C_{16}H_{33}O_3$ $[M+H]^+$ 273.24; observed 273.2. [10]

2-((11-bromoundecyl)oxy)tetrahydro-2H-pyran (32)

To a stirred solution of alcohol **31** (9.1 g, 33.52 mmol) and CBr_4 (12.2 g, 36.9 mmol) in anhydrous dichloromethane (335 mL) at 0°C under an argon atmosphere was added dropwise a solution of triphenyl phosphine (10.1 g, 38.5 mmol, 39 mL 1M in dry dichloromethane) over a period of 20 min. After the resulting reaction mixture was allowed to stir for 1h at 0°C, gradually warmed to ambient temperature, and stirred for additional 3h (as monitored by TLC analysis; Pet. ether/ EtOAc 8:2; $R_{f\,(adduct)}$= 0.37; $R_{f\,(product)}$=0.68; visualized with ceric ammonium molybdate solution), the solvent was removed under reduced pressure. The resultant oily residue was suspended in petroleum ether (100 mL) and the mixture was stirred for 30 min at ambient temperature, during while a white precipitate was formed. The mixture

was filtered through a pad of Celite to remove the formed triphenylphosphonium oxide and the combined filtrates was concentrated under reduced pressure. This process was repeated several times until no further precipitate was formed. The obtained oily crude product was subsequently purified by flash column chromatography over silica gel using petroleum ether and ethylacetate as eluents (from 5-15% ethylacetate in petroleum ether) to yield compound **32** as colorless oil.

Yield: 9.64 g (86 %). R*f*: 0.53 (petroleum ether /EtOAc 9:1, ceric ammonium molybdate solution). These data are in accordance with literature precedence [1,4].^{1}H NMR (500 MHz, $CDCl_3$, ppm) δ 4.59-4.56 (m, 1H), 3.92-3.86 (m, 1H), 3.76-3.69 (m, 1H), 3.54-3.49 (m, 1 H), 3.42 (t, *J*= 7.1 Hz, 2H), 3.39-3.36 (m, 1H), 1.89-1.52 (m, 8H), 1.42-1.27 (m, 16H). ^{13}C NMR (126 MHz, $CDCl_3$, ppm) δ 98.9, 67.8, 62.5, 34.1, 32.9, 30.8, 29.9, 29.7, 29.6, 29.6, 28.9, 28.2, 26.4, 25.6, 19.8. MS (ESI^+) *m/z* calcd for $C_{16}H_{32}O_2Br$ $[M+H]^+$ 335.16; observed 335.2. [11]

11-bromoundec-1-ene (35)

A stirred solution of 10-undecenol **34** (1.05 g, 6.15 mmol) and CBr_4 (2.25 g, 6.8 mmol) in dry dichloromethane (62 mL) at 0°C under an argon atmosphere was treated with a solution of triphenyl phosphine (1.85 g, 7.1 mmol, 7 mL 1M in dry dichloromethane) dropwise over a period of 20 min. The resulting reaction mixture was allowed to stir at the same conditions for 1h and for additional 3h at ambient temperature (as judged by TLC analysis; Pet. ether/ EtOAc 4:1; $R_{f\ (adduct)}$= 0.48; $R_{f\ (product)}$=1; visualized with $KMnO_4$ solution), before it was filtered through a pad of silica gel and rinsed subsequently with petroleum ether. The combined filtrates was concentrated under reduced pressure and the resultant oily crude product was purified by flash column chromatography over silica gel using petroleum ether as eluent to afford 11-bromoundec-1-ene **35** as colorless oil.

Yield: 1.3 g (91 %). R*f*: 0.57 (Pet. ether, visualized with $KMnO_4$ solution). ^{1}H NMR (500 MHz, $CDCl_3$, ppm) δ 5.82 (ddt, *J* = 16.7, 6.8 Hz, 1H), 4.96 (ddd, *J* = 17.1, 3.7, 1.6 Hz, 1H), 4.93 (ddt, *J* = 10.2 Hz, 1H), 3.43 (t, *J*= 6.8 Hz, 2H), 2.14-2.05 (m, 2H), 1.96-1.88 (m, 2H,), 1.47-1.26 (m, 10H). ^{13}C NMR (126 MHz, $CDCl_3$, ppm) δ 139.3, 114.3, 34.1, 33.9, 32.9, 29.6, 29.2, 29.0, 28.9, 28.5. [12]

(*S*)-2-methylbutyl 4-methylbenzenesulfonate (37)

To a stirred solution of (*S*)-2-methylbutan-1-ol (2 g, 22.4 mmol) in dry dichloromethane (45 mL) under argon atmosphere at 0ºC was added pyridine (7.2 mL, 89.6 mmol), followed by a portionwise addition of tosyl chloride (5.3 g,28 mmol). The resulting reaction mixture was allowed to stir for 4h at ambient temperature (as monitored by TLC analysis; Pet. ether/ EtOAc 7:3; $R_{f\ (adduct)}$= 0.38; $R_{f\ (product)}$=0.79; visualized with ceric ammonium molybdate solution), before it was carefully quenched with saturated $NaHCO_3$ solution (100 mL). The resultant mixture was extracted with n-pentane (3x 100 mL) and the combined organic layers were washed with saturated $CuSO_4$ solution (150 mL) and brine (150 mL), dried over anhydrous Na_2SO_4, filtered and concentrated (**Caution**: temperature of rotatory evaporator shouldn't exceed 35°C). Purification of the crude product with flash column chromatography over silica gel using n-pentane and ethylacetate as eluents (from 0-9% ethylacetate in petroleum ether) afforded the desired product **37** as colorless oil.

Yield: 3.9 g (72 %). R*f*: 0.53 (petroleum ether /EtOAc 9:1, ceric ammonium molybdate solution). ^{1}H NMR (300 MHz, $CDCl_3$, ppm) δ 7.82 (d, *J*= 8.5 Hz, 2H), 7.36 (d, *J*= 8.1 Hz, 2H), 3.74 (d, *J*= 6.1 Hz, 2H), 2.46 (s, 3H), 1.74-1.61 (m, 1H), 1.48-1.09 (m, 2H), 0.86 (d, *J*= 6.9, 3H), 0.82 (t, *J*= 7.5 Hz, 3H). ESI-MS *m/z* calcd for $C_{12}H_{18}NaO_3S$ $[M+Na]^+$: 265.09; observed 265.1. [13]

(*S*)-1-bromo-2-methylbutane (38)

A stirred solution of (*S*)-2-methylbutyl 4-methylbenzenesulfonate **37** (3.1 g, 12.7 mmol) and *N*-bromosuccinimide (NBS) (2.5 g, 14 mmol) in anhydrous dichloromethane (125 mL) at 0°C under argon atmosphere was treated with a solution of triphenyl phosphine (PPh_3) (3.8 g, 14.6 mmol) in anhydrous dichloromethane (15 mL) over a period of 20 min. After the resulting reaction mixture was allowed to stir for 2h at ambient temperature (as judged by TLC analysis for almost a complete reaction, Pet. ether/ EtOAc 9:1; $R_{f\,(adduct)}$= 0.53; $R_{f\,(product)}$=1; visualized with $KMnO_4$ solution), the reaction mixture was filtered through a pad of silica gel and rinsed subsequently with n-pentane. The solvent was removed under reduced pressure (**Caution**: temperature of rotatory evaporator shouldn't exceed 25°C), and the resultant residue was subsequently purified by flash column chromatography over silica gel using n-pentane as eluent to furnish the desired compound **38** as colorless oil.

Yield: 1.3 g (68 %). R*f*: 0.57 (petroleum ether, visualized with $KMnO_4$ solution). ^{1}H NMR (500 MHz, $CDCl_3$, ppm) δ 3.41 (dd, *J*= 9.8 Hz, 1H), 3.37 (dd, *J*= 9.8, 5.6 Hz, 1H), 1.79-1.71 (m, 1 H), 1.56-1.44 (m, 1H), 1.26 (q, *J* = 7.4 Hz, 1H) 1.02 (d, *J*= 6.7 Hz, 3H), 0.93 (t, *J*= 7.6 Hz, 3H). ^{13}C NMR (126 MHz, $CDCl_3$, ppm) δ 41.2, 37.2, 27.6, 18.7, 11.4. [14]

(4*S*,5*R*)-*tert*-Butyl-4-(hydroxymethyl)-2,2-dimethyl-5-vinyloxazolidine-3-carboxylate (40)

The titled compound was synthesized in 7 steps starting from L-serine according to our previously established route [2]. R*f*: 0.5 (cyclohexane/EtOAc 6:4, visualized with 1.3% ninhydrine). ^{1}H NMR ($CDCl_3$, 500 MHz, ppm) δ 5.94-5.76 (m, 1H), 5.37-5.28 (m, 1H), 5.23-5.19 (m, 1H), 4.54-4.48 (m, 1H), 3.93-3.75 (m, 1H), 3.56 (dd, *J*= 5.8, 11.1 Hz, 1H), 3.37 (m, 1H), 1.45-1.39 (m, 6H), 1.27 (br.s, 9H). ^{13}C NMR ($CDCl_3$, 300 MHz, ppm): δ 154.4, 131.9, 119.3, 93.3, 81.3, 76.5, 63.5, 61.8, 28.5, 24.8. MS (ESI^+) *m/z* calcd for $C_{13}H_{23}NNaO_4$ $[M+Na]^+$ 280.15; observed 280.1. [2]

3 NMR spectra of synthesized compounds

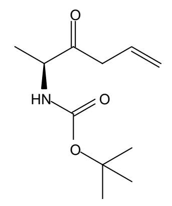

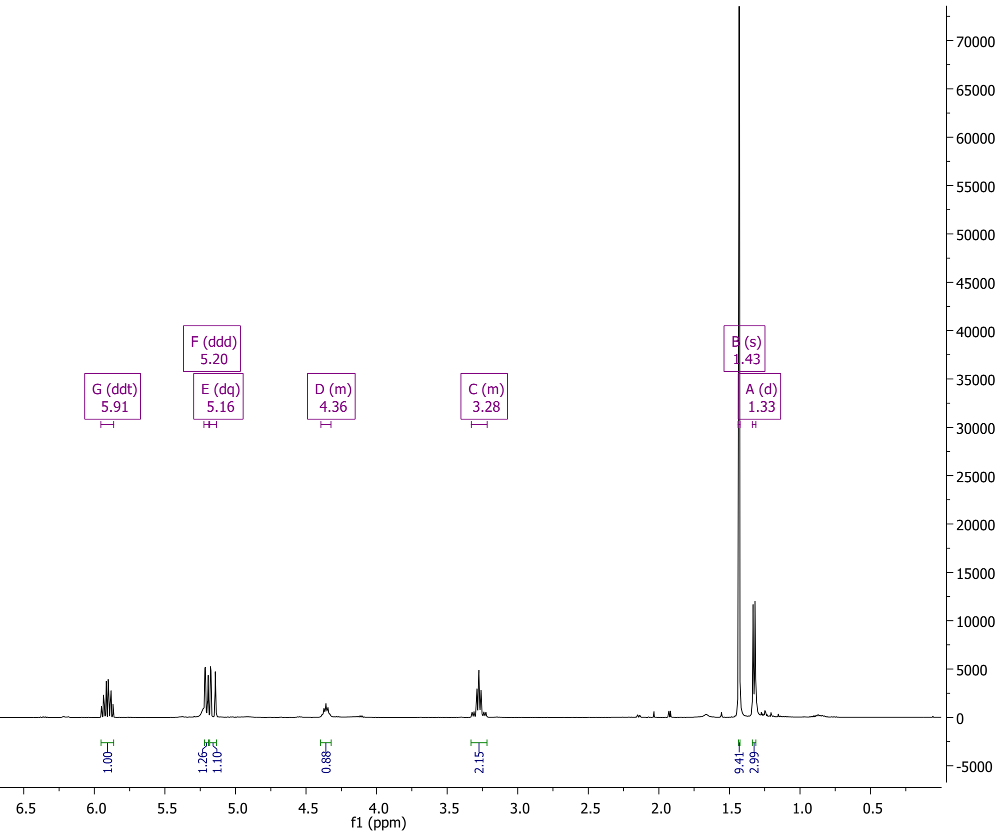

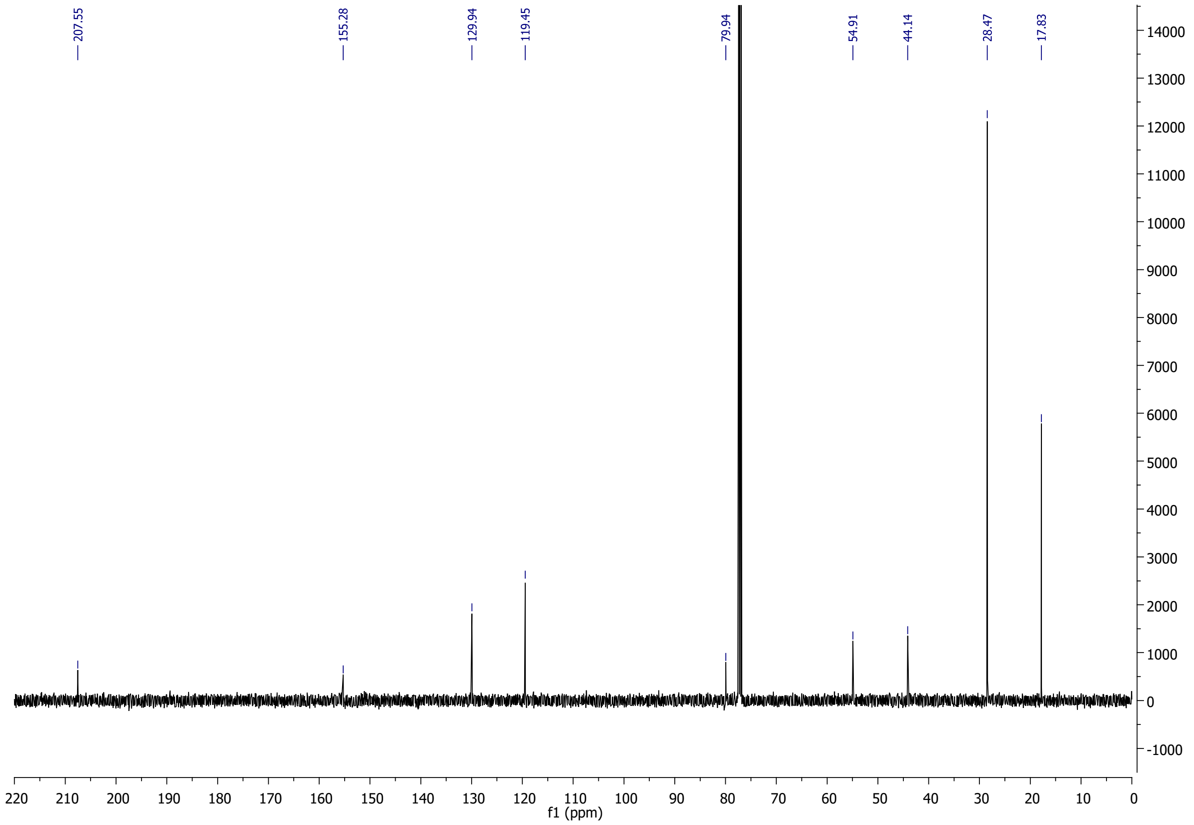

^{1}H- and ^{13}C- NMR Spectra for compound 3

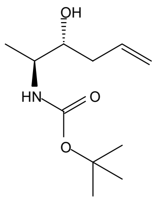

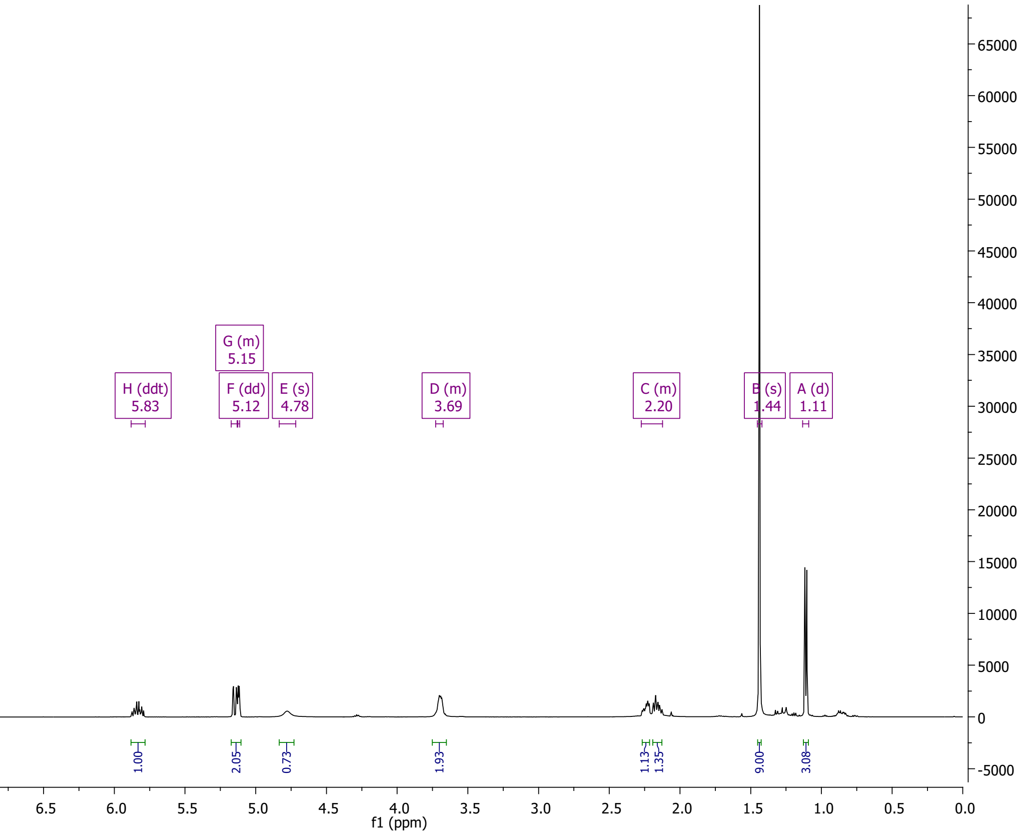

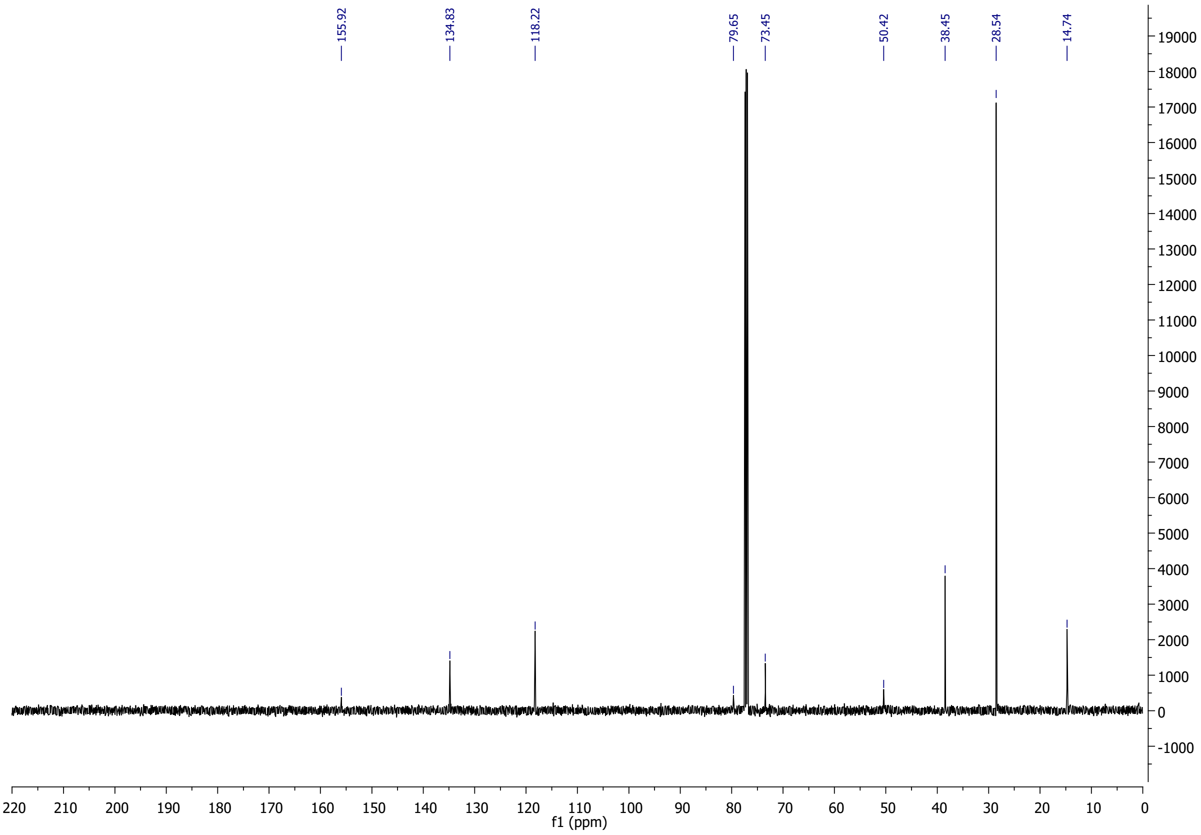

^{1}H- and ^{13}C- NMR Spectra for compound 4

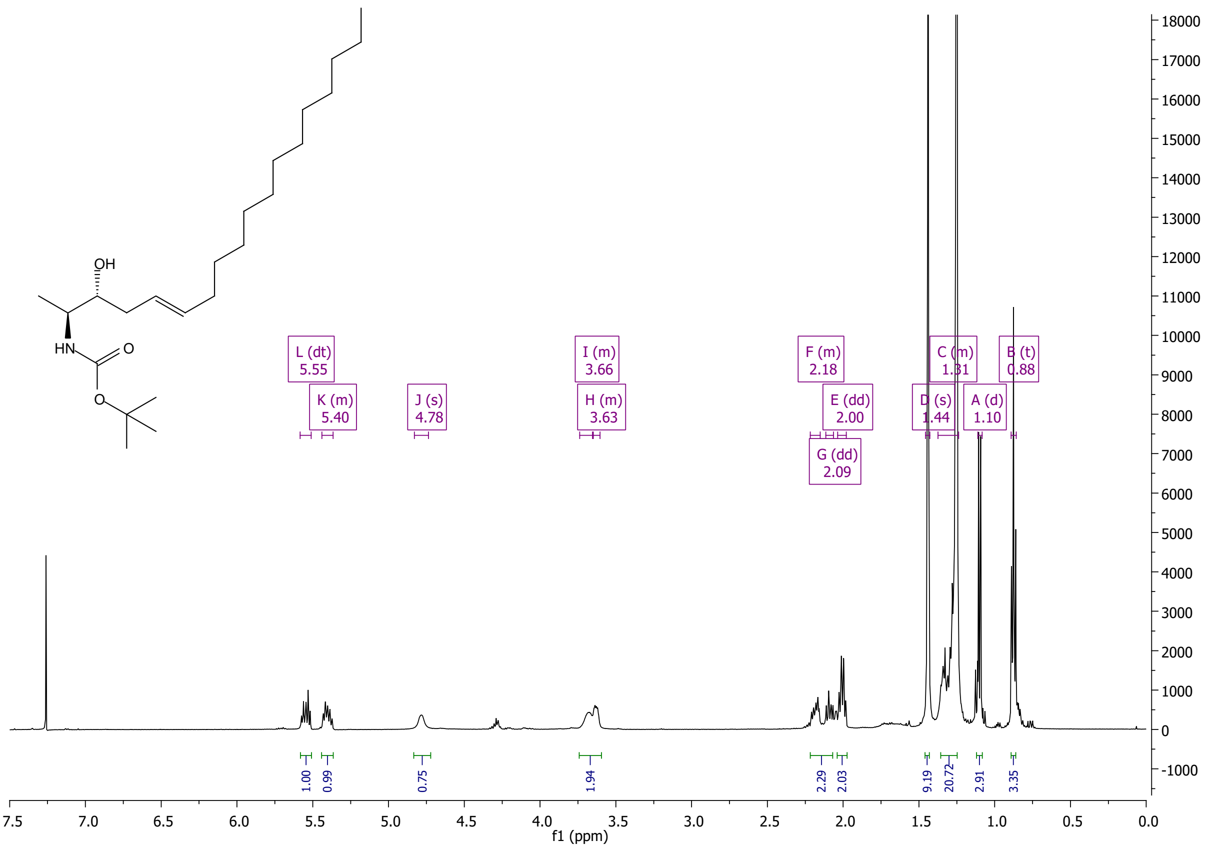

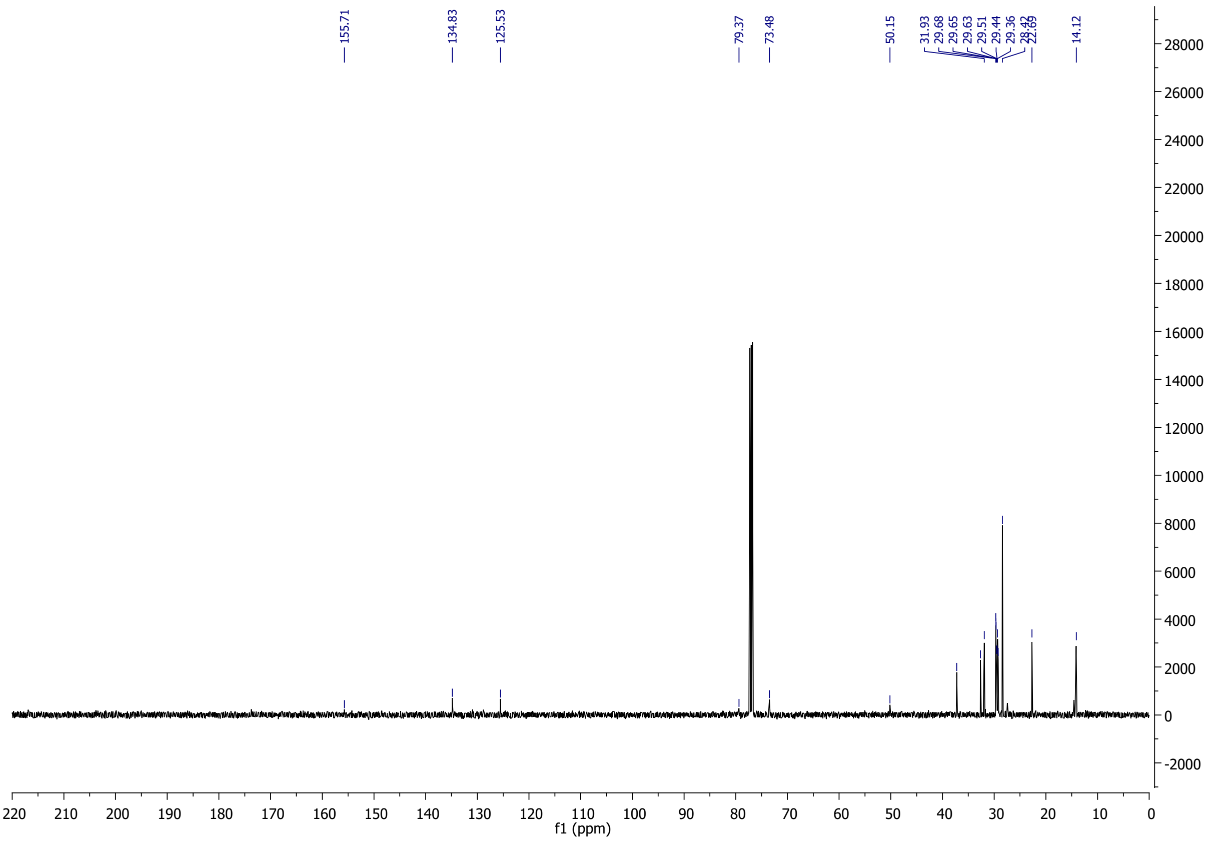

^{1}H- and ^{13}C- NMR Spectra for compound 5

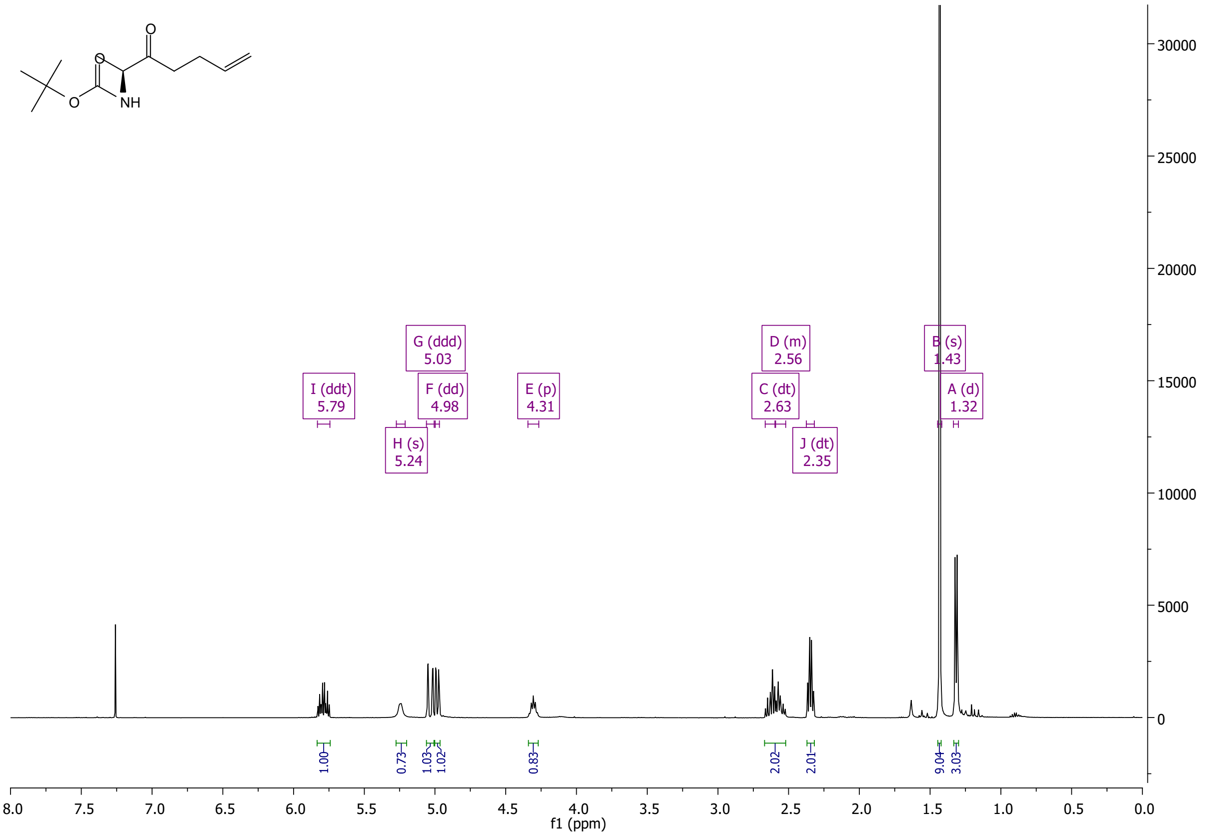

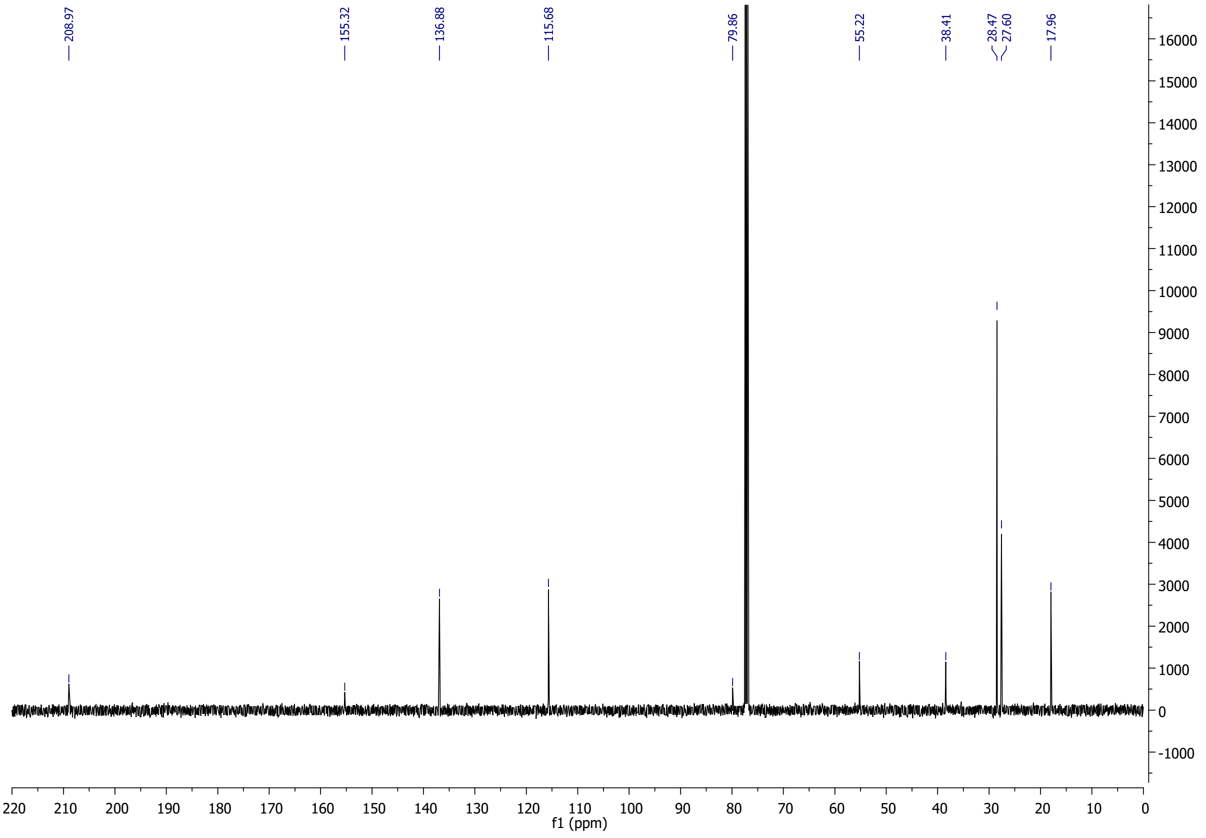

^{1}H- and ^{13}C- NMR Spectra for compound 7

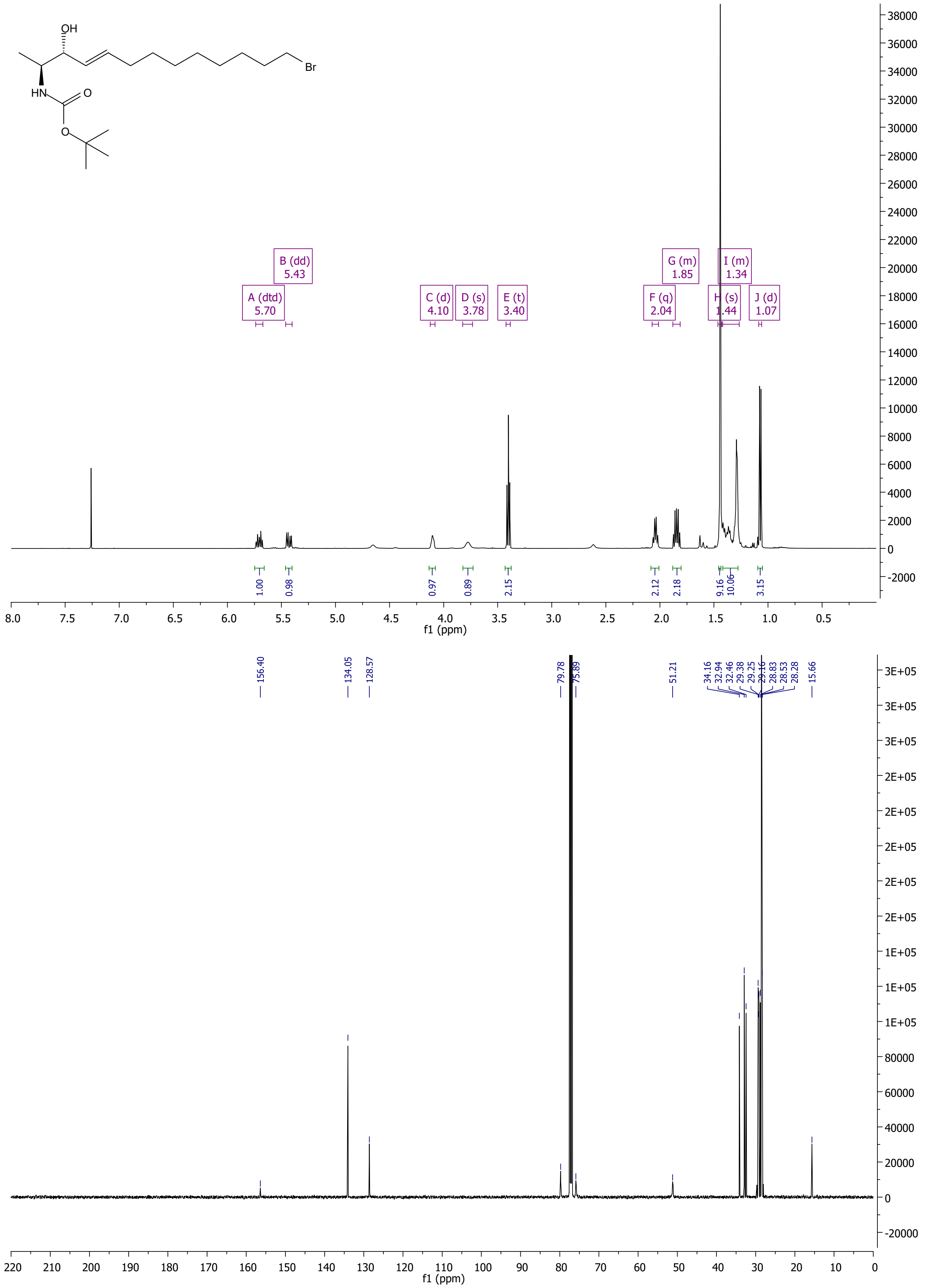

^{1}H- and ^{13}C- NMR Spectra for compound 20

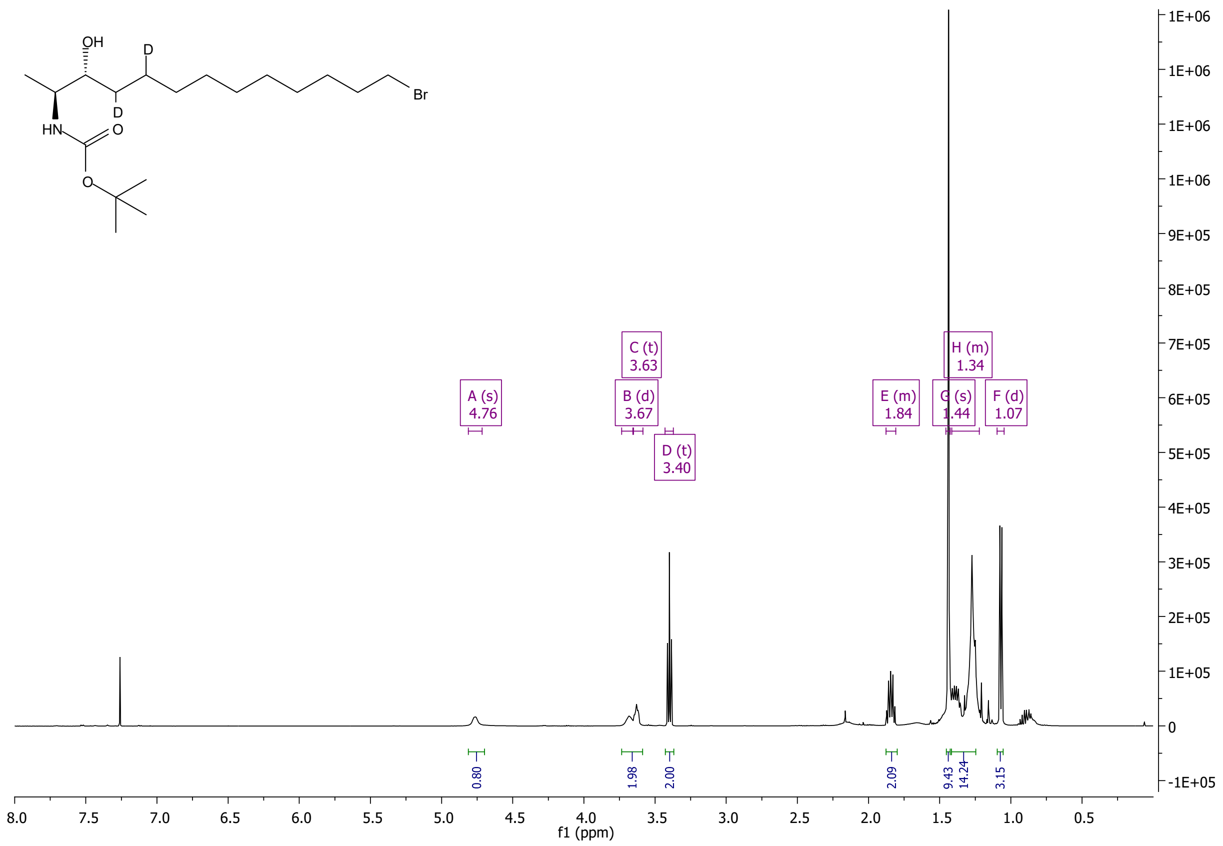

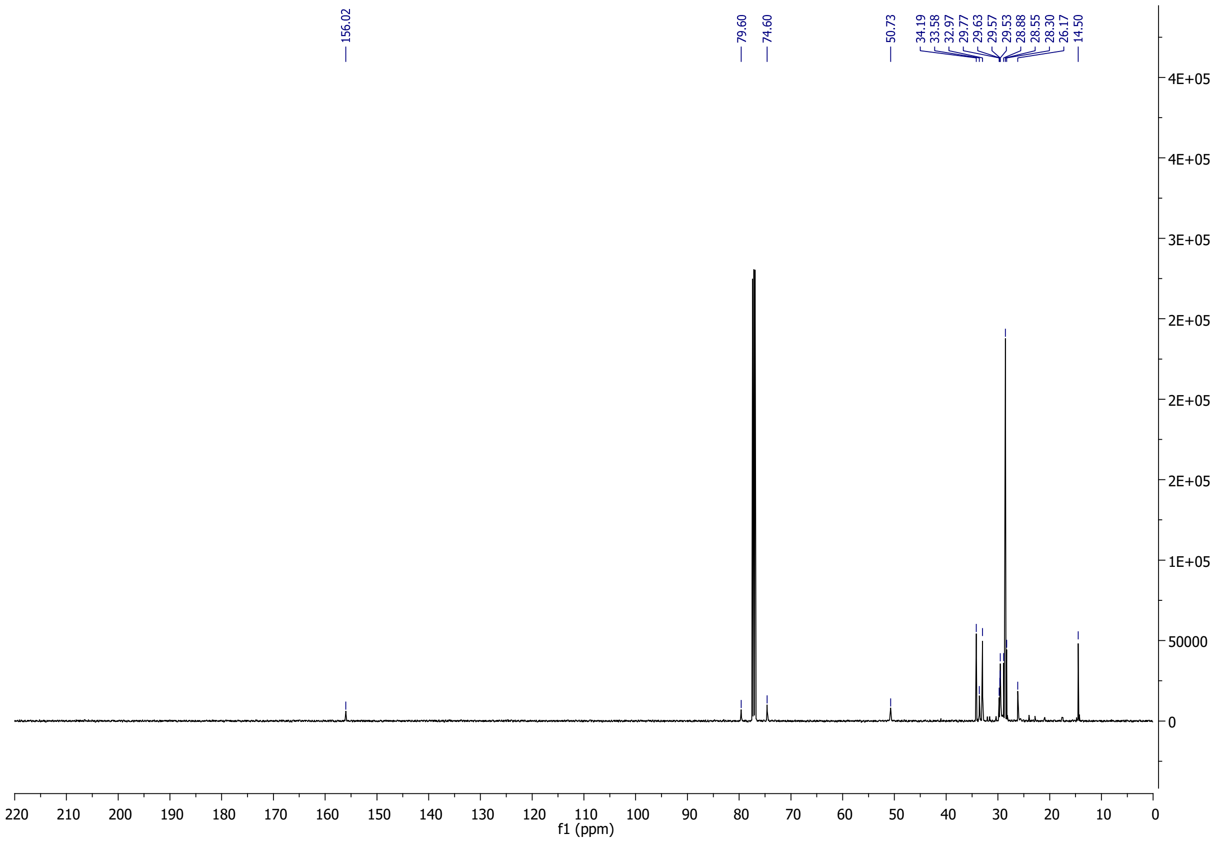

^{1}H- and ^{13}C- NMR Spectra for compound 21

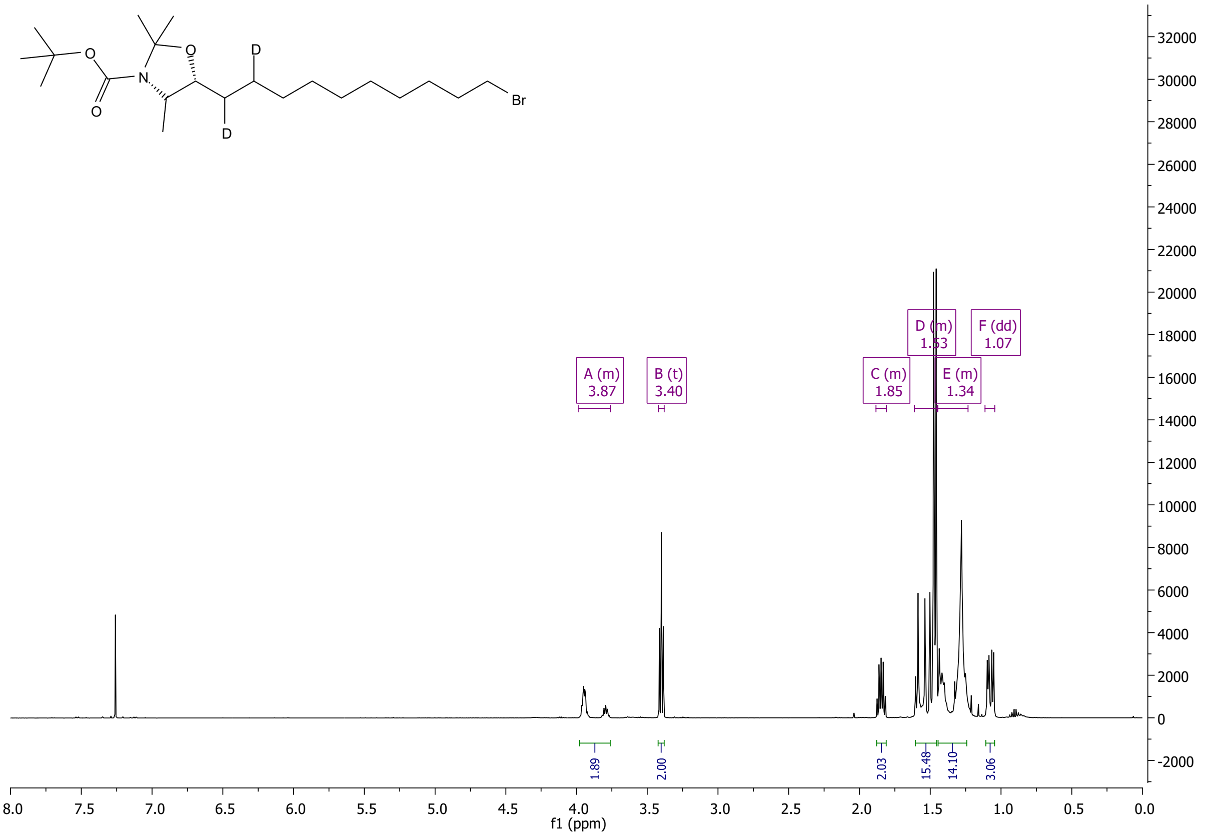

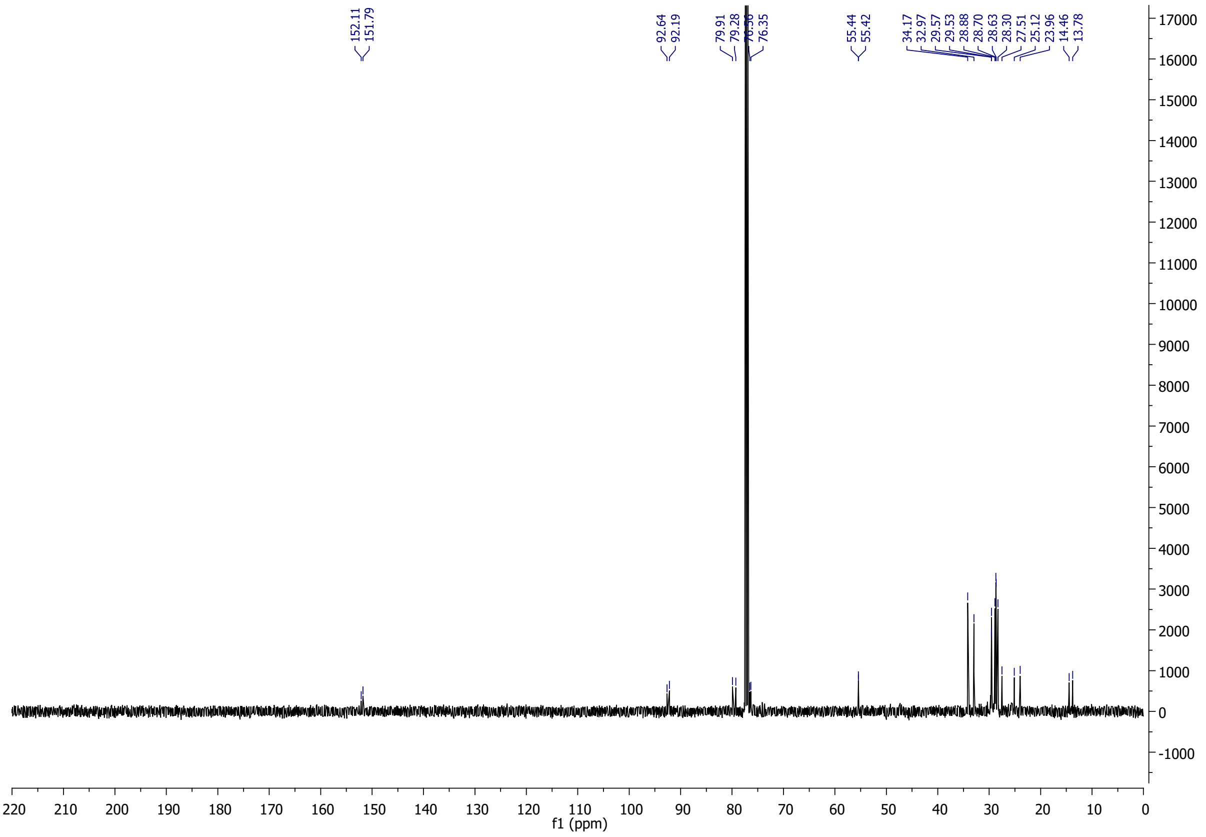

^{1}H- and ^{13}C- NMR Spectra for compound 22

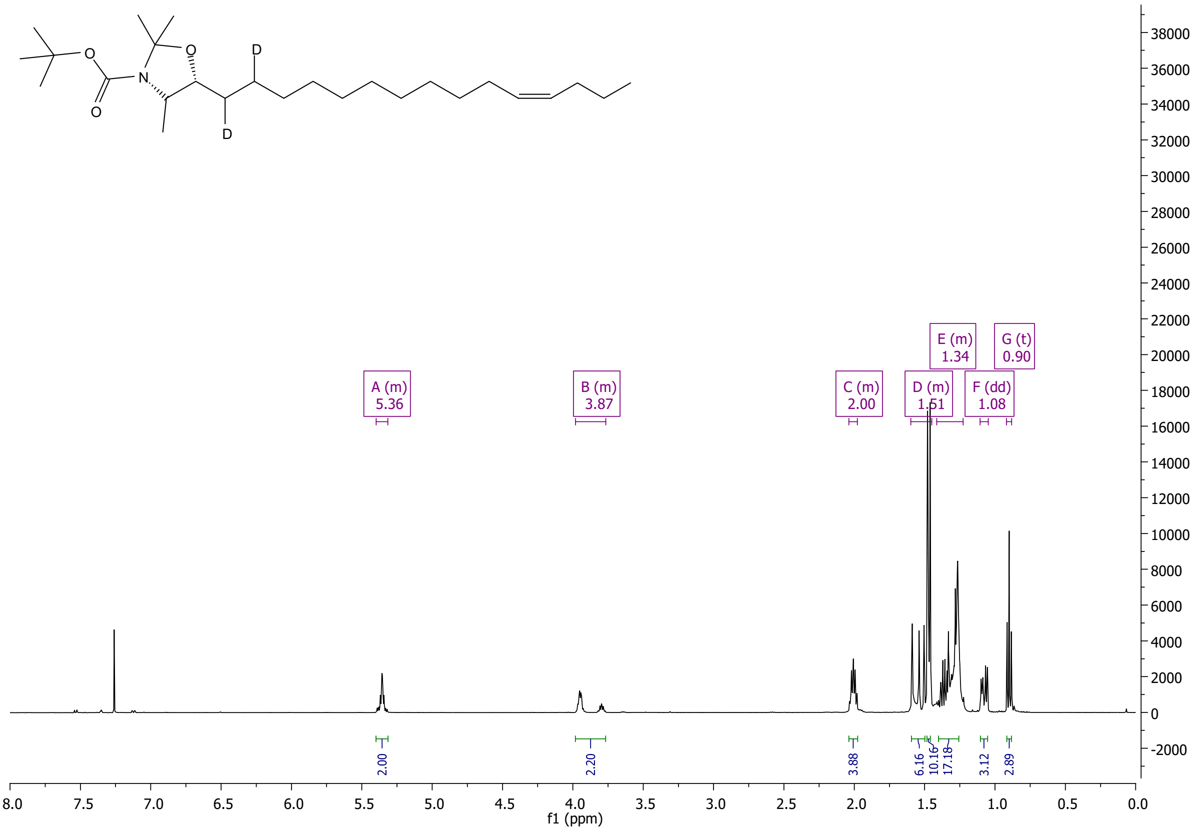

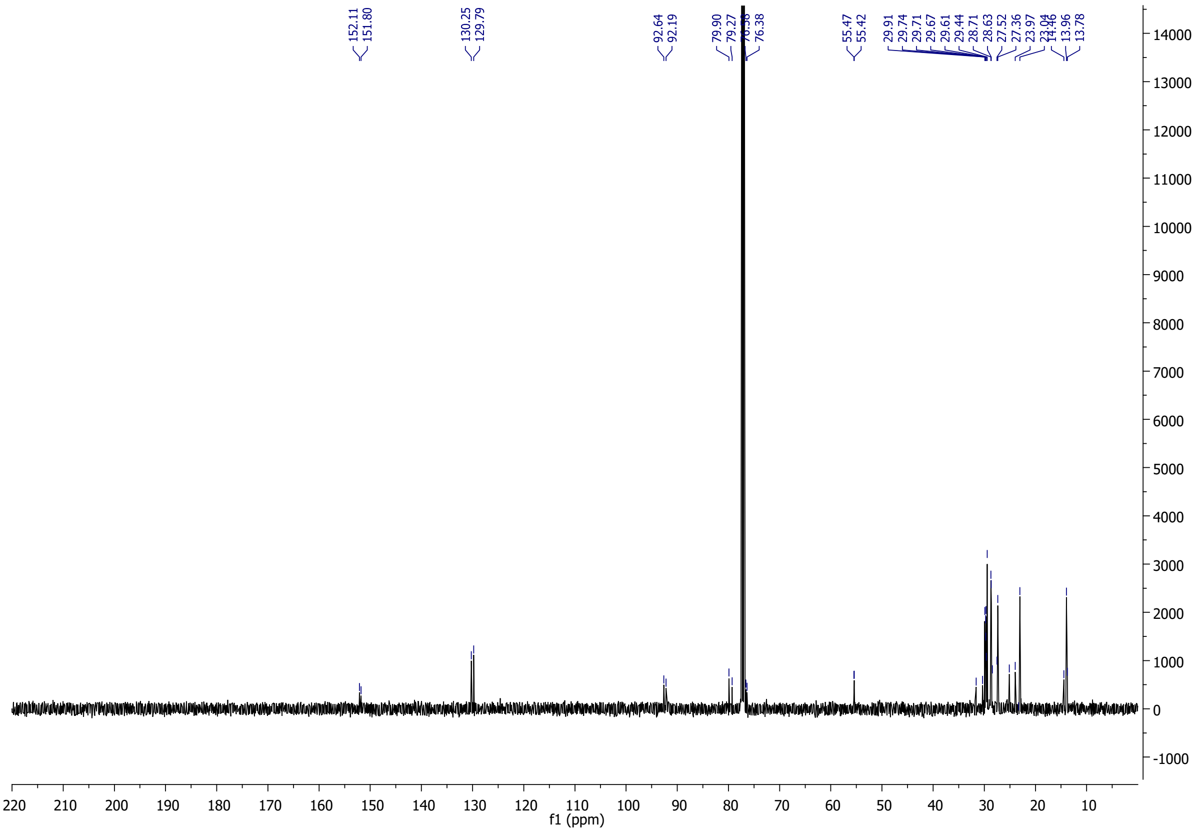

^{1}H- and ^{13}C- NMR Spectra for compound 24

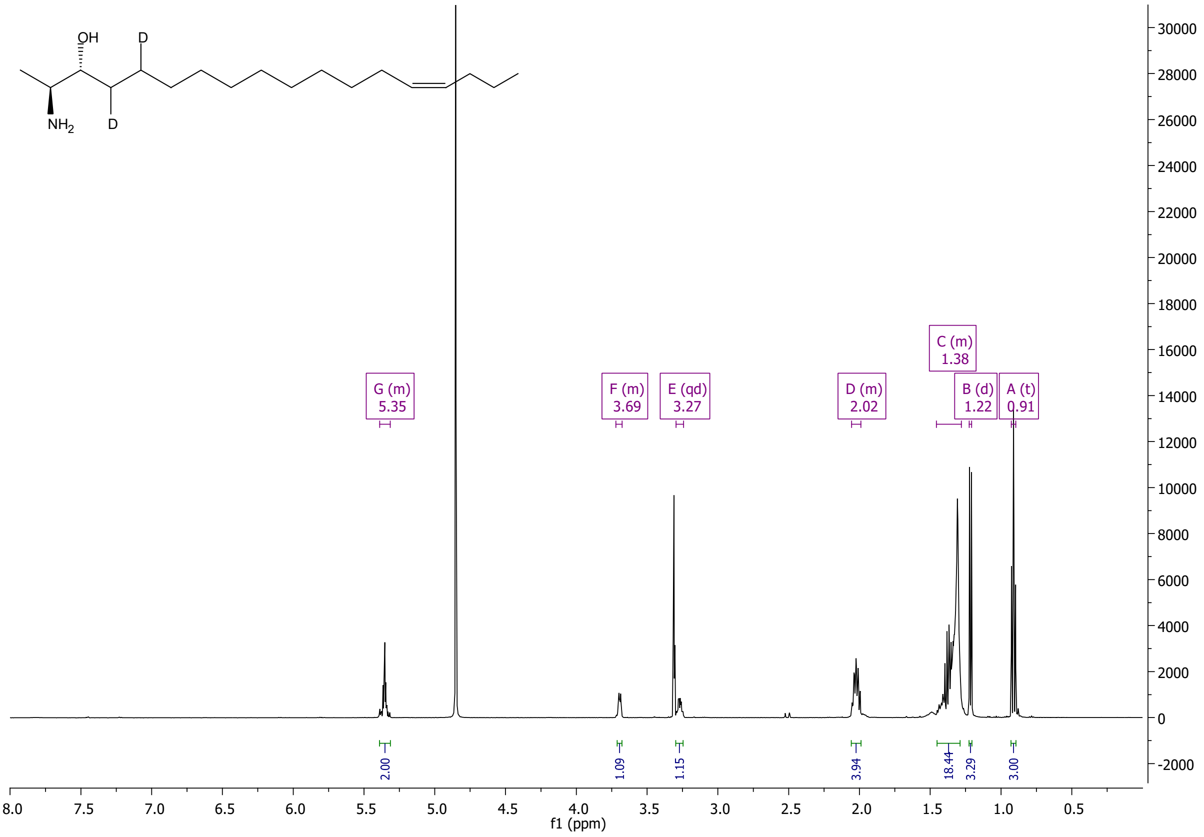

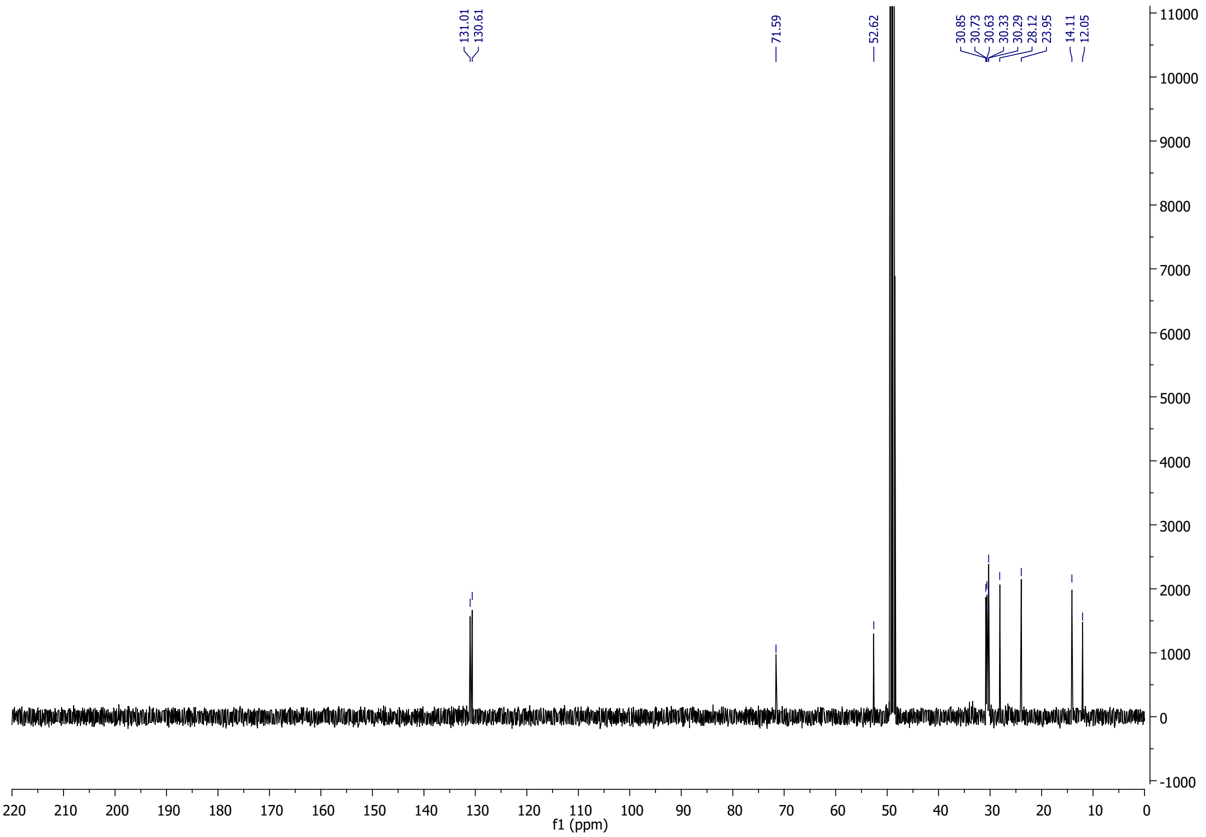

^{1}H- and ^{13}C- NMR Spectra for compound 25

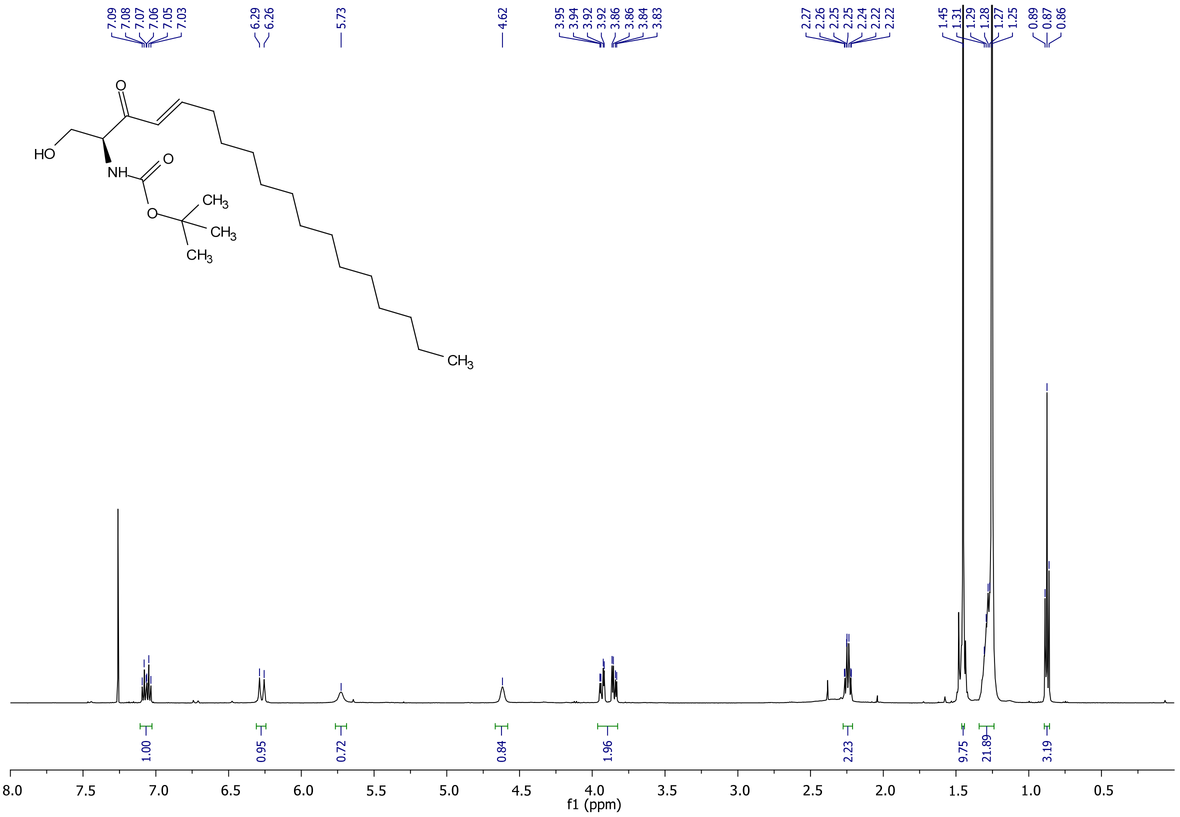

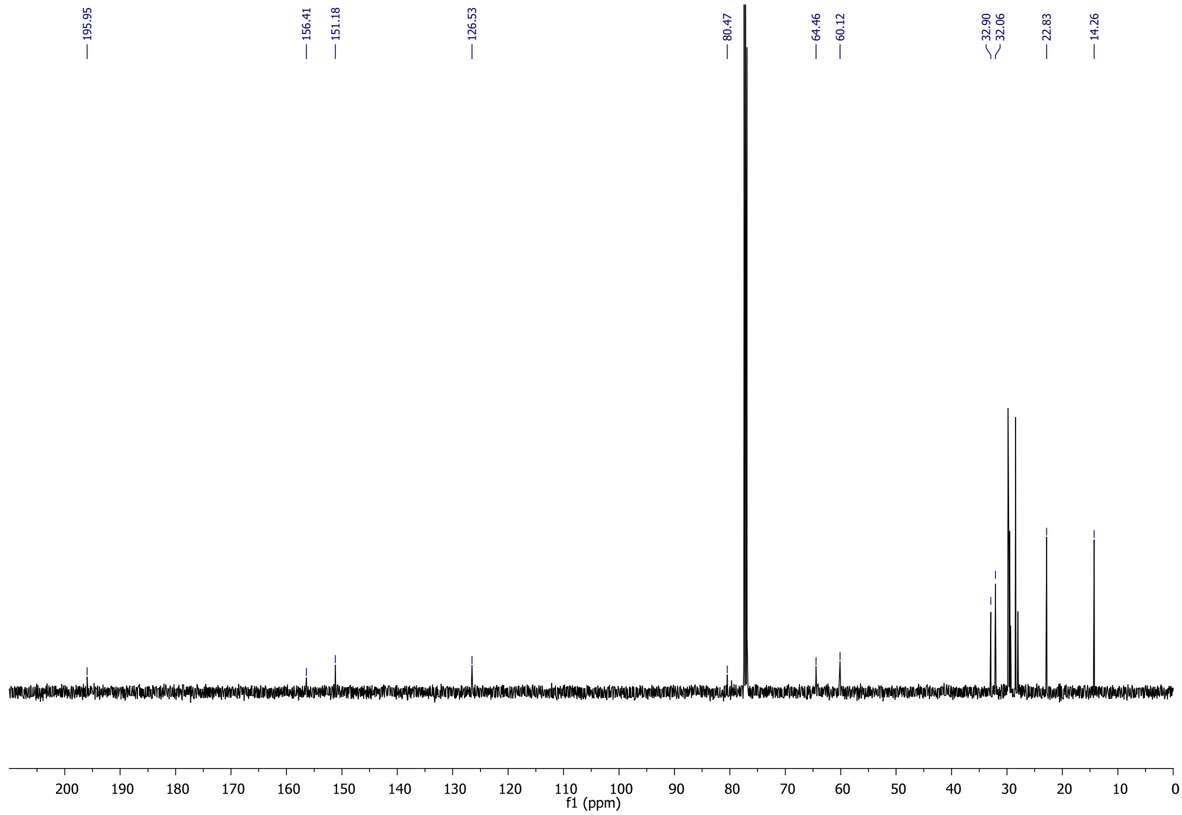

^{1}H- and ^{13}C- NMR Spectra for compound 29

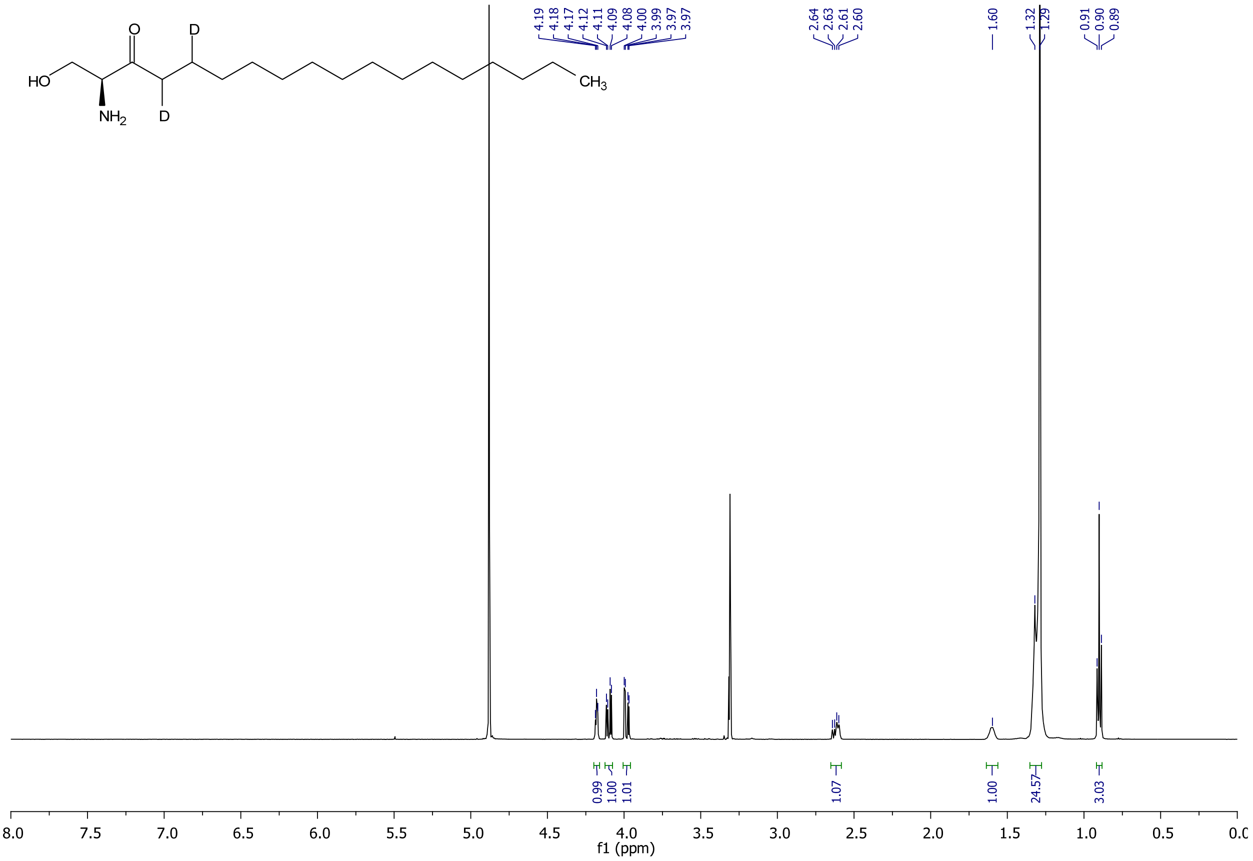

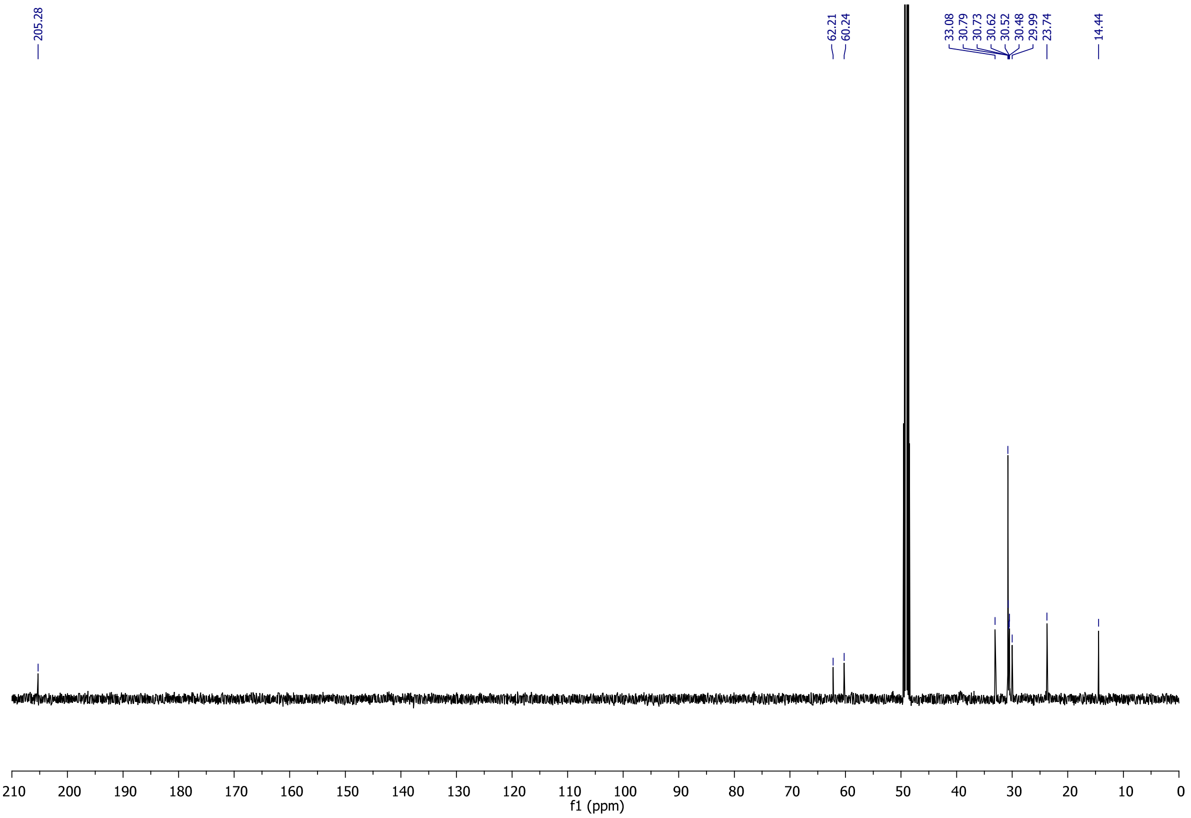

^{1}H- and ^{13}C- NMR Spectra for compound 30

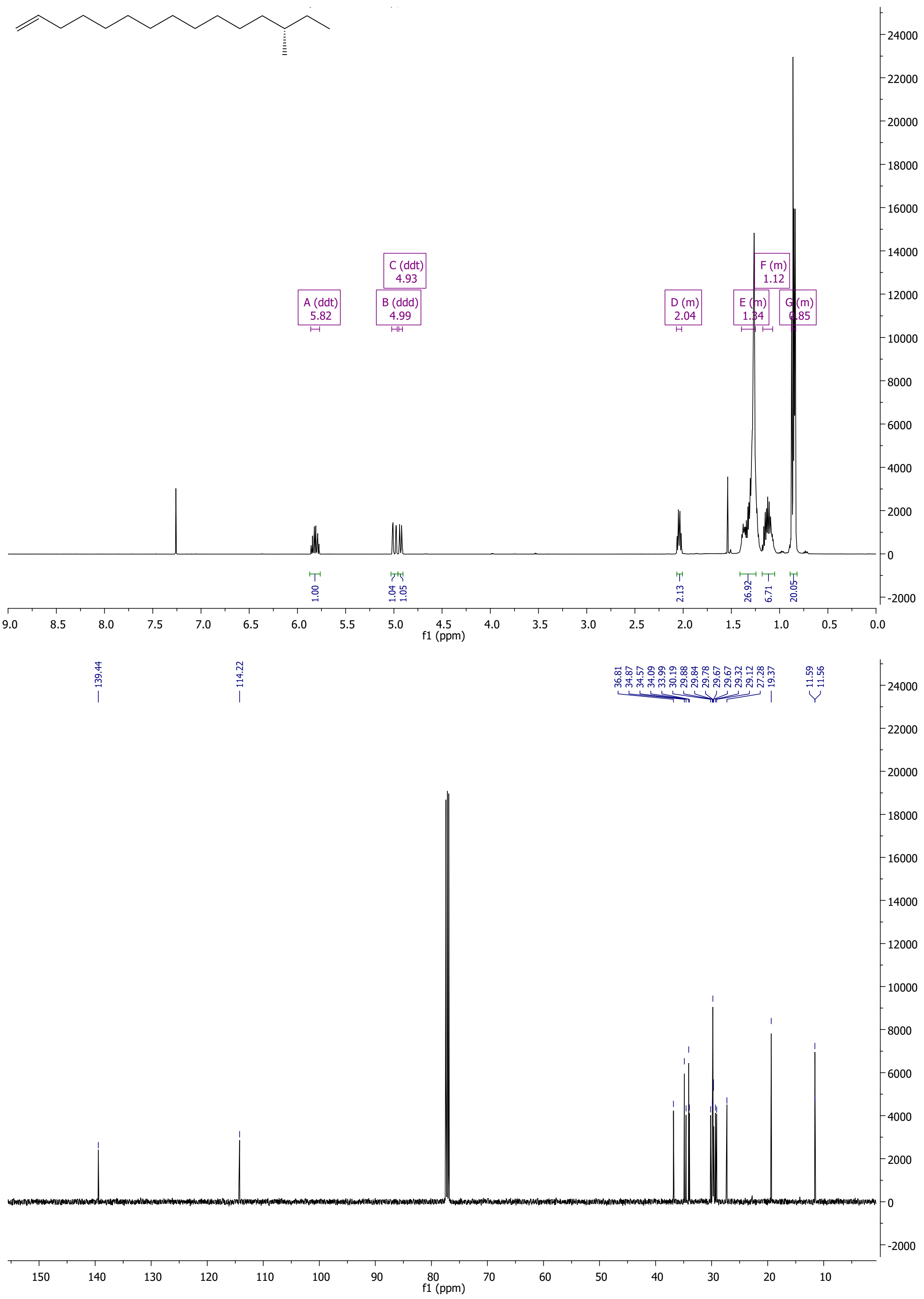

^{1}H- and ^{13}C- NMR Spectra for compound 39

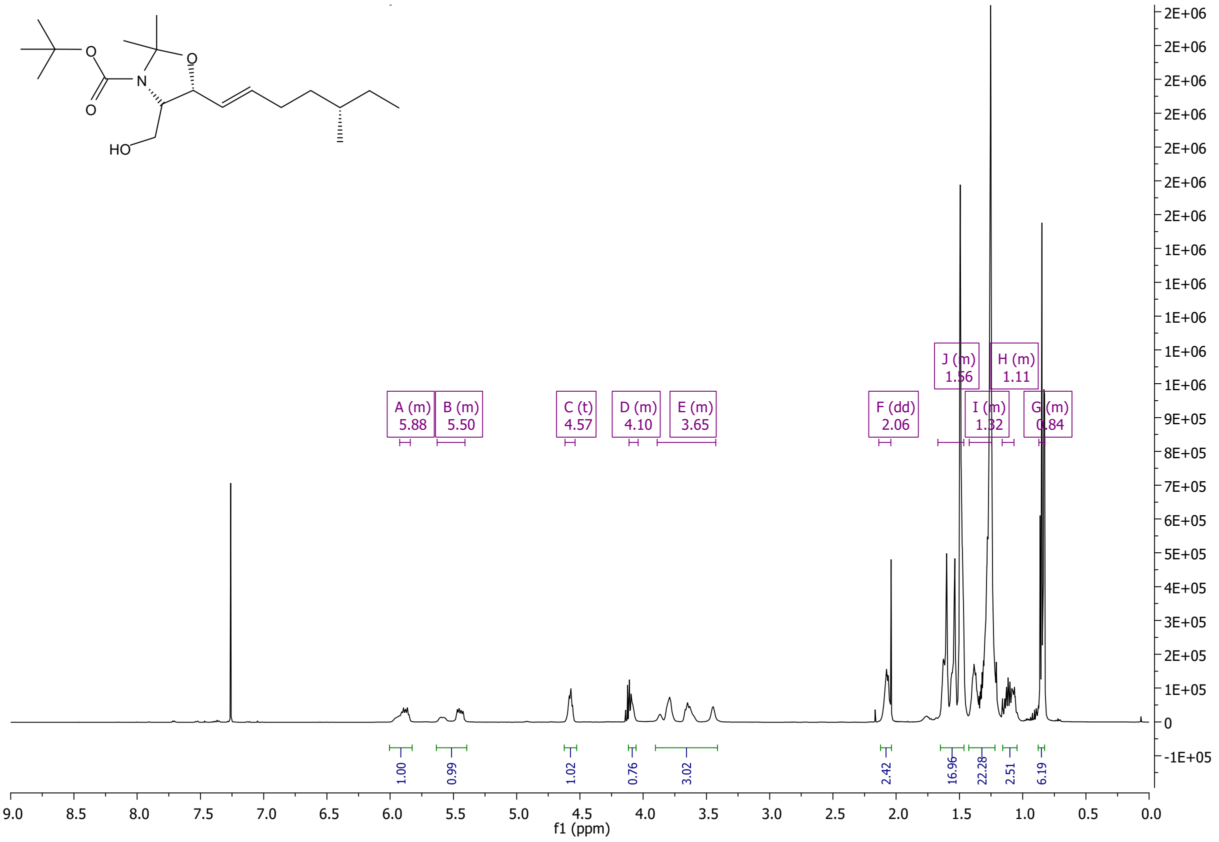

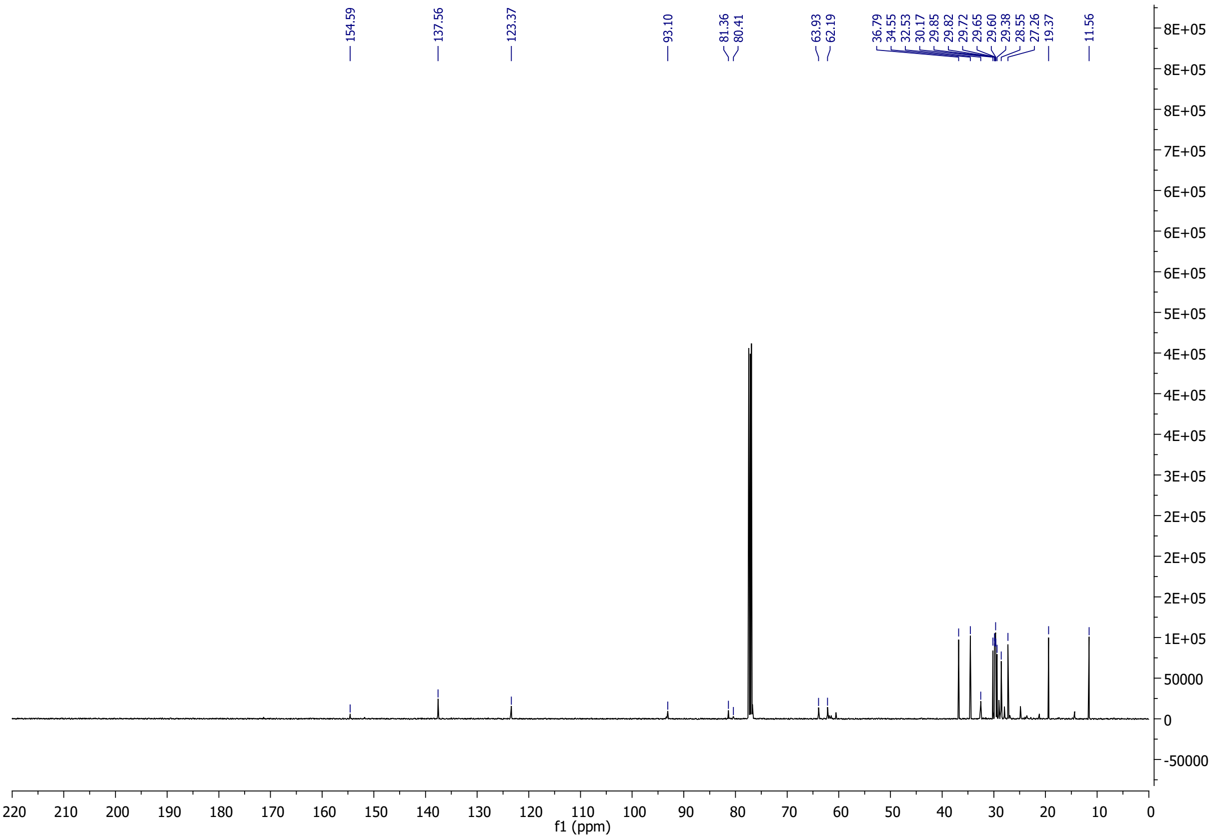

^{1}H- and ^{13}C- NMR Spectra for compound 41

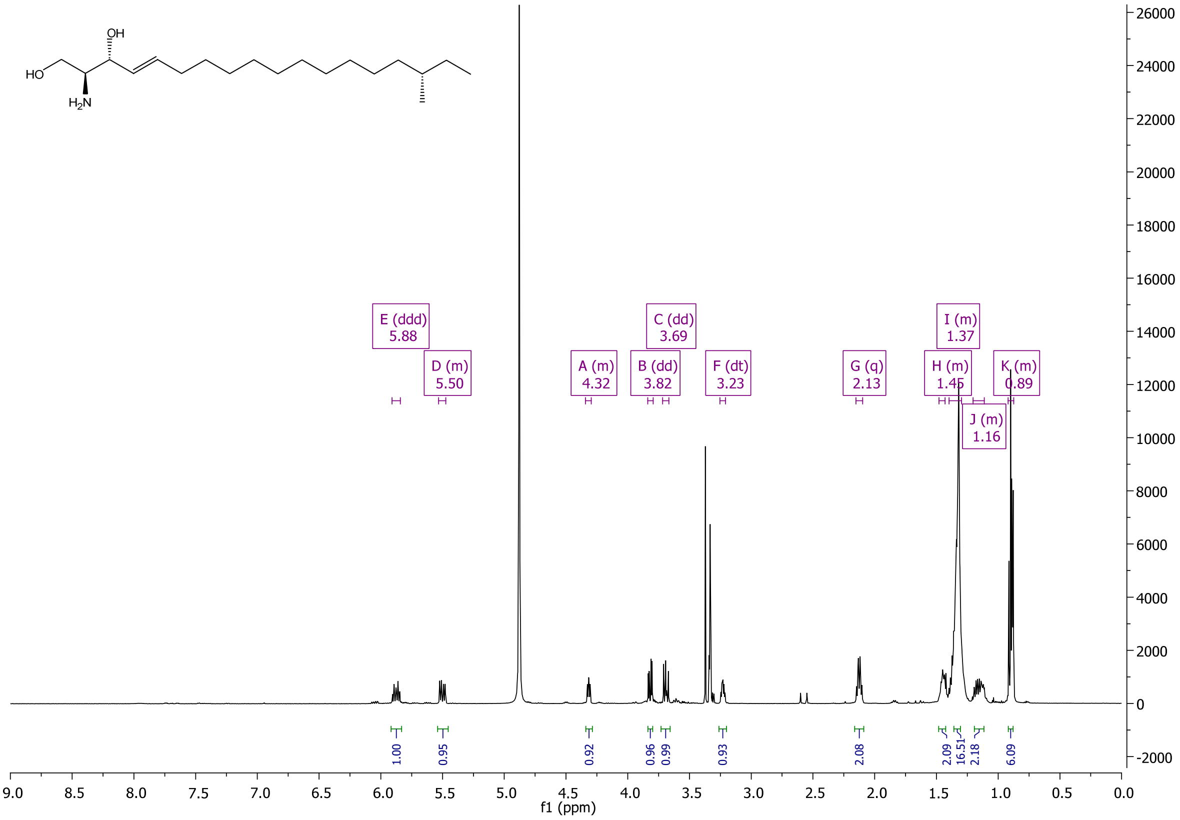

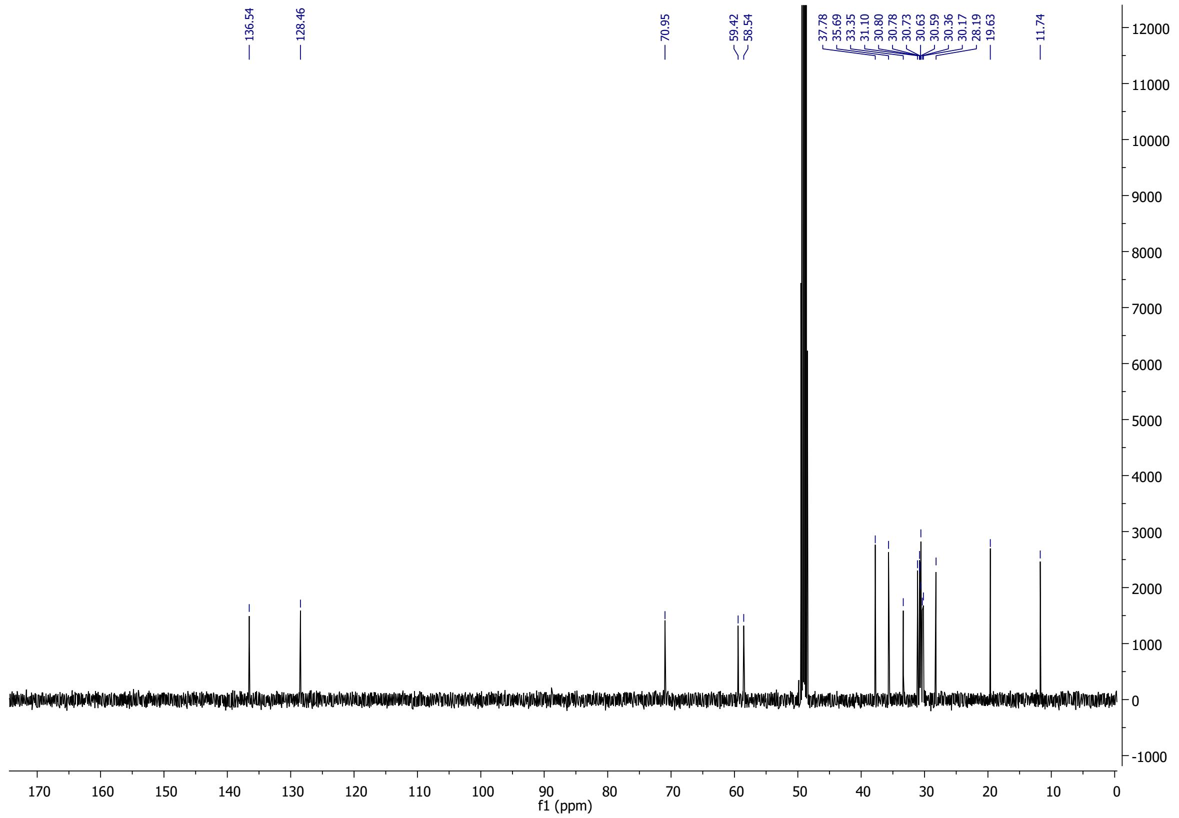

^{1}H- and ^{13}C- NMR Spectra for compound 42

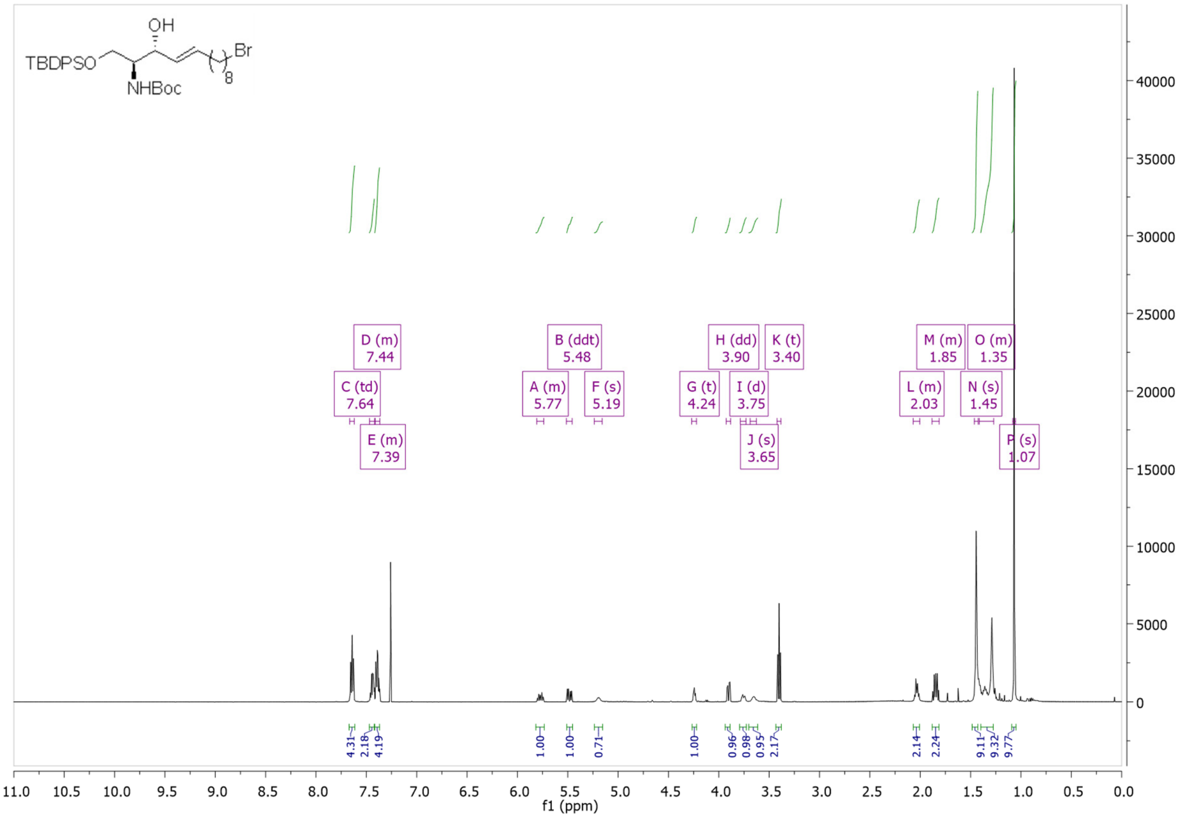

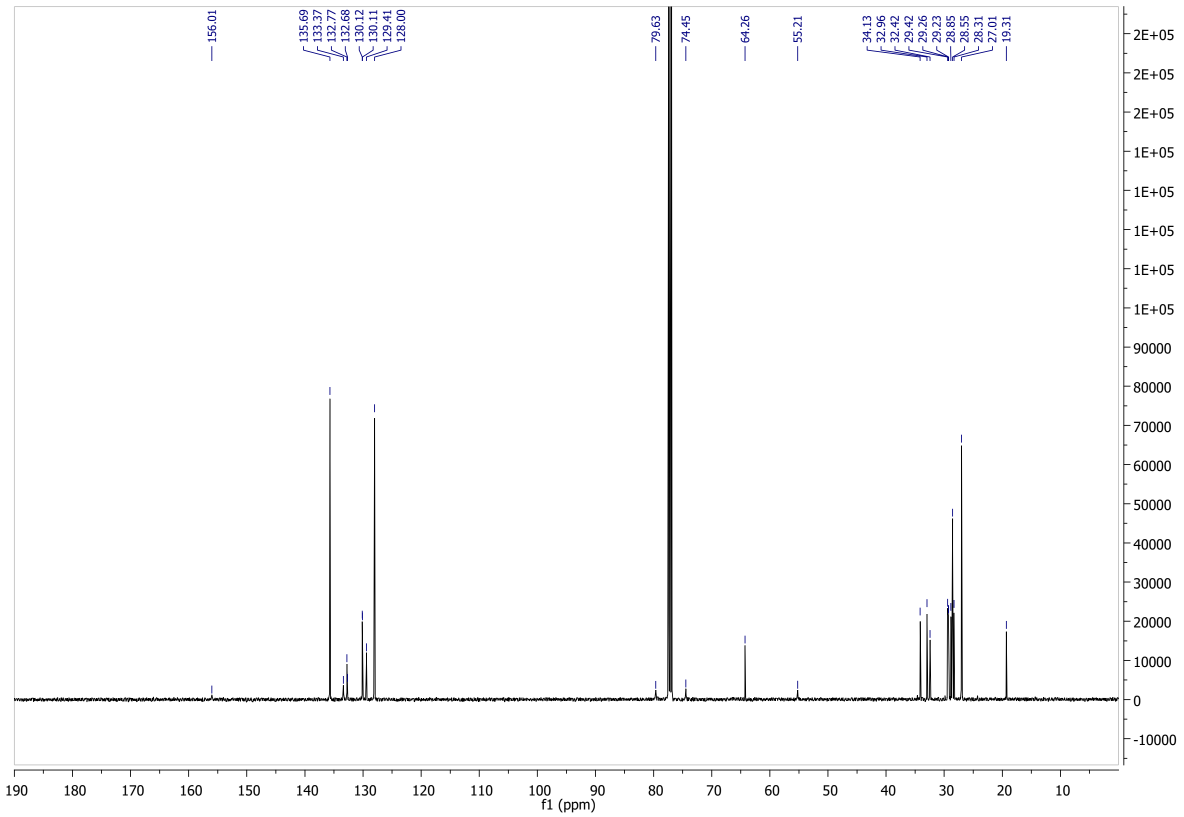

^{1}H- and ^{13}C- NMR Spectra for compound 44

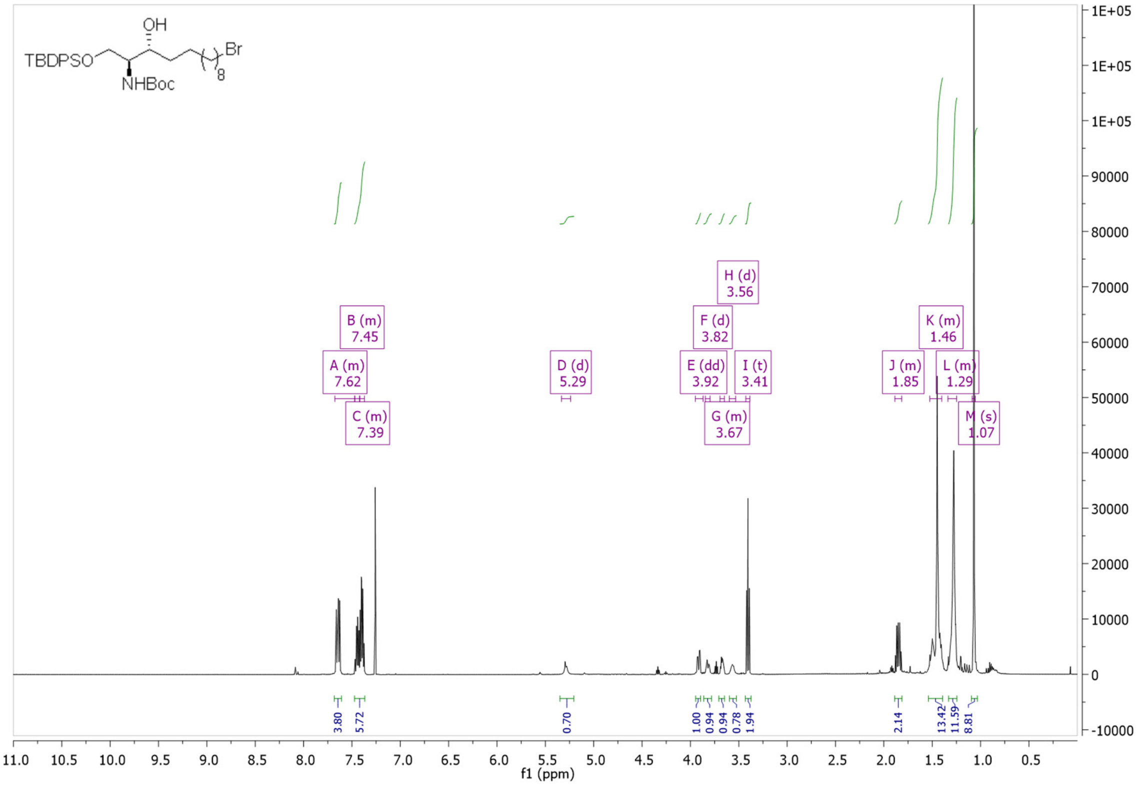

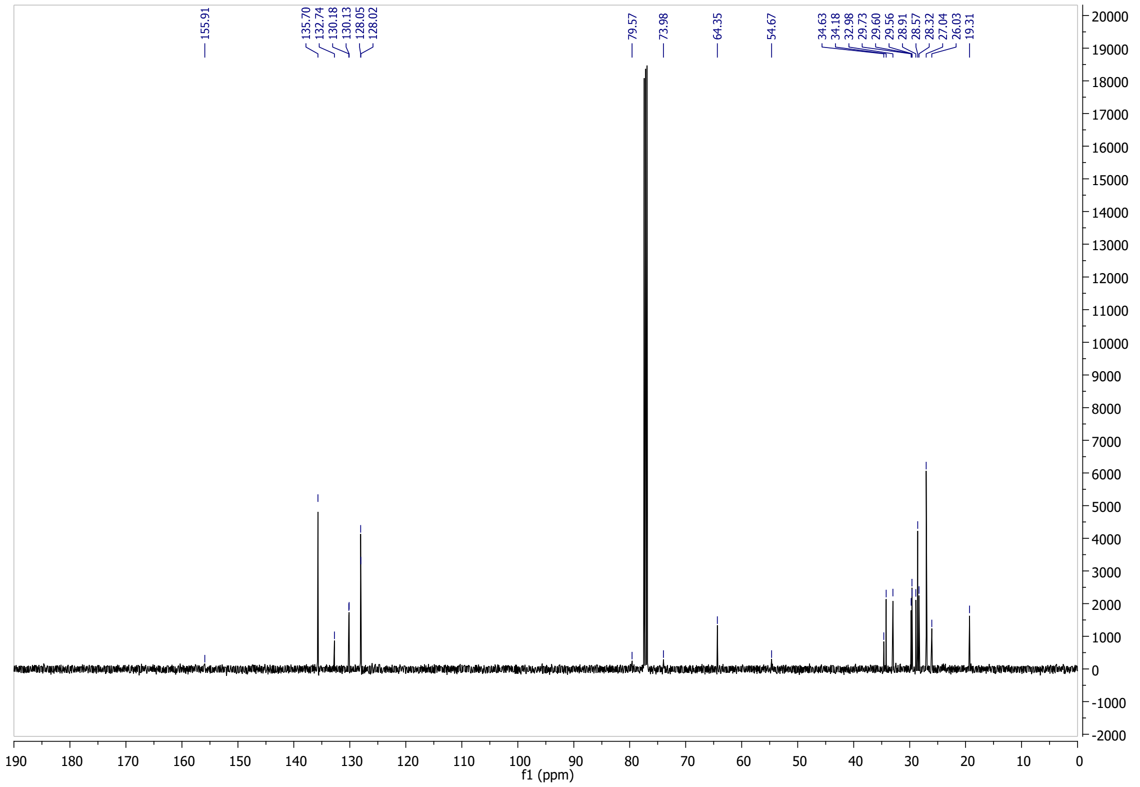

^{1}H- and ^{13}C- NMR Spectra for compound 45

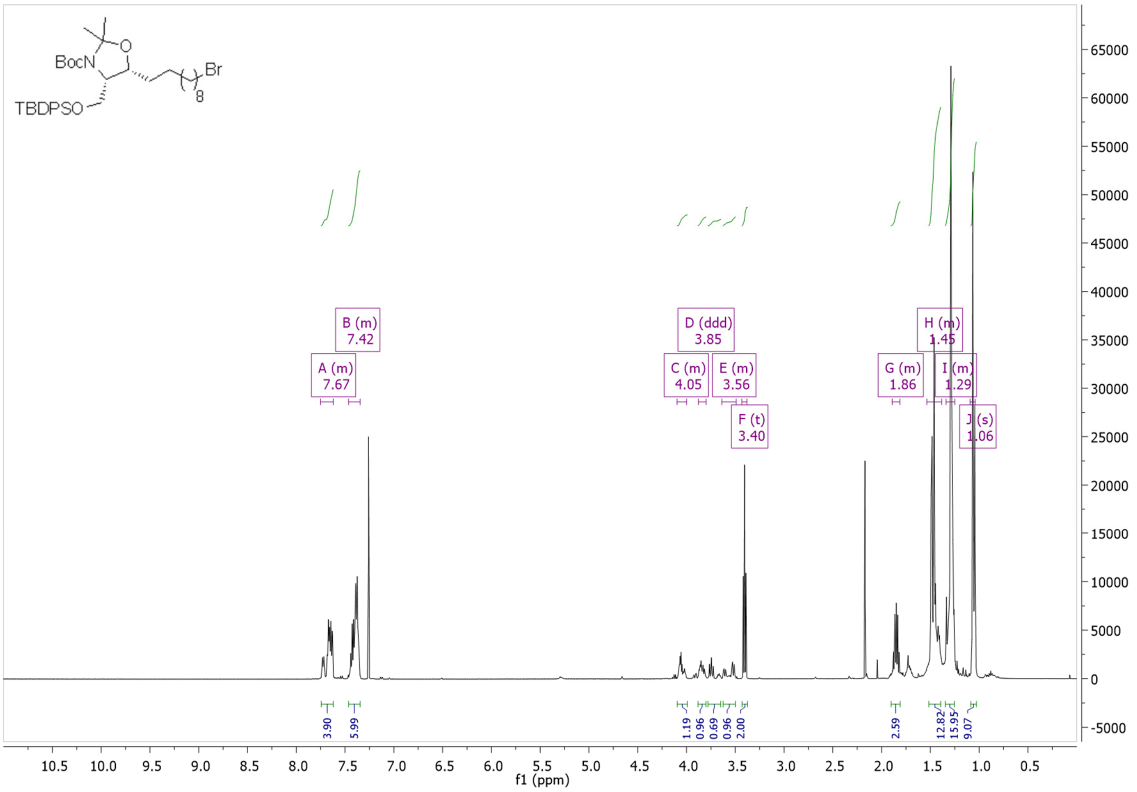

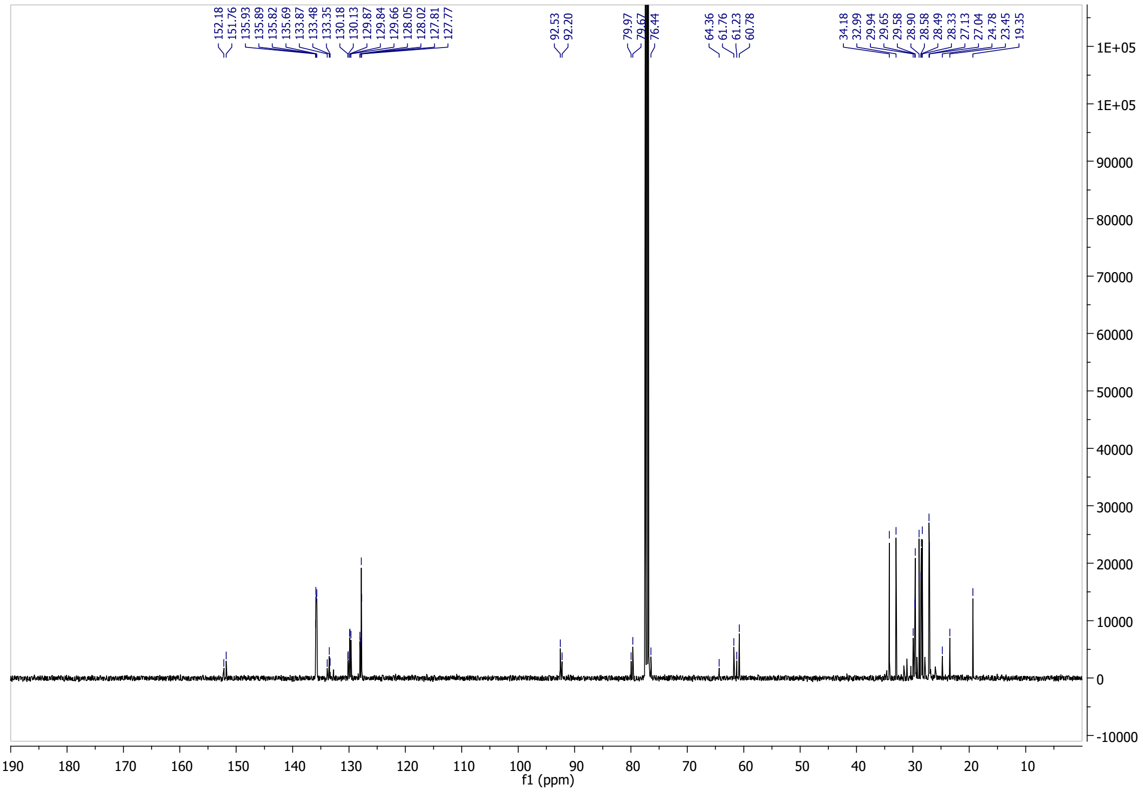

^{1}H- and ^{13}C- NMR Spectra for compound 46

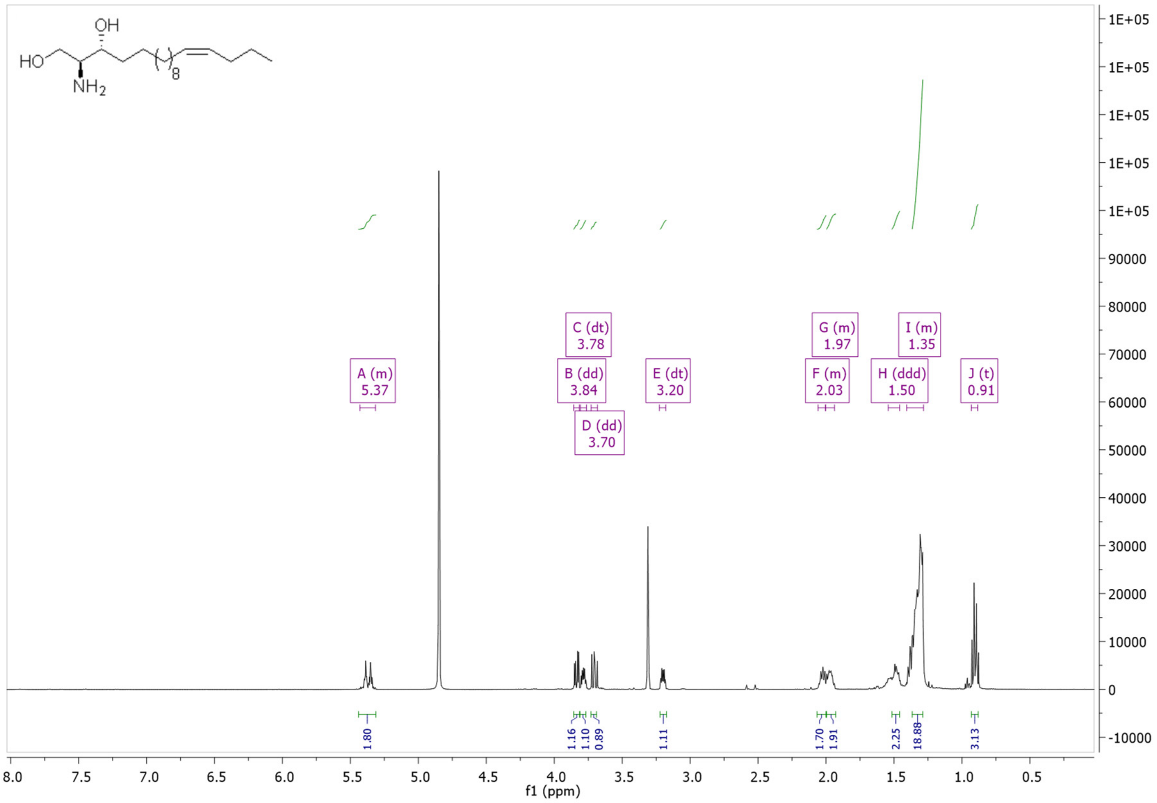

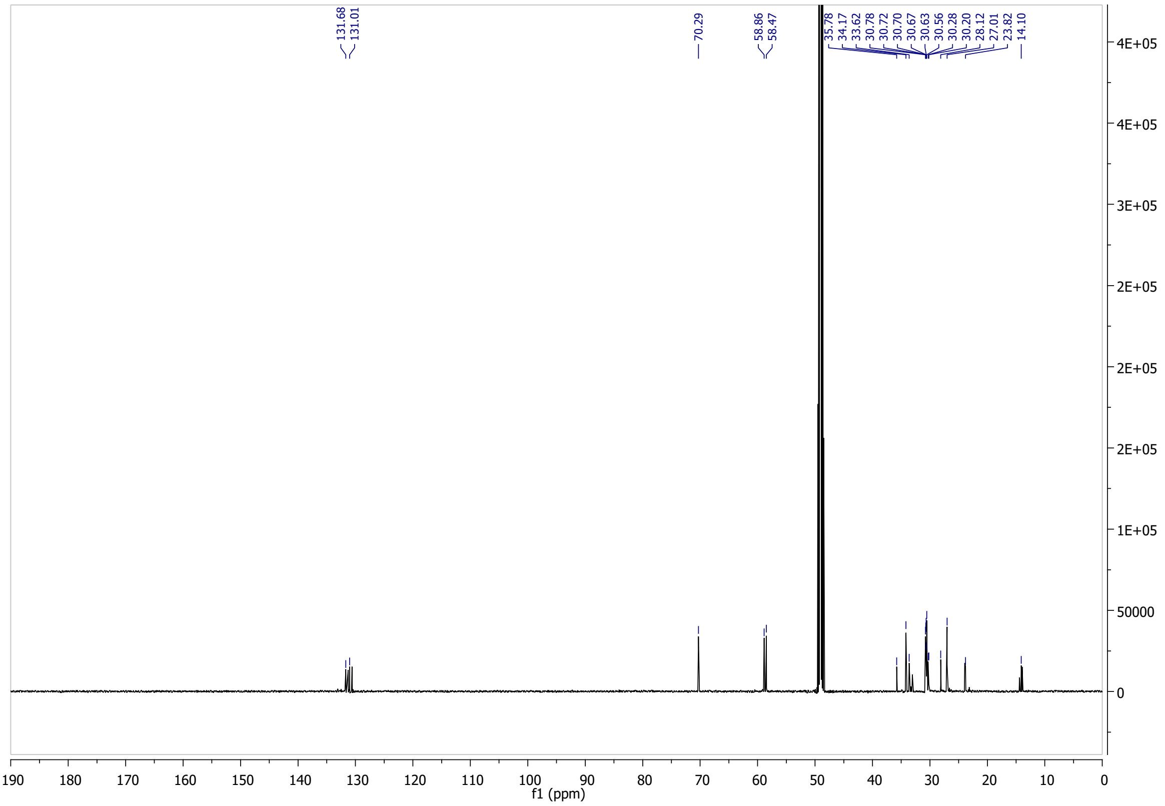

^{1}H- and ^{13}C- NMR Spectra for compound 49

4 References

1. Steiner, R.; Saied, E.M.; Othman, A.; Arenz, C.; Maccarone, A.T.; Poad, B.L.J.; Blanksby, S.J.; Eckardstein, A. von; Hornemann, T. Elucidating the Chemical Structure of Native 1-Deoxysphingosine. *J. Lipid Res.* **2016**, *57*, 1194–1203, doi:10.1194/jlr.M067033.
2. Saied, E.M.; Banhart, S.; Bürkle, S.E.; Heuer, D.; Arenz, C. A Series of Ceramide Analogs Modified at the 1-Position with Potent Activity against the Intracellular Growth of Chlamydia Trachomatis. *Future Medicinal Chemistry* **2015**, *7*, 1971–1980, doi:10.4155/fmc.15.126.
3. Ren, J.; Saied, E.M.; Zhong, A.; Snider, J.; Ruiz, C.; Arenz, C.; Obeid, L.M.; Girnun, G.D.; Hannun, Y.A. Tsc3 Regulates SPT Amino Acid Choice in Saccharomyces Cerevisiae by Promoting Alanine in the Sphingolipid Pathway. *Journal of Lipid Research* **2018**, *59*, 2126–2139, doi:10.1194/jlr.M088195.
4. Saied, E.M.; Le, T.L.-S.; Hornemann, T.; Arenz, C. Synthesis and Characterization of Some Atypical Sphingoid Bases. *Bioorganic & Medicinal Chemistry* **2018**, *26*, 4047–4057, doi:10.1016/j.bmc.2018.06.031.
5. Mina, J.G.; Mosely, J.A.; Ali, H.Z.; Denny, P.W.; Steel, P.G. Exploring Leishmania MajorInositol Phosphorylceramide Synthase (LmjIPCS): Insights into the Ceramide Binding Domain. *Org. Biomol. Chem.* **2011**, *9*, 1823–1830, doi:10.1039/C0OB00871K.
6. Huang, W.; Ma, J.-Y.; Yuan, M.; Xu, L.-F.; Wei, B.-G. A Facile Approach to Trans-4,5-Pyrrolidine Lactam and Application in the Synthesis of Nemonapride and Streptopyrrolidine. *Tetrahedron* **2011**, *67*, 7829–7837, doi:10.1016/j.tet.2011.07.049.
7. Hansford, K.A.; Dettwiler, J.E.; Lubell, W.D. One-Pot Synthesis of Homoallylic Ketones from the Addition of Vinyl Grignard Reagent to Carboxylic Esters. *Org. Lett.* **2003**, *5*, 4887–4890, doi:10.1021/ol0359822.
8. Arfan, M.; Tahira, A.; Mannan, A.; Fatima, T. A Facile Approach to the Synthesis of Benzothiazoles from N-Protected Amino Acids. *Russ J Org Chem* **2020**, *56*, 292–297, doi:10.1134/S1070428020020190.
9. Zer Aviv, P.; Shubely, M.; Moskovits, Y.; Viskind, O.; Albeck, A.; Vertommen, D.; Ruthstein, S.; Shokhen, M.; Gruzman, A. A New Oxopiperazin-Based Peptidomimetic Molecule Inhibits Prostatic Acid Phosphatase Secretion and Induces Prostate Cancer Cell Apoptosis. *ChemistrySelect* **2016**, *1*, 4658–4667, doi:https://doi.org/10.1002/slct.201600987.
10. Cryle, M.J.; Matovic, N.J.; De Voss, J.J. Products of Cytochrome P450BioI (CYP107H1)-Catalyzed Oxidation of Fatty Acids. *Org. Lett.* **2003**, *5*, 3341–3344, doi:10.1021/ol035254e.
11. Lee, S.-H.; Kim, H.-J.; Kim, D.-H.; Chang, W.-S.; Vales, T.P.; Kim, J.-W.; Kim, K.-H.; Kim, J.-K. Thermo-Sensitive Nanogel-Laden Bicontinuous Microemulsion Drug-Eluting Contact Lenses. *Journal of Biomedical Materials Research Part B: Applied Biomaterials* **2019**, *107*, 1159–1169, doi:https://doi.org/10.1002/jbm.b.34209.
12. Kutsumizu, S.; Tokiwa, I.; Kawafuchi, A.; Miwa, Y.; Yamamura, Y.; Saito, K. Stabilization of the Bicontinuous Cubic Phase in Siloxane-Terminated Mesogens, 1,2-Bis[4′-(n-(Oligodimethylsiloxyl)Alkoxy)Benzoyl]Hydrazine. *Phys. Chem. Chem. Phys.* **2016**, *18*, 9013–9020, doi:10.1039/C6CP00622A.
13. Kitahara, T.; Aono, S.; Mori, K. Synthesis of Both the Enantiomers of Aseanostatin P5 (Sarcinic Acid), an Inhibitor of Myeloperoxidase Release, and Four Diastereomers of Aggreceride A, a Platelet Aggregation Inhibitor. *Bioscience, Biotechnology, and Biochemistry* **1995**, *59*, 78–82, doi:10.1271/bbb.59.78.
14. Mori, K.; Tashiro, T.; Zhao, B.; Suckling, D.M.; El-Sayed, A.M. Pheromone Synthesis. Part 243: Synthesis and Biological Evaluation of (3R,13R,1′S)-1′-Ethyl-2′-Methylpropyl 3,13-Dimethylpentadecanoate, the Major Component of the Sex Pheromone of Paulownia Bagworm, Clania Variegata, and Its Stereoisomers. *Tetrahedron* **2010**, *66*, 2642–2653, doi:10.1016/j.tet.2010.02.028.
